# A yeast-expressed RBD-based SARS-CoV-2 vaccine formulated with 3M-052-alum adjuvant promotes protective efficacy in non-human primates

**DOI:** 10.1126/sciimmunol.abh3634

**Published:** 2021-07-15

**Authors:** Maria Pino, Talha Abid, Susan Pereira Ribeiro, Venkata Viswanadh Edara, Katharine Floyd, Justin C. Smith, Muhammad Bilal Latif, Gabriela Pacheco-Sanchez, Debashis Dutta, Shelly Wang, Sanjeev Gumber, Shannon Kirejczyk, Joyce Cohen, Rachelle L. Stammen, Sherrie M. Jean, Jennifer S. Wood, Fawn Connor-Stroud, Jeroen Pollet, Wen-Hsiang Chen, Junfei Wei, Bin Zhan, Jungsoon Lee, Zhuyun Liu, Ulrich Strych, Neeta Shenvi, Kirk Easley, Daniela Weiskopf, Alessandro Sette, Justin Pollara, Dieter Mielke, Hongmei Gao, Nathan Eisel, Celia C. LeBranche, Xiaoying Shen, Guido Ferrari, Georgia D. Tomaras, David C. Montefiori, Rafick P. Sekaly, Thomas H. Vanderford, Mark A. Tomai, Christopher B. Fox, Mehul S. Suthar, Pamela A. Kozlowski, Peter J. Hotez, Mirko Paiardini, Maria Elena Bottazzi, Sudhir Pai Kasturi

**Affiliations:** 1 Division of Microbiology and Immunology, Yerkes National Primate Research Center, Emory University, 954, Gatewood Rd, Atlanta, GA 30329, USA.; 2 Department of Pathology and Laboratory Medicine, School of Medicine, Emory University, Atlanta, GA 30329, USA.; 3 Emory Vaccine Center at Emory University, 954, Gatewood Rd, Atlanta, GA 30329, USA.; 4 Centers for Childhood Infections and Vaccines, Children’s Healthcare of Atlanta, and Department of Pediatrics, Emory University, Atlanta, GA 30322, USA.; 5 Department of Microbiology, Immunology, and Parasitology, Louisiana State University Health Sciences Center, New Orleans, LA 70112, USA.; 6 Division of Pathology, Yerkes National Primate Research Center, Emory University, Atlanta, GA 30329, USA.; 7 Division of Animal Resources, Yerkes National Primate Research Center, Emory University, Atlanta, GA 30329, USA.; 8 Texas Children’s Center for Vaccine Development, Houston, TX 77030, USA.; 9 Department of Pediatrics, Molecular Virology and Microbiology, National School of Tropical Medicine, Baylor College of Medicine, Houston, TX 77030, USA.; 10 Department of Biostatistics and Bioinformatics, Emory University, Atlanta, GA 30322, USA.; 11 Center for Infectious Disease and Vaccine Research, La Jolla Institute for Immunology (LJI), La Jolla, CA 92037, USA.; 12 Department of Medicine, Division of Infectious Diseases and Global Public Health, University of California, San Diego (UCSD), La Jolla, CA 92037, USA.; 13 Department of Surgery, Duke Human Vaccine Institute, Duke University Medical Center, Durham, NC 27708, USA.; 14 3M Corporate Research Materials Laboratory, St. Paul, MN 55144-1000, USA.; 15 Infectious Disease Research Institute, Seattle, WA 98109, USA.

## Abstract

Ongoing severe acute respiratory syndrome coronavirus–2 (SARS-CoV-2) vaccine development is focused on identifying stable, cost-effective, and accessible candidates for global use, specifically in low- and middle-income countries. Here, we report the efficacy of a rapidly scalable, novel yeast-expressed SARS-CoV-2–specific receptor binding domain (RBD)–based vaccine in rhesus macaques. We formulated the RBD immunogen in alum, a licensed and an emerging alum-adsorbed TLR-7/8-targeted, 3M-052-alum adjuvant. The RBD + 3M-052-alum–adjuvanted vaccine promoted better RBD binding and effector antibodies, higher CoV-2 neutralizing antibodies, improved T_H_1-biased CD4^+^ T cell reactions, and increased CD8^+^ T cell responses when compared with the alum-alone adjuvanted vaccine. RBD + 3M-052-alum induced a significant reduction of SARS-CoV-2 virus in the respiratory tract upon challenge, accompanied by reduced lung inflammation when compared with unvaccinated controls. Anti-RBD antibody responses in vaccinated animals inversely correlated with viral load in nasal secretions and bronchoalveolar lavage (BAL). RBD + 3M-052-alum blocked a post–SARS-CoV-2 challenge increase in CD14^+^CD16^++^ intermediate blood monocytes, and fractalkine, MCP-1 (monocyte chemotactic protein–1), and TRAIL (tumor necrosis factor–related apoptosis-inducing ligand) in the plasma. Decreased plasma analytes and intermediate monocyte frequencies correlated with reduced nasal and BAL viral loads. Last, RBD-specific plasma cells accumulated in the draining lymph nodes and not in the bone marrow, contrary to previous findings. Together, these data show that a yeast-expressed, RBD-based vaccine + 3M-052-alum provides robust immune responses and protection against SARS-CoV-2, making it a strong and scalable vaccine candidate.

## Adjuvant adds protection to COVID-19 vaccine

Despite vaccinations against severe acute respiratory syndrome coronavirus–2 (SARS-CoV-2) being rolled out globally, it is important to consider the next generation of SARS-CoV-2 vaccines and adjuvants that might increase protection. Here, Pino *et al*. vaccinated rhesus macaques (RM) with a yeast-expressed RBD-based SARS-CoV-2 vaccine adjuvanted with 3M-052 (TLR7/8 agonist)–alum, previously shown to induce better immune responses against human immunodeficiency virus. Comparing this RBD-based vaccine adjuvanted with 3M-052-alum to the one adjuvanted only with alum, RMs were more protected from a SARS-CoV-2 challenge. This amplified protection correlated to increased SARS-CoV-2–specific antibody and T cell responses in the blood and the nasal mucosa. Thus, using the adjuvant 3M-052-alum may be a way to improve the efficacy of SARS-CoV-2 vaccines moving forward.

## Teaser

An adjuvanted monomeric RBD immunogen induces immunogenicity and protective immunity against SARS-CoV-2 in rhesus macaques.

## INTRODUCTION

Infection by beta coronaviruses (CoVs) in humans results in symptoms ranging from mild, caused by common cold–causing strains, to severe acute respiratory syndrome (SARS), caused by select highly virulent strains leading to death in many cases (*1*). Coronavirus disease 2019 (COVID-19) is a respiratory infectious disease caused by SARS-CoV-2, a new CoV first reported in Wuhan, China, in December 2019 (*2*–*4*). Because of rapid worldwide spread, the World Health Organization (WHO) declared COVID-19 a pandemic in March 2020 with more than 175 million cases now and greater than 3.5 million fatalities globally (*5*). An effective SARS-CoV-2 vaccine is the best way to stem the pandemic (*6*). A historic feat has been achieved in vaccinology by rapid design, testing, and approval (<~1 year) of several SARS-CoV-2 vaccines for use in select countries (*7*). However, there is a continued need for expanding the availability of safe, cost-effective, and scalable candidates for distribution in developing or low- to middle-income countries (*8*). In addition, there is an urgent need to improve cellular and molecular insights into SARS-CoV-2 vaccine–induced protective immunity in preclinical studies, preferentially in non-human primates (NHPs) (*7*, *9*).

SARS-CoV-2 infects host cells by binding of its surface transmembrane spike (S) protein to angiotensin-converting enzyme 2 (ACE2) receptors predominantly present on type II pneumocytes in the host respiratory mucosa (*10*). Thus, S protein is the lead target for vaccine design (*11*). Immunogens stabilized in their prefusion confirmation (*12*, *13*) elicit strong virus-neutralizing activity upon vaccination (*14*, *15*). The receptor binding domain (RBD) in the S protein represents the most dominant target for neutralizing SARS-CoV-2 (*16*, *17*) and RBD-targeted binding antibodies (Abs) highly correlate with virus-neutralizing activity (*18*). Several epitopes within RBD contribute to neutralization; thus, both monomeric and oligomeric RBD vaccines are being tested (*16*). We have previously reported the protective efficacy of a yeast (*Pichia pastoris*)–expressed RBD-based vaccine against SARS-CoV in mice (*19*, *20*). The wide availability of yeast expression technology for hepatitis B vaccines in low- and middle-income nations highlights a significant advantage for rapid manufacture of yeast-based SARS-CoV-2 vaccines, helping meet the need for inexpensive and accessible COVID-19 vaccines (*8*).

Unlike live-attenuated virus–based vaccines, subunit vaccines are weakly immunogenic and require the use of adjuvants (*21*, *22*). Adjuvants substantially improve the quantity and quality of vaccine-induced immunity and can facilitate immunogen dose sparing. Pharmaceutically acceptable adjuvants are needed to strongly enhance Ab responses against SARS-CoV-2 protein immunogens. We recently reported that 3M-052, an emerging Toll-like receptor 7/8 (TLR-7/8) agonist formulated in polymer nanoparticles or adsorbed to alum (3M-052-alum), promotes the durability of HIV envelope (Env)–specific long-lived plasma cells (LLPCs) in rhesus macaques (RMs); this correlated with serum Ab responses (*23*). In contrast, alum alone failed to induce LLPCs, an important finding considering that >40 subunit vaccines containing aluminum salts have been licensed for use (*24*). In addition, vaccinating with 3M-052 and HIV-1 Env induced Ab response in the genital mucosa and promotes protection from viral challenge (*25*). As the respiratory mucosa is the primary port of entry of CoVs, inducing SARS-CoV-2–specific Ab responses in the serum and upper (URT) and lower respiratory tracts (LRT) is crucial. The induction of vaccine-specific LLPCs might support the longevity of such Ab responses. There is strong synergy between Ab and CD8^+^ T cell responses in promoting protective efficacy in NHPs against HIV-1 (*25*, *26*). These findings, along with the emergence of new variants of SARS-CoV-2 that may escape neutralization by Abs (*27*), further emphasize the need for vaccines capable of inducing balanced CD8^+^ T cell and humoral immunity against SARS-CoV-2.

Here, we report the immunogenicity and protective efficacy of a novel, yeast-expressed, SARS-CoV-2–derived RBD-219-WT–based (referred to as RBD immunogen in the study) (*28*) vaccine formulated with adjuvants in RMs. We specifically combined RBD with alum or alum-adsorbed 3M-052 (3M-052-alum), two clinically applicable adjuvants in inducing RBD-specific humoral and T cell immunity (*29*). We challenged naïve control and vaccinated animals with live SARS-CoV-2 via the intranasal (IN) and intratracheal (IT) routes (*30*) and saw that protective immunity was induced only by the RBD + 3M-052-alum vaccine, suggesting its promise as a SARS-CoV-2 vaccine candidate.

## RESULTS

### Study design and timeline

**Fig. 1.**
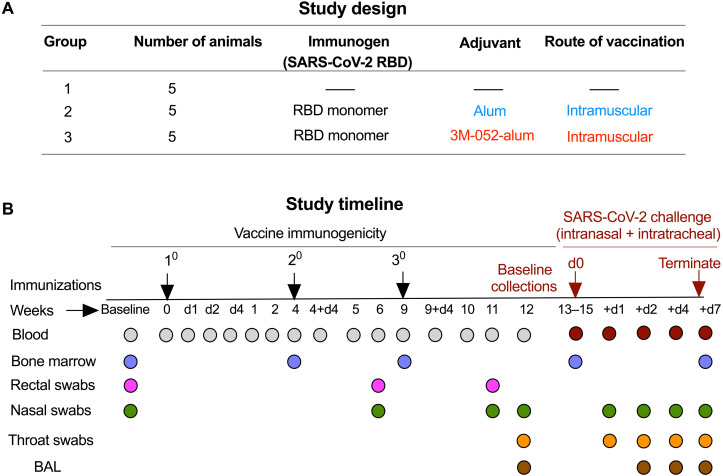
Study design and timeline. (**A**) Two groups of RMs (*n* = 5 per group) were immunized with a SARS-CoV-2–derived RBD monomer immunogen plus alum alone (group 2) or a combination of 3M-052 and alum (group 3). An additional *n* = 5 animals were included as naïve/unvaccinated controls (group 1) in the study when challenging with SARS-CoV-2. (**B**) Animals in groups 2 and 3 were vaccinated three times at time points indicated in the study timeline. Blood, BM aspirates, nasal, and rectal swabs were collected from animals at indicated time points for various assays described in the study. All vaccinated and the naïve control animals were challenged ~1 month after the third vaccination with ~2.3 × 10^5^ PFU of SARS-CoV-2, WA-1 strain via the IN and IT routes. VLs were quantified in the URT (nasal) and LRT (BAL) as well as in the throat. Animals were euthanized 1 week after challenge and anamnestic immune responses were quantified after necropsy.

The SARS-CoV-2 RBD (amino acid residues 332 to 549 of the spike protein S1 subunit) was cloned and expressed in the *P. pastoris* X-33 system (*20*) and homogeneity of the purified protein was demonstrated by SDS–polyacrylamide gel electrophoresis and size exclusion high-performance liquid chromatography (SE-HPLC). The glycosylated protein migrated as a single band on nonreducing gels and eluted as a single peak from the HPLC column (fig. S1). We had three groups of RMs (*n* = 5 per group) with one unvaccinated group (group 1), one group vaccinated with RBD + alum (group 2), and one with RBD + 3M-052-alum (group 3; Fig. 1A). The RBD immunogen was used at 100 μg and alum was used at 500 μg of Al^+3^ content identical to our recent studies with HIV-1 Env immunogens (*23*)and guided by use in pre-clinical studies and licensed vaccines (*31*, *32*). 3M-052 was used at 10 μg per dose guided by ongoing human studies (NCT04177355). All animals in groups 2 and 3 received three immunizations at weeks 0, 4, and 9. In view of reports of a wide range of Ab responses achieved by vaccine platforms with varying vaccine doses and timing of immunizations (*33*–*37*), we reasoned that a third vaccination could substantially improve the magnitude and quality (higher neutralizing activity and effector function) as previously observed with HIV-1 immunogens (*23*, *38*). Blood, mucosal (nasal and rectal) swabs, bronchoalveolar lavage (BAL), and bone marrow (BM) samples were collected as detailed (Fig. 1B). All animals were challenged with the Washington-1 (WA-1), SARS-CoV-2 strain via a combined IN and IT route of administration about 1 month after the third vaccination. Animals were staggered for viral challenge in three separate groups and were euthanized at day 7 or 8 after challenge. Blood, nasal, and throat swabs, as well as BAL samples were collected at pre- and postchallenge time points and at euthanasia (Fig. 1B).

### The RBD + 3M-052-alum vaccine induces robust humoral immunity in RMs

**Fig. 2.**
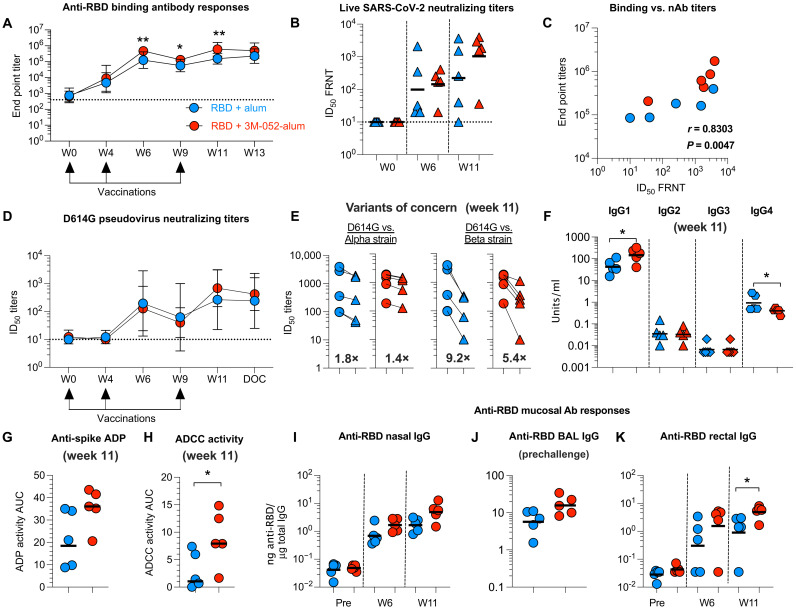
The RBD + 3M-052-alum vaccine induces robust humoral immunity in RMs. (**A**) Line graphs indicate end point titers of anti-RBD immunogen–specific binding Ab responses in RM serum. Geometric mean titers (GMT) with 95% confidence interval (CI) are shown (*n* = 5 per group). The dotted line at 400 indicates the start of serial dilution and a value assigned to animals with no background binding activity. W, week. (**B**) Live SARS-CoV-2 neutralization activity using a focus reduction neutralization titer (FRNT) assay as detailed in Materials and Methods. Horizontal bars indicate GMT. (**C**) Correlation between end point titers and live SARS-CoV-2 neutralization activity. (**D**) Line graphs indicate pseudovirus neutralization titers. All values below the limit of detection were assigned a value of 10 for plotting. GMT with 95% CI are shown (*n* = 5 per group). (**E**) Scatterplots indicate pseudovirus neutralization titers against the SARS-CoV-2 (WA-1) compared with the Alpha and Beta VOCs, respectively. Bold numbers indicate fold change in GMT. (**F**) Scatterplots indicate anti-RBD Ab IgG isotypes assayed at week 11. (**G**) Scatterplots show ADP activity in serum at week 11. (**H**) Scatterplots indicate Ab-dependent NK cell degranulation activity against target cells when expressing protein with the mutated G614. (**I**) Scatterplots indicate anti-RBD immunogen–specific binding Ab activity in nasal swabs. (**J**) Scatterplots show anti-RBD–specific binding Ab activity in BAL. Prechallenge refers to 5 days before SARS-CoV-2 challenge. (**K**) Graph indicates anti-RBD immunogen–specific binding Ab activity in rectal swabs. Horizontal lines in graphs (F) to (K) show the geometric mean. RMAs were used to test for significant statistical differences in Ab titers and pseudo neutralization titers (A and D) measured longitudinally over time as detailed in Materials and Methods. **P = 0.003 at week 6, *P = 0.02 at week 9, and **P = 0.002 at week 11 in (A). The difference in magnitude of Ab response in (B) and (F) to (K) was tested using a non-parametric two-tailed Mann-Whitney test using the GraphPad Prism software version 8.0. *P* < 0.05 was considered significant. *P = 0.0317 for IgG1 and *P* = 0.0476 for IgG4 in (F). *P = 0.0317 in (H) and (K). Spearman’s correlation analysis was used to compare the correlation between the magnitude of immune responses in (C). Spearman’s *r* and *P* values are indicated on the graphs.

We first investigated serological and mucosal humoral immunity induced by our vaccine. We observed onset of RBD binding Ab responses as early as 4 weeks after the first vaccination, which was then substantially increased (~26- to 30-fold) at week 6 and week 11 after the first and second boost vaccinations in both groups (Fig. 2A). Binding Ab responses were significantly higher in group 3 when compared with group 2 at indicated time points (Fig. 2A) and were persistent in both groups before challenge. We next compared and quantified binding Abs with RBD, whole spike (S), and nucleocapsid (N), which ensured recognition of the RBD in a more native S protein–associated form. The kinetics of RBD- and S-specific Ab responses (fig. S2, A and B) were identical, and there was no binding of Abs to N protein detected (fig. S2C). A correlation was observed between anti-RBD Ab responses (fig. S2D).

Live virus neutralizing titers were modest in all vaccinated animals after the second dose but were boosted to higher levels after the third vaccination (Fig. 2B). A positive correlation was found between RBD binding and live virus neutralizing activity (Fig. 2C). In addition, we assayed for neutralizing activity using the pseudovirus with the D614G mutation in the S protein found commonly in most circulating SARS-CoV-2 (*39*). Consistent with live virus neutralizing activity, pseudovirus neutralizing Abs (nAbs) were higher in both groups after the third vaccination. (Fig. 2D). We then screened for potential reduction in nAb responses against the spike-bearing pseudoviruses from the Alpha strain and the Beta strain, two SARS-CoV-2 variants of concern (VOCs) that emerged in the United Kingdom and South Africa in 2020, respectively (*27*, *40*). A marginal 1.8- and 1.4-fold reduction in nAb titer against the Alpha strain and a 9.2-fold and a 5.4-fold drop against the Beta strain were observed in serum of groups 2 and 3, respectively, at week 11 (Fig. 2E).

We then assayed for vaccine-induced Ab subclasses [immunoglobulin G (IgG) 1 to 4] at week 11 (*41*). A significantly higher anti-RBD IgG1 subclass response was induced in group 3 animals in contrast with significantly higher anti-RBD IgG4 isotype in group 2 animals, suggesting a skewing to T_H_1 and T_H_2 responses by the respective adjuvants (Fig. 2F). There was also a consistent trend of higher nonneutralizing Ab effector function in group 3 animals using an Ab-dependent phagocytosis (ADP) (Fig. 2G) and a spike protein–expressing cell Ab binding assay (fig. S2E) most likely contributed by the balance of Ab subclasses. Last, using a new natural killer (NK) cell–mediated degranulation assay (fig. S3) reflecting Ab-dependent cell cytotoxic (ADCC) potential, we observed significantly higher responses in serum of animals in group 3 when compared with group 2 (Fig. 2H), and these ADCC responses positively correlated with the binding Ab titers (fig. S2F).

We next assayed for vaccine-induced Ab responses in respiratory mucosa. We observed higher anti-RBD binding Ab responses in nasal secretions after the second vaccination at week 6, which continued to increase after the third vaccination most prominently in group 3 (Fig. 2I). IgG responses in nasal secretions positively correlated with live SARS-CoV-2 neutralization titers in serum (fig. S2G), suggesting a potential serum and not mucosal origin for these Abs. Anti-RBD binding Ab responses in BAL were modestly higher in group 3 animals versus group 2 (Fig. 2J) and correlating with live SARS-CoV-2 neutralization titers in serum as well (fig. S2H). Last, we observed significantly higher anti-RBD IgG Ab responses in rectal secretions (Fig. 2K), which also positively correlated with live SARS-CoV-2 neutralization titers in serum (fig. 2SI). Overall, group 3 had better Ab responses compared with group 2, suggesting a positive effect of the 3M-052-adjuvant.

### The RBD + 3M-052-alum vaccine induces strong and durable RBD-specific CD8^+^ and T_H_1-biased CD4^+^ T cell responses in the blood

**Fig. 3.**
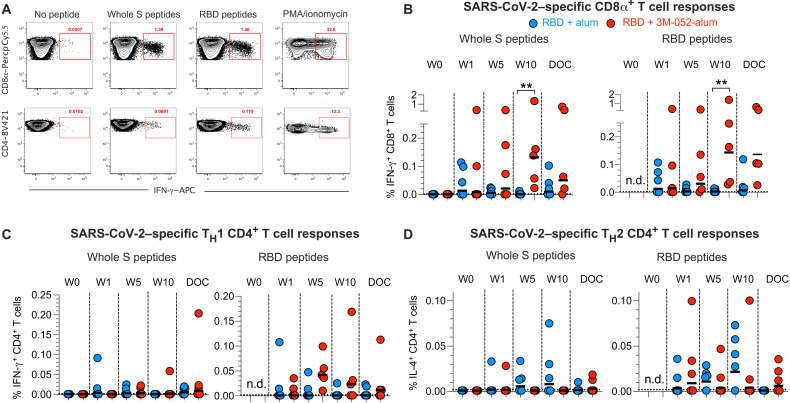
The RBD + 3M-052-alum vaccine in comparison with RBD + alum induces significantly higher RBD-specific CD8^+^ T cells and a T_H_1-biased CD4^+^ T cell response. (**A**) Representative flow cytometry plots show IFN-γ–secreting CD8^+^ and CD4^+^ T cell responses to whole S megapool and RBD-specific peptide pools stimulations ex vivo, as well as no peptide and phorbol 12-myristate 13-acetate (PMA) + ionomycin stimulated negative and positive assay controls, respectively. Scatterplots summarize frequencies of (**B**) IFN-γ^+^CD8^+^ T cells, (**C**) IFN-γ^+^CD4^+^ T cells, and (**D**) IL-4^+^CD4^+^ T cells when stimulating with whole S megapool and RBD peptide pools. n.d., not determined. Horizontal bars represent the geometric mean. A two-tailed Mann-Whitney test was used to compare the significance of differences between groups 2 and 3. **P = 0.0079 in (B).

We next quantified vaccine-specific CD4^+^ and CD8^+^ T cell responses in our study (fig. S4A). We observed induction of RBD-specific IFN-γ^+^CD8^+^ T cell responses as early as week 1 after the primary vaccination in a few animals (Fig. 3, A and B). These responses peaked at week 10 with frequencies significantly higher in group 3 animals in comparison with those in group 2 and persisted on the day of challenge (DOC). A T_H_1-biased, RBD-specific IFN-γ^+^CD4^+^ T cell response was also observed only in group 3 animals peaking at week 5 (Fig. 3C). Consistent with differences observed in RBD-specific IgG subclasses (Fig. 2F), we observed that the RBD-specific IL-4^+^CD4^+^ T cell response (Fig. 3D) trended higher in group 2 animals, suggesting a T_H_2-biased response. Secretion of TNF, another key effector cytokine secreted at peak, was similar to levels of interferon-γ (IFN-γ) with both RBD-specific CD8^+^ and CD4^+^ T cell responses (fig. S4, B and C). In contrast, RBD-specific IL-2^+^CD4^+^ T cell responses peaked at the DOC 4 to 6 weeks after final vaccination in group 3 animals (fig. S4C). Low levels of vaccine-specific interleukin-17 (IL-17) production in CD4^+^ and CD8^+^ T cells were observed in select animals (fig. S4, B and C). Overall, our data highlight that a balance in RBD-specific CD4^+^ and CD8^+^ T cell responses in addition to the RBD-specific Ab responses is achieved when using the RBD + 3M-052-alum vaccine.

### The RBD + 3M-052-alum vaccine significantly reduces viral load in LRT and URT after SARS-CoV-2 challenge

**Fig. 4.**
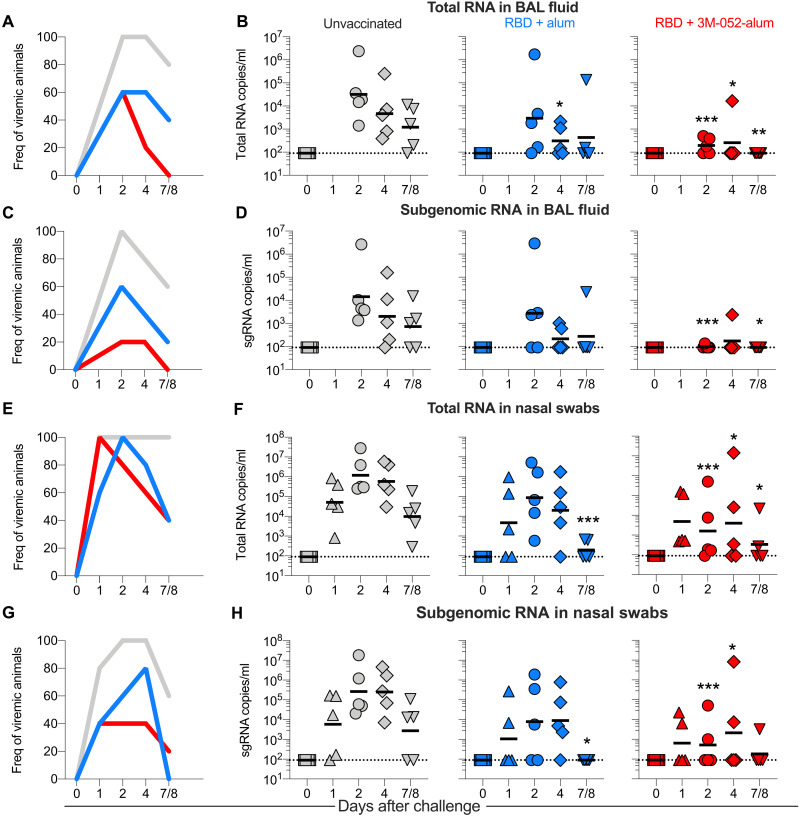
The RBD + 3M-052-alum vaccine significantly reduces total and replicating SARS-CoV-2 in BAL and nasal swabs of RMs post respiratory challenge. (**A**) Line graph indicates frequencies of animals testing positive for total RNA in BAL after challenge. (**B**) Scatterplots indicate total SARS-CoV-2 RNA levels measured in BAL after challenge in all treatment groups. (**C**) Line graph indicates frequencies of animals testing positive for subgenomic (sgRNA) in BAL after challenge. (**D**) Scatterplots indicate sgRNA levels measured in BAL after challenge. (**E**) Line graph indicates frequencies of animals testing positive for total RNA in nasal swabs after challenge. (**F**) Scatterplots indicate total SARS-CoV-2 RNA levels measured in nasal swabs after challenge. (**G**) Line graph indicates frequencies of animals testing positive for sgRNA in nasal swabs after challenge. (**H**) Scatterplots indicate sgRNA levels measured in nasal swabs after challenge. Horizontal lines in graphs (B), (D), (F), and (H) represent geometric means. RMAs were performed on data with VLs to compare differences over time between the study groups as detailed in Materials and Methods. *P = 0.029 for group 2 versus group 1 in (B) and ***P < 0.001, *P* = 0.045, and *P* = 0.006 for group 3 versus group 1 at days 2, 4, and 7/8 in (B). ***P ≤ 0.001 and *P ≤ 0.024 at days 2 and 7/8 in (D). In (F), ***P ≤ 0.001 for group 2 versus group 1 at day 7/8, and ***P < 0.001, *P = 0.032, and *P* = 0.017 at days 2, 4, and 7/8 for group 3 versus group 1. In (H), *P = 0.012 for group 2 versus group 1 at day 7/8 and ***P < 0.001 and *P = 0.041 at days 2 and 4 for group 3 versus group 1. For overall VL AUC measurements (see Materials and Methods), *P* = 0.003 for group 3 versus group 1 and *P* = 0.175 for group 2 versus group 1 in (B). *P* = 0.012 for group 3 versus group 1 and *P* = 0.282 for group 2 versus group 1 in (D). In (F), *P* = 0.01 for group 3 versus group 1 and *P* = 0.006 for group 2 versus group 1. Last, *P* = 0.014 for group 3 versus group 1 and *P* = 0.018 for group 2 versus group 1 in (H).

RMs were challenged approximately a month after the final immunization via the IN and IT routes with a total dose of ~2.3 × 10^5^ plaque-forming units (PFU) of live SARS-CoV-2, WA-1 isolate. Clinical parameters such as change in body weight, blood oxygen saturation, and temperature on the day of and after challenge until termination were closely monitored as detailed in table S1. Moderate but no significant changes in these parameters were recorded in comparison with baseline including with clinical scores (cage-side assessment or physical examination under anesthesia), in any of the groups. No substantial change in comparison with baseline was recorded with serum chemistries after challenge as detailed in table S2. Both a linear mixed effect statistical model appropriate for repeated measures analyses (RMAs) and the area under the curve (AUC) measurements were used for statistical inference of viral load (VL) changes over in nasal swabs, BAL, and throat swabs for both total viral RNA and subgenomic RNA (sgRNA) as detailed in Materials and Methods. Consistent with our recent report (*30*), all unvaccinated animals were infected with SARS-CoV-2, assessed by total RNA detected in BAL fluid at day 2 after challenge (Fig. 4, A and B). In contrast, animals vaccinated with RBD + 3M-052-alum were protected from SARS-CoV-2 infection by day 2, with four of five and all five of five animals having undetectable virus at day 4 and necropsy, respectively. Animals vaccinated with RBD + alum in comparison with unvaccinated controls had lower VLs on day 4. In addition, when comparing the overall mean differences between the area under the VL curves (AUC), a significant difference was observed when comparing group 3 versus group 1 and not when comparing group 2 versus group 1. When sgRNA was measured, VL was detected in 100% of unvaccinated animals but in only 60 and 20% of group 2 and group 3 vaccinated animals, respectively (Fig. 4C). Only one of five animals in group 3 had a detectable VL at day 2 (Fig. 4D), once again significantly lower in comparison with control animals highlighting nearly complete protection at peak with group 3 animals. Four of five and all five of five animals in group 3 had undetectable sgRNA at day 4 and necropsy, respectively. Similar to total RNA with overall AUC measurements, a significant difference again was only observed between groups 3 and 1 and not when comparing groups 2 and 1. Furthermore, we observed reductions in VL in throat swabs (fig. S5) both with total and sgRNA in group 3 (fig. S5, A to D) where 60, 80, and 100% of animals had undetectable total and sgRNA at days 2, 4, and 7/8, respectively. Total RNA VLs were only significantly different in group 3 versus unvaccinated animals, and no significant differences were observed when comparing group 2 versus group 1. We observed significant differences when comparing AUCs with both total and sgRNAs when comparing group 3 versus group 1 and not group 2. In summary, we observed a significant impact of vaccinating with RBD + 3M-052-alum in reducing SARS-CoV-2 viral burden in the LRT.

Persistent total VL in nasal swabs was observed in 100% of control animals from days 1 through necropsy in contrast with rapid control of nasal VL observed by day 2 in many vaccinated animals (Fig. 4, E and F). Reduction in VL was again most apparent when immunizing with RBD + 3M-052-alum in comparison with unvaccinated controls. Identical results were observed when measuring sgRNA in nasal swabs with persistent replicating virus observed in 100% of control animals at days 2 and 4 in contrast to vaccinated animals, most notably in group 3 where significant reduction of VL was observed at days 2 and 4 (Fig. 4, G and H). In contrast with observations in LRT, VLs with both vaccinated groups in comparison with unvaccinated animals were significantly different when measured as AUC and sgRNA, highlighting an impact of vaccination on reducing viral burden in the URT. Overall, these data support the use of RBD + 3M-052-alum vaccine to protect against SARS-CoV-2 infection.

### The RBD + 3M-052-alum vaccine reduces lung pathology after respiratory challenge with SARS-CoV-2

We next evaluated the impact of our vaccine in minimizing lung pathology after challenge. Lung pathology was determined using hematoxylin and eosin (H&E) staining after animal euthanasia (fig. S6, A to C). Total pathology score was calculated considering severity and number of affected lobes, whereas average pathology score was calculated measuring the average severity of abnormalities per affected lobe. The pathology score was reduced in lung sections of animals in group 3 versus group 1 (fig. S6D) but not in group 2 versus group 1 or when comparing group 2 versus group 3. In addition, average pathology score, perivascular cuffing, inflammatory infiltrates, alveolar septal thickening, and type 2 pneumocyte hyperplasia were lower in lung sections of group 3 animals when comparing with those in group 1 (fig. S6, E to I). Thus, group 3 RMs had reduced lung pathology compared with groups 2 and 1.

### Immune correlates of protection upon SARS-CoV-2 challenge in RMs

**Fig. 5.**
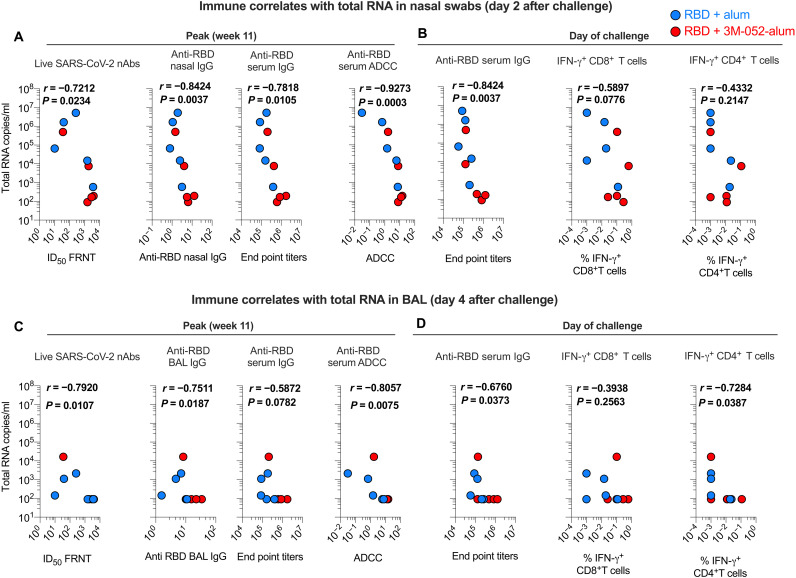
Anti-RBD Ab responses correlate with total viral RNA in URT and LRT. Correlations of anti-RBD Ab and T cell responses with VL in URT and LRT were evaluated at both peak and the DOC. (**A** and **B**) Correlations between Ab and T cell responses induced by vaccines in the study with total SARS-CoV-2 viral RNA in nasal swabs. (**C** and **D**) Correlations between Ab and T cell responses induced by vaccination with total SARS-CoV-2 viral RNA in BAL. Spearman’s correlation was used to identify significance. *P* and *r* values of the Spearman’s correlation test are indicated.

Ab responses and CD8^+^ T cells have been reported to contribute to protecting against SARS-CoV-2 infection (*42*). Here, we correlated Ab and T cell responses to reduction in VL in both URT and LRT. SARS-CoV-2 nAbs, ADCC activity, and anti-RBD binding responses in both serum and nasal secretions at week 11 in the study negatively correlated with levels of total or sgRNA in nasal swabs at day 2 after challenge (Fig. 5A and fig. S7A). A negative correlation was found between anti-RBD Ab responses on the DOC with both total and sgRNA (Fig. 5B and fig. S7B) consistent with the peak Ab responses. We observed no significant association between anti-RBD IFN-γ^+^CD8^+^ T or CD4^+^ T cell responses with total or sgRNA in nasal swabs (Fig. 5B). A negative correlation of nAb and binding Ab responses was also observed with total RNA but not sgRNA in BAL, while ADCC activity positively correlated with both total and sgRNA (Fig. 5, C and D, and fig. S7, C and D). Anti–RBD-IFN-γ^+^CD8^+^ T responses did not correlate with total RNA in BAL, and a correlation was found between RBD-specific CD4^+^ T cell response with total RNA in BAL on DOC (Fig. 5D). Overall, these data highlighted the importance of the induction of persistent anti-RBD serum and mucosal Ab responses in promoting protection against SARS-CoV-2.

### The RBD + 3M-052-alum vaccine reduces expansion of intermediate blood monocytes after SARS-CoV-2 challenge

**Fig. 6.**
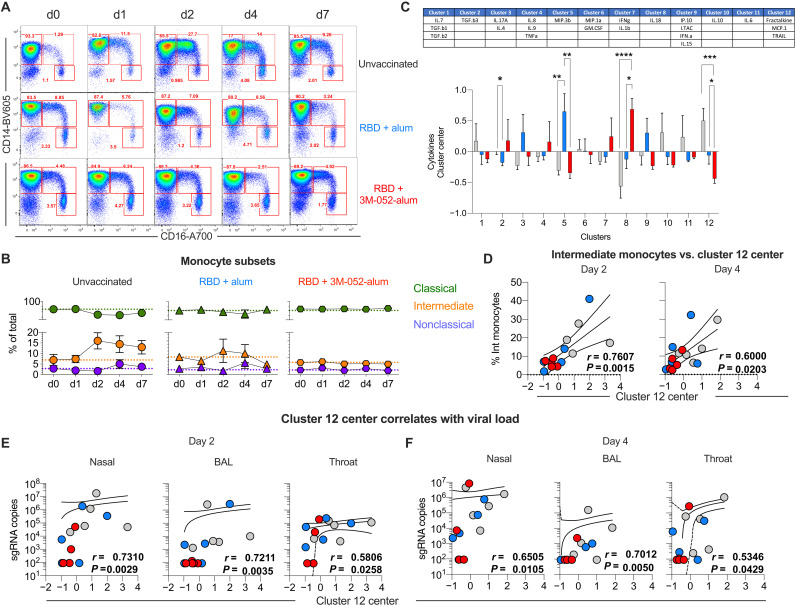
Intermediate (CD14^+^CD16^++^) monocytes in blood and a cluster of chemokines strongly correlate with VL upon SARS-CoV-2 challenge in RMs. We have highlighted a gating strategy to identify monocyte subsets in fig. S7. (**A**) Representative flow plots show changes in classical, intermediate, and nonclassical monocytes at all days after respiratory SARS-CoV-2 challenge in unvaccinated and vaccinated RMs. d, day. (**B**) Line graphs summarize proportional changes in monocyte subsets in all *n* = 5 animals per treatment group. Mean and SEM are reported for all time points sampled. (**C**) Plasma soluble factor components in each cluster (upper table); the *x* and *y* axes represent clusters and contributions to each treatment group. Bars are presented in light gray for unvaccinated (controls), in blue for RBD + alum, and in red for RBD + alum + 3M-052. Error bars indicate SEM. Asterisks *, **, ***, and **** indicate *P* value = 0.04 for cluster 2, *P* = 0.004 for group 2 versus group 1 and 0.0034 for group 3 versus group 2 for cluster 5, *P* < 0.0001 for group 3 versus group 1 and 0.01 for group 3 versus group 2 for cluster 8, and *P* = 0.003 group 3 versus group 1 and 0.049 for group 3 versus group 2 with cluster 12. (**D**) Correlation of the cluster 12 center with the frequencies of IMs at days 2 and 4 (peak). (**E**) Correlation of the cluster 12 center with sgRNA in nasal and throat swabs as well as BAL at day 2 and (**F**) at day 4. Spearman’s correlation was used to identify significance. *P* and *r* values of the Spearman’s correlation test are indicated.

Cytokine storm and infiltration of highly activated innate cells (neutrophils, monocytes, and macrophages) contribute to lung pathology during SARS-CoV-2 infection in humans and RMs (*30*, *43*). Here, we evaluated changes in the frequencies of peripheral blood mononuclear cells (PBMCs) after SARS-CoV-2 challenge. An increase in frequencies of monocytes was most consistently observed in unvaccinated group 1 animals as assessed by complete blood counts (table S3). In addition, we used multiparameter flow cytometry and identified subsets of blood monocytes as previously described (fig. S8) (*23*). Frequencies of intermediate monocytes increased by day 2 after challenge most prominently in unvaccinated animals and persisted at higher levels until termination (Fig. 6, A and B). In contrast, frequencies of intermediate monocytes were unchanged in vaccinated animals, suggesting an impact of vaccine-induced immunity in blocking such an expansion. Also, there was a transient but notable increase in the frequency of blood plasmacytoid dendritic cells at day 1 after challenge once again only in groups 1 and 2 with no significant changes in frequencies of myeloid DCs, lymphocytes, and NK cells in any groups (figs. S9 and S10).

We next measured 27 analytes in plasma of all animals after challenge (days 0 to 7/8) using a multiplexed MesoScaleDiscovery (MSD) platform. We first performed a principal components analysis (PC1: 16.9%, PC2: 16.1%) to evaluate the overall segregation of analytes in all animals upon challenge (fig. S11A). The contribution of analytes (cytokines/chemokines/interleukins and IFNs) to each principal component is highlighted in fig. S11B. We performed a multivariate analysis using gap statistics and k-means clustering to identify the most prominent clusters in the entire dataset (fig. S11C). A total of 12 clusters were identified as tabulated in Fig. 6C. Cluster 12, a combination of fractalkine (chemokine ligand 1), monocyte chemotactic protein (MCP-1), and tumor necrosis factor (TNF)–related apoptosis-inducing ligand or TRAIL, was significantly up-regulated in group 1 animals, whereas a significant reduction was observed in group 3 animals compared with groups 1 and 2. Cluster 8 with IL-18 and cluster 5 composed of MIP.3b were selectively up-regulated in group 3 and group 2 animals, respectively, highlighting distinct outcomes of vaccination in response to SARS-CoV-2 infection in RMs (Fig. 6C). A positive correlation was observed between cluster 12 center versus frequencies of intermediate monocytes at days 2 and 4 (Fig. 6D), a time point at which a peak VL was observed in LRT and URT (Fig. 4 and fig. S4). Baseline or DOC cluster 12 center did not correlate with viral sgRNA in nasal or throat swabs at both days 2 and 4, whereas a modest association was observed with sgRNA in BAL at day 2 and not with day 4 (fig. S11, D and E). In contrast, cluster 12 center values positively correlated with the magnitude of sgRNA in nasal, BAL, and throat at days 2 and 4, highlighting a potential plasma biomarker of SARS-CoV-2 virus levels in LRT and URT (Fig. 6, E and F). Frequencies of intermediate monocyte also positively correlated with sgRNA in LRT and URT only at day 2 (fig. S11F) and not at day 4 (fig. S11G). A negative correlation of cluster 12 with classical monocytes was observed (fig. S12A). No correlation of cluster 12 with DC subsets or B cells was observed with viral sgRNA levels (Fig. S12, B to F). Last, both frequencies of intermediate monocytes and cluster 12 at day 2 and not day 4 after challenge positively correlated with lung pathology (fig. S13, A and B); however, VLs after challenge in the respiratory mucosa did not correlate with lung pathology, with the exception of BAL VL at day 2 (fig. S13, C and D). In summary, we report a targeted and early impact of SARS-CoV-2 respiratory infection on blood monocytes, which was strongly suppressed in animals vaccinated with RBD + 3M-052-alum. We also report the identification of potential plasma biomarkers found in cluster 12 (fractalkine, MCP-1, and TRAIL), which correlated with respiratory levels of SARS-CoV-2.

### RBD + 3M-052-alum vaccine–induced plasma cells accumulate in draining lymph nodes and not in the BM

**Fig. 7.**
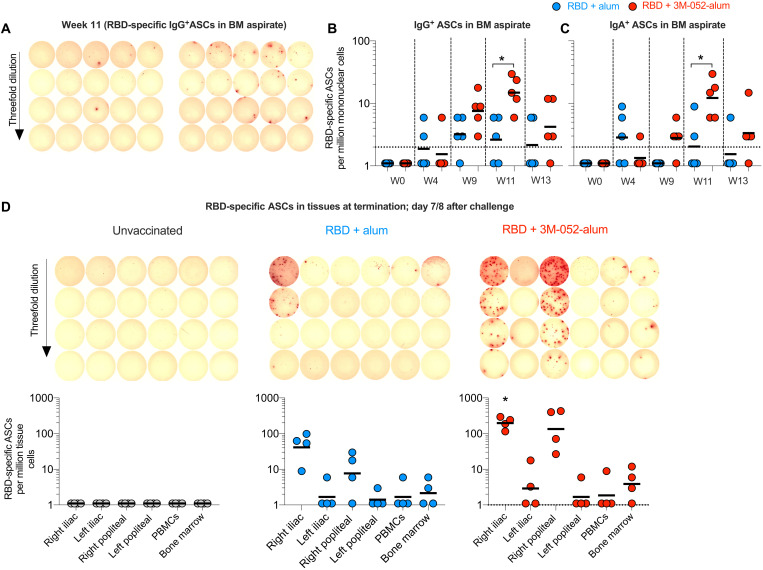
The RBD + 3M-052-alum vaccine in comparison with RBD + alum induces significantly higher RBD-specific ASCs in draining LNs. RBD-specific ASCs were enumerated using an ELISpot assay. (**A**) Scanned ELISpot plate images of RBD-specific ASCs at week 11 assayed in BM aspirate are shown. (**B**) Scatterplots summarize frequencies of IgG-secreting RBD-specific ASCs in BM aspirates collected before and after vaccination up to a week before challenge. (**C**) Scatterplots summarize frequencies of IgA-secreting RBD-specific ASCs in BM aspirates. (**D**) Scanned ELISPOT plate images of RBD-specific ASCs in draining and nondraining iliac and popliteal LNs, PBMCs, and BM long bone (femur) scoop tissue at necropsy after challenge are shown for one representative animal in each treatment group. Scatterplots below the scanned images are aligned to indicate the tissue in each column of the ELISpot plate. Data reported here summarize the frequencies of IgG-secreting ASCs in LNs, PBMCs, and BM of *n* = 4 animals per treatment group. The significance of the difference in the frequencies of RBD-specific ASCs in tissues was established using a two-tailed Mann-Whitney test. *P = 0.023 in (B), *P* = 0.032 in (C), and *P* = 0.0238 in (D) when comparing groups 3 and 2.

Vaccination of RMs with an HIV-1, envelope (Env) based vaccine using the alum-3M-052 adjuvant compared with alum alone induced high frequencies of Env–specific Ab-secreting cells (ASCs) in blood as well as successful homing and persistence of LLPCs in both the BM and lymph nodes (LNs) (*23*). Here, we quantified frequencies of RBD-specific ASCs first in peripheral blood at day 4 after the second and third vaccinations, respectively, a time point previously established to be a peak for the presence of ASCs specifically in RMs (*23*, *38*). Higher frequencies of RBD-specific IgG^+^ and IgA^+^ ASCs were observed in group 3 animals relative to group 2, both after the second and third immunizations (fig. S14, A to C). We next quantified the frequencies of RBD-specific ASCs in BM aspirates. While RBD-specific ASCs were detectable after the second vaccination at week 9, significantly higher frequencies of RBD-specific ASCs in the BM were observed at week 11 when vaccinating with RBD + 3M-052-alum compared with group 2 (Fig. 7, A to C). In contrast to the persistence observed with LLPCs induced by HIV-1 env immunogens in BM (*23*), we saw a sharp reduction in the frequencies of RBD-specific BM ASCs in aspirate samples between week 11 (peak) and week 13 (prechallenge; Fig. 7B). Euthanizing animals a week after SARS-CoV-2 challenge allowed us to investigate the distribution of ASCs in vaccine draining LNs and in the femur BM. Consistent with our previous work in mice and NHPs, we observed a notable number of vaccine/RBD-specific IgG^+^ ASCs in draining iliac and popliteal LNs (Fig. 7D), whereas no responses were observed in the contralateral LNs (*23*, *44*). These responses were significantly higher in group 3 animals versus group 2 (Fig. 7D). However, once again in sharp contrast to our HIV-1 data, we saw very low RBD-specific LLPCs in the femur-derived BM (Fig. 7D). The lack of accumulation of RBD-specific LLPCs in the BM needs to be further investigated in the context of other SARS-CoV-2 vaccines.

### The RBD + 3M-052-alum vaccine induces higher expression of tissue homing chemokine markers on vaccine enriched blood ASCs

**Fig. 8.**
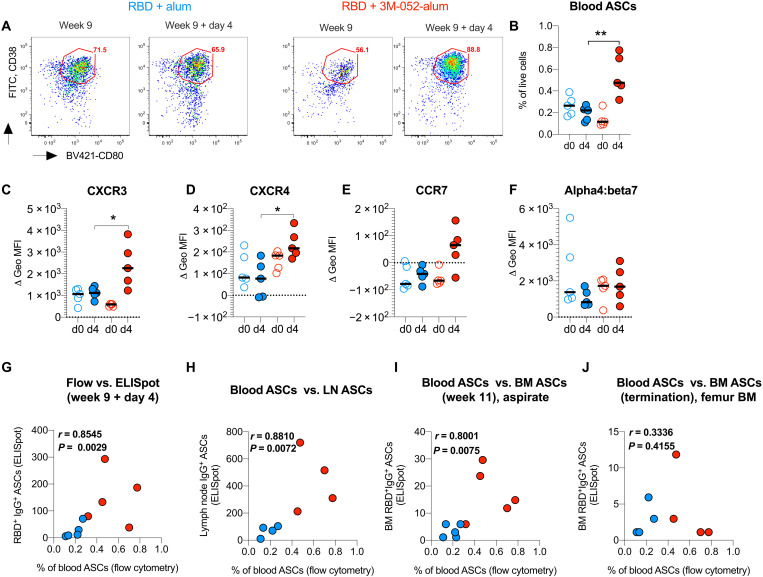
The RBD + 3M-052-alum vaccine in comparison with RBD + alum induces increased frequencies of blood ASCs and up-regulation of tissue homing markers. (**A**) Flow plots highlight CD38^+^CD80^+^ ASCs (gating strategy in fig. S12) from one animal each vaccinated with RBD + alum or RBD + 3M-052-alum adjuvants at week 9 and week 9 + day 4. FITC, fluorescein isothiocyanate. (**B**) Scatterplots summarize frequencies of blood ASCs before and after vaccination at week 9. Scatterplots in (**C**) to (**F**) summarize the change in differential geometric mean fluorescence intensity (MFI) (stain isotype) of chemokine receptors CXCR3, CXCR4, CCR7, and alpha4:beta7 on blood ASCs, before and day 4 after vaccination at week 9 in the study. (**G**) Correlation of flow-based frequencies of total blood ASCs with RBD-specific ELISpot–based IgG^+^ ASCs at week 9 + day 4 in the study is shown. (**H**) Correlation of RBD-specific IgG^+^ blood ASCs (week 9 + day 4) with IgG^+^ ASCs in draining LNs at termination after challenge is shown. (**I**) Correlation of RBD-specific IgG^+^ blood ASCs (week 9 + day 4) with IgG^+^ ASCs in BM aspirates at week 11 is shown. (**J**) Correlation of RBD-specific IgG^+^ blood ASCs (week 9 + day 4) with IgG^+^ ASCs in femur BM scoop tissue at necropsy is shown. Statistical significance of the difference in responses measured was tested using a nonparametric two-tailed Mann-Whitney test. *P* < 0.05 was used as significant. **P = 0.0079 in (B), *P = 0.03 in (C), and *P = 0.0159 in (D).

We next investigated the expression of chemokine markers on blood ASCs before and after the final vaccination using flow cytometry (fig. S15) (*45*) to understand the qualitative impact of adjuvants on vaccine enriched ASCs. ELISpot assays had revealed a significantly higher increase in RBD-specific IgG- and IgA-secreting cells at day 4 after the final vaccination at week 9 (fig. S14). Consistent with these data, here we saw significantly higher frequencies of CD38^+^CD80^+^ (all lineage negative cells) cells when vaccinating with RBD + 3M-052-alum in comparison with RBD + alum (Fig. 8, A and B). In addition, we also observed higher expression of chemokine receptors, CXCR3 (trafficking to inflamed tissue such as LNs) and CXCR4 (trafficking to the BM) (Fig. 8, C and D, and fig. S15) (*46*, *47*), when vaccinating with RBD + 3M-052-alum compared with alum alone. A modest increase in CCR7 expression was observed on ASCs when vaccinating with RBD + 3M-052-alum and no up-regulation of alpha4:beta7 was observed in any group (Fig. 8, E and F). There was a positive correlation between blood ASCs in all vaccinated animals at day 4 with corresponding RBD-specific IgG^+^-secreting cells (Fig. 8G). This highlighted the enrichment of vaccine-specific ASCs. Frequencies of blood ASCs in all vaccinated animals at day 4 also positively correlated with RBD-specific IgG + ASCs in LNs (popliteal + iliac; combined) quantified at euthanasia (Fig. 8H) and with week 11 ASCs in BM aspirates (Fig. 8I) but not with ASCs in long bone femur scoop tissue (Fig. 8J). Overall, these observations further inform on the qualitative impact of alum and 3M-052-alum adjuvants on chemokine receptor expression on vaccine enriched ASCs.

Last, we evaluated anamnestic RBD-specific T and Ab responses after challenge. No appreciable change in frequencies of RBD-specific CD8^+^ and CD4^+^ T cells in comparison with prechallenge was observed in PBMCs at the time of necropsy (fig. S16, A to F). No changes were also seen in the magnitude of anti-RBD binding Ab responses between the DOC and necropsy (fig. S16, G to I). RBD-specific CD8^+^ and CD4^+^ T cell responses were also assayed in hilar LNs that drain the lung. No SARS-CoV-2–specific CD8^+^ (fig. S17) and CD4^+^ T cell responses (fig. S18) were detected in hilar LNs of naïve control animals in group 1 at necropsy. Significantly higher CD8^+^ T cell and CD4^+^ T cell responses were observed only in group 3 animals, suggesting that these responses were likely induced by vaccination and not by the SARS-CoV-2 challenge. Overall, these data inform on the tissue distribution of vaccine-induced B and T cell responses against SARS-CoV-2.

## DISCUSSION

Many SARS-CoV-2 vaccine candidates are based on the whole S protein and limited testing in NHPs has been reported with RBD-based vaccines (*7*, *35*, *36*, *48*, *49*). Because RBD-targeted binding Abs correlate very strongly with virus-neutralizing activity in natural infections and vaccinations (*18*), an RBD immunogen offers a target for rational vaccine design both immunologically and from a manufacturability point of view (*8*). Our study highlights induction of robust anti-SARS-CoV-2 Ab responses and protection from SARS-CoV-2 infection and pathogenesis specifically when using the RBD + 3M-052-alum vaccine formulation.

Consistent with previous reports of live SARS-CoV-2 nAbs induced by chimpanzee adenovirus (ChAdOx-1) (*33*) or with the adenovirus 26–based (Ad.26) viral vectored vaccines in NHPs (*35*), nAbs were observed after two vaccinations in our study. However, these titers were inferior when compared with whole S immunogen–based mRNA vaccine after two vaccinations (*36*). One explanation for this outcome is the presence of additional neutralizing and nonneutralizing epitopes contributing to overall higher titers when using a whole S immunogen versus RBD. Clinical subunit vaccines and preclinical studies in NHPs have documented significant increases in magnitude (peak) and quality of Ab responses against protein vaccines after three vaccinations (*23*, *38*). We report higher live SARS-CoV-2 nAbs and pseudovirus nAb in group 3 animals after the third vaccination with a relatively lower increase in binding Abs. In addition, a smaller drop observed in cross-neutralizing activity against the Beta strain selectively in animals vaccinated with RBD-3M-052-alum suggests potential maturation of Ab responses. We have recently reported strong germinal center (GC) responses with repeated vaccinations when using 3M-052 and HIV-1 Env immunogens (*23*), which could have contributed to improve nAbs after the third vaccination. Such induction of GCs with RBD vaccination needs to be investigated in future studies. While a role for nonneutralizing Abs in protection with vaccine-induced immunity is unclear, treatment with monoclonal Abs (mAbs) in therapeutic settings highlights benefits with Fc effector functions in reducing lung pathology (*50*). On the basis of these reports, we hypothesize that anti-RBD Abs were skewed by adjuvants toward IgG1 (higher effector function in macaques in comparison with IgG3) or IgG4 subclasses (*41*), followed by strong ADCC activity that correlates with VLs in mucosa as observed in group 3 or 2, respectively, and may differentially contribute to protection.

A protective SARS-CoV-2 vaccine should ideally induce a protective mucosal response. We observed strong anti-RBD Ab responses in nasal, BAL, and rectal secretions when vaccinating via the intramuscular (IM) route, which may contribute substantially to minimizing transmission and onset of disease followed by a reduction in fecal shedding of virus, respectively. It is unknown whether an IN route of vaccination will further improve mucosal immunity with local IgA Abs. Most SARS-CoV-2 vaccines approved under emergency use authorization follow a two-dose regimen. Because we observed protective efficacy after three vaccinations, additional investigation including testing in humans with two vaccine doses is critical to assess protective efficacy. Robust induction of cross-CoV neutralizing Ab responses in RMs when vaccinating with a multimeric RBD displaying nanoparticle immunogen with the 3M-052-alum has recently been reported (*51*). These data are consistent with strong immunogenicity of multimeric nanoparticle protein-based construct used in the Novavax vaccine adjuvanted with Matrix-M (*52*). Collectively, we conclude that 3M-052-alum may offer significant promise in improving immunogenicity of both monomeric and multimeric RBD-based SARS-CoV-2 vaccines.

Robust CD8^+^ T cell responses, nAbs, and T_H_1-biased CD4^+^ T cells could also support protective immunity against SARS-CoV-2 (*53*). Specifically, where nAb responses can be suboptimal against emerging VOCs (*27*), conserved epitope-based CD8^+^ T cell responses could support protection. No evidence of induction of robust CD8^+^ T cell responses was observed when using the recently approved mRNA-1273 vaccine from Moderna, inactivated SARS-CoV-2 from Sinovac, or a recombinant protein from Novavax in NHPs (*36*, *48*, *52*). The saponin-based adjuvant used in the Novavax vaccine induces CD8^+^ T cell responses with protein immunogens in mice (*54*). To date, SARS-CoV-2–specific CD8^+^ T cells have only been induced by viral vectored ChAd0X-1 and Ad.26 and/or Ad5 in NHPs or with the BNT162b2, an mRNA vaccine in humans (*7*). Induction of anti-RBD CD8^+^ T cell responses in our current study is in contrast to our inability to consistently induce either anti-simian immunodeficiency (SIV) gag or anti-Env CD8^+^ T cell responses in RMs with HIV-1 or SIV immunogens when using TLR-7/8 agonist–based adjuvants (*23*, *38*). It is possible that the RBD immunogen is enriched in epitopes for CD8^+^ T cells or preferentially targets cross-presenting DCs. However, as discussed above, RBD-containing whole S protein–based vaccines expressed in mammalian cells do not induce appreciable CD8^+^ T cell responses. Alternatively, unique glycosylation patterns of a yeast-expressed RBD immunogen (*20*) that could improve targeting of lectin receptors on DC subsets (*55*) including monocyte-derived inflammatory DCs (*56*), may better induce CD8^+^ T cells. This hypothesis is well supported by (i) reported addition of distinct numbers of mannose residues when expressing proteins in mammalian (~8 mannose residues) versus yeast hosts (up to 20 mannose residues) (*57*) and (ii) the fact that mannose receptor mediates uptake of soluble mannose–expressing antigens for cross-presentation (*58*). In summary, yeast expression of RBD in combination with a T_H_1 biasing 3M-052 adjuvant likely led to targeting and differentiation of inflammatory DCs, supporting the induction of CD8^+^ T cells that may improve vaccine efficacy against emerging SARS-CoV-2 variants.

Differentiation of classical to intermediate monocytes has been documented in dengue virus infections in humans (*59*), as well as when using TLR-7/8 adjuvants in RMs (*23*, *38*). Intermediate monocytes favor B cell differentiation to ASCs via secretion of IL-10 and B cell–activating factor (*59*). SARS-CoV-2 is a single-stranded RNA virus with potential to target TLR-7/8 receptors. Our data support the idea that respiratory infection with SARS-CoV-2 leads to activation of blood monocytes. Precise immunological mechanisms by which either anti-RBD Ab or T cell responses induced by RBD + 3M-052-alum vaccine in respiratory mucosa contribute to blocking activation of peripheral blood monocytes are unclear. We hypothesize that anti-RBD Ab responses in nasal and BAL mucosa could be contributing to rapid clearance of virus via formation of immune complexes and perhaps signaling via Fc inhibitory receptors on innate immune cells as seen with mAbs in therapeutic settings (*50*). A larger role for monocytes/macrophages in BAL after SARS-CoV-2 challenge has been reported in both humans and RMs (*30*, *43*). Identification of cluster 12 comprising monocyte-related analytes (fractalkine, MCP-1, and TRAIL) in plasma further supports a targeted impact of SARS-CoV-2 infection on monocyte recruitment to lung respiratory mucosa contributing to exaggerated pathogenicity of COVID-19.

Currently, there is limited knowledge on the ability of SARS-CoV-2 vaccines to induce BM homing LLPCs in NHPs or humans, which is predictive of the longevity of Ab responses. Here, we document a transient presence of RBD-specific ASCs in RM BM aspirate after the third vaccination that contracts rapidly between weeks 11 and 13 in contrast with our previous work with adjuvanted HIV-1 immunogen–specific BM LLPCs (*23*). We also previously documented that repeated BM sampling can deplete finite numbers of plasma cells in aspirates (*23*) and, hence, additionally verified the presence of RBD-specific LLPCs in the femur BM at necropsy. In contrast with our studies using HIV-1 immunogens, we observed low levels of RBD-specific LLPCs in the femur BM scoop tissue. However, we documented robust frequencies of RBD-specific ASCs in both iliac and popliteal LNs draining the vaccination site consistent with our previous observations in both mice and RMs (*23*, *44*). While quantifying the relative contributions of LN- versus BM-resident LLPCs in supporting Ab responses is challenging, it is reasonable to hypothesize that draining LNs may provide an alternate site of persistence of SARS-CoV-2 vaccine–specific LLPCs that could support durability of RBD-specific Ab responses. Increased expression of CXCR3 and higher CCR7 on blood ASCs when vaccinating with RBD + 3M-052-alum may support trafficking to inflamed draining LNs. Alternatively, a significant proportion of LN ASCs could have also failed to egress into the periphery. While increased CXCR4 expression on blood ASCs may in part explain transient homing of blood ASCs to the BM, a sharp contraction in frequencies of RBD-specific ASCs in the BM suggests a short life span or poor quality of these cells. These findings highlight the need for longitudinal tracking of SARS-CoV-2 plasma cells in parallel with serum Ab responses.

We face continued challenges with supply, distribution under extreme cold-chain requirements, and availability of SARS-CoV-2 vaccines in low-income or developing countries. We reasoned that a vaccine design based on a low-cost production process matching existing platforms such as with the manufacture of hepatitis B vaccines could be easily repurposed for local production and meeting global requirements of a SARS-CoV-2 vaccine. Our study supports the testing of an RBD-based immunogen adjuvanted with 3M-052-alum in human trials, which could potentially be a cost-effective, scalable, and thermostable SARS-CoV-2 vaccine.

## MATERIALS AND METHODS

### Study design

The objective of our study was to establish the immunogenicity and protective efficacy of a SARS-CoV-2–derived yeast-expressed RBD monomer immunogen. We formulated RBD with two clinically relevant adjuvants in alum and 3M-052-alum. Using the RM model (*n* = 5 per treatment group), we tested the immunogenicity and protective efficacy of this vaccine upon a respiratory challenge with live SARS-CoV-2 as detailed in Fig. 1. We investigated correlation of vaccine-induced Ab and T cell responses with VLs in the respiratory mucosa upon challenge. In addition, we evaluated changes in RM blood PBMCs and soluble analytes after challenge and correlation of such blood parameters with VLs in the respiratory mucosa. Last, we investigated the distribution of vaccine-specific plasma cells in blood, LNs, and BM.

### Cloning and expression of SARS-CoV-2 RBD in the yeast *P. pastoris*

As recently described (*28*), RBD219-WT in *P. pastoris* X33 was produced by fermentation at the 5-liter scale and the target protein was purified through a combination of anion exchange, hydrophobic interaction, and SE chromatography. Additional details are provided in the Supplementary Materials.

### Adjuvants and formulations

3M-052, a TLR-7/8 agonist, was provided by 3M Corporate Research and Materials Laboratory (MN, USA). Aluminum oxyhydroxide (Alhydrogel “85”) was procured from Brenntag Biosector, Denmark, and formulated and aliquoted for use at The Infectious Disease Research Institute (IDRI). 3M-052 adsorption to alum was facilitated by first preparing a lipid nanosuspension with 3M-052 and distearoyl phosphatidylglycerol (DSPG) at a 1:4 3M-052:DSPG molar ratio, which was then mixed with aluminum oxyhydroxide following a procedure adapted from our previous report (*60*).

### Animals

Fifteen RMs, aged ~5 to 7 years (all male), were identified for the study from the Yerkes National Primate Research Center (YNPRC) colony. YNPRC’s animal care facilities are accredited by both the U.S. Department of Agriculture (USDA) and the Association for Assessment and Accreditation of Laboratory Animal Care. All animal procedures were performed in accordance with guidelines established by the Emory University Institutional Animal Care and Use Committee Guidelines and those set up by the NIH’s *Guide for the Care and Use of Laboratory Animals*, *8th Edition*.

### Immunization details

Animals were immunized three times in the right calf muscle via the IM route of vaccination at weeks 0, 4, and 9 as detailed in the study timeline in Fig. 1. RBD-WT was used at 100 μg, alum was used at 500 μg, and 3M-052 was used at 10 μg per dose. RBD immunogen stored at −80°C was thawed and mixed with alum or 3M-052-alum adjuvant formulations for 20 min at room temperature (R.T.) on an end-to-end shaker to allow adsorption. Saline was used as the diluent to make up volumes, and all animals were immunized with 1.0 ml of the final inoculum. All immunizations and samplings were performed under sedation with ketamine and/or Telazol.

### Analyses of anti-RBD IgG binding Ab responses

RBD-specific binding Abs were assayed using a recently described assay with minimal modifications (*61*). Briefly, RBD-coated enzyme-linked immunosorbent assay (ELISA) plates were incubated with serially diluted sera from prevaccination and vaccinated time points followed by detection using horseradish peroxidase–conjugated anti-monkey Ab (SB108A, Southern Biotech, Al, USA) and absorbance analyses after incubation with tetramethyl benzidine (TMB) substrate. Additional details are included in the Supplementary Materials.

### Multiplexed analyses of anti-RBD, spike, and nucleocapsid Ab responses

Serum IgG binding to SARS-CoV-2 spike, RBD, and nucleocapsid proteins was evaluated using the 4-PLEX SARS-CoV-2 Panel 2 Kit from MSD. Briefly, samples were incubated at multiple dilutions on the plates preprinted with SARS-CoV-2 antigens, followed by detection with SULFO-Tag–labeled anti-human IgG Ab. Electrochemiluminescence (ECL) signal from each sample against each antigen was quantified by applying electricity to electrodes built in assay plates. The magnitude of binding, evaluated as arbitrary units (AU/ml), was calculated by backfitting the binding ECL on the four-parameter logistic regression model, which was generated by titration of the kit’s reference standard with assigned AU concentration.

### Pseudovirus neutralizing assay

SARS-CoV-2 neutralization was assessed with spike-pseudotyped virus in 293T/ACE2 cells as a function of reductions in luciferase (Luc) reporter activity. 293T/ACE2 cells were provided by M. Farzan and H. Mu at Scripps Research Institute. Cells were maintained in Dulbecco’s modified Eagle’s medium (DMEM) containing 10% fetal bovine serum, 25 mM Hepes, gentamycin (50 μg/ml), and puromycin (3 μg/ml). An expression plasmid encoding codon-optimized full-length spike of the Wuhan-1 strain (VRC7480) was provided by B. Graham and K. Corbett at the Vaccine Research Center, National Institutes of Health (USA). The D614G amino acid change was introduced into VRC7480 by site-directed mutagenesis using the QuikChange Lightning Site-Directed Mutagenesis Kit from Agilent Technologies (catalog no. 210518). The mutation was confirmed by full-length spike gene sequencing. Pseudovirions were produced in HEK 293T/17 cells (ATCC, catalog no. CRL-11268) by transfection using FuGENE 6 (Promega, catalog no. E2692) and a combination of spike plasmid, lentiviral backbone plasmid (pCMV ΔR8.2), and firefly Luc reporter gene plasmid (pHR′ CMV Luc) in a 1:17:17 ratio. Transfections were allowed to proceed for 16 to 20 hours at 37°C. Media were removed, monolayers were rinsed with growth medium, and 15 ml of fresh growth medium was added. Pseudovirus-containing culture medium was collected after an additional 2 days of incubation and was clarified of cells by low-speed centrifugation and 0.45-μm filtration and stored in aliquots at −80°C. TCID_50_ (median tissure culture infectious dose) assays were performed on thawed aliquots to determine the infectious dose for neutralization assays [relative luminescence units (RLU) 500× to 1000× background, background usually averages 50 to 100 RLU].

For neutralization, a pretitrated dose of virus was incubated with eight serial fivefold dilutions of serum samples in duplicate in a total volume of 150 μl for 1 hour at 37°C in 96-well flat-bottom poly-l-lysine–coated culture plates (Corning Biocoat). Cells were suspended using TrypLE Select Enzyme solution (Thermo Fisher Scientific) and immediately added to all wells (10,000 cells in 100 μl of growth medium per well). One set of eight control wells received cells + virus (virus control) and another set of eight wells received cells only (background control). After 66 to 72 hours of incubation, the medium was removed by gentle aspiration and 30 μl of Promega 1× lysis buffer was added to all wells. After a 10-min incubation at R.T., 100 μl of Bright-Glo Luc reagent was added to all wells. After 1 to 2 min, 110 μl of the cell lysate was transferred to a black/white plate (PerkinElmer). Luminescence was measured using a PerkinElmer Life Sciences, Model Victor2 luminometer. Neutralization titers are the serum dilution at which RLU were reduced by either 50% inhibitory dose (ID_50_) or 80% (ID_80_) compared with virus control wells after subtraction of background RLUs. Serum samples were heat-inactivated for 30 min at 56°C before assay.

### Live SARS-CoV-2 neutralizing or focus reduction neutralization titer assay

Neutralization assays with authentic SARS-CoV-2 virus were performed as previously described (*62*). Briefly, serially diluted sera from vaccinated animals incubated (1 hour) with 100 to 200 focus forming unit (FFU) infectious clone–derived SARS-CoV-2-mNG (*63*) were added to VeroE6 cell monolayer for an hour. After the removal of inoculum and incubation with 0.85% methylcellulose containing DMEM for 24 hours, cells were fixed with 2% paraformaldehyde (PFA) for 30 min and washed, and foci were visualized on a fluorescence ELISpot plate reader (*64*). Additional details on the assay and calculation of nAb titers are detailed in the Supplementary Materials.

### ELISA for anti-RBD IgG subclasses

Immulon 4 plates were coated overnight with RBD, except for two rows dedicated for standard. These wells were coated with either purified IgG1, IgG2, IgG3, or IgG4 (all from the NHP Reagent Resource). Plates loaded with serum samples were similarly reacted overnight. Plates were treated with the following respective mAbs (1 μg/ml) to rhesus IgG1, IgG2, IgG3, and IgG4 (all from the NHP Reagent Resource): 3C10.3, 3C10.1, 2G11, and 7A8. After 1 hour at 37°C, the rhesus subclass–specific mAb was detected with either SBA #1082-08 biotinylated goat anti-mouse IgG2a (for 3C10.3) or SBA #1071-08 biotinylated goat anti-mouse IgG1 (for 3C10.1, 2G11, and 7A8). Plates were then washed and reacted with 1:2000 diluted neutralite avidin-peroxidase (SBA #7200-05) for 30 min at R.T and then washed and developed with TMB (SBA #0410-01).

### ELISA for anti-RBD IgG in secretions

Immulon 4 microtiter plates (Thermo Fisher Scientific) were coated overnight with 100 ng per well of the RBD immunogen in phosphate-buffered saline (PBS). Plates were then washed with PBS containing 0.05% Tween 20 (PBST) and blocked for 30 min with 0.1% bovine serum albumin in PBST. Standard and samples diluted in fresh block buffer were then added. The IgG standard was an anti-RBD human IgG Ab (ACRO #SAD-S35). After overnight storage at 4°C, plates were washed and treated with biotinylated anti-human g chain Ab (SBA #2048-08) for 1 hour at 37°C. Plates were then washed and reacted with 1/2000 neutralite avidin-peroxidase (SBA #7200-05) for 30 min at R.T. Plates were washed and developed with TMB (SBA #0410-01). Absorbance was recorded at 370 nm after 30 min. Concentrations of Ab were subsequently interpolated from standard curves constructed with SoftMax Pro software (Molecular Devices). For secretions, concentrations of antigen-specific IgG Abs were normalized by dividing by the concentration of total IgG, measured by ELISA.

### ELISA for total IgG in secretions

Immulon 4 plates were coated overnight with 50 ng per well of goat anti-monkey IgG (Rockland). Plates were then washed, blocked, and loaded with serially diluted secretions. The standard was rhesus IgG (Rockland). Plates were developed as described above with anti-RBD IgG in secretions using biotinylated goat anti-human g chain Ab (SBA).

### Ab-dependent phagocytosis

Phagocytosis assays were performed as previously described (*65*). Briefly, 1-μm avidin-coated fluorescent beads (Molecular Probes) were labeled with biotinylated anti-HIS tag Ab followed by the HIS-tagged RBD immunogen. Beads (25 μl) were preincubated at 37°C in V-bottom plates with diluted serum samples (25 μl). After 1 hour, 2 × 10^4^ THP-1 cells (in 50 μl) were added to each well. After 5 hours at 37°C in 5% CO_2_, the cells were washed in Dulbecco’s PBS (lacking Ca^+2^ and Mg^+2^) and then treated with trypsin for 5 min. The cells were washed and resuspended in 1% PFA. Fluorescence was analyzed using a FACS Canto (BD Biosciences), and FlowJo software (BD Biosciences) was used to determine the percentage of bead^+^ cells and multiply them by their median fluorescence intensity. A phagocytic score was calculated for each test sample by dividing this value by the average value obtained for four RBD naïve sera at the same dilution. A score of 2.0 was considered significant.

### Spike protein–expressing cell Ab binding assay

The cell Ab binding assay was performed based on our previously described methods (*66*–*68*) modified to use target cells transfected with plasmids designed to express the SARS-CoV-2 spike protein (S protein) with a C-terminal flag tag. Serum from vaccinated animals was serially diluted and incubated with S-expressing transfected cells and % rhesus IgG^+^ cells were quantified as detailed in the Supplementary Materials.

### Ab-dependent NK cell degranulation assay

Cell surface expression of CD107a was used as a marker for NK cell degranulation, a prerequisite process for ADCC (*69*), performed as previously described (*70*). 293 target T cells transfected with the S protein from the G614 variant were incubated at a 1:1 ratio with NK cells from healthy volunteers in the presence or absence of test sera for 6 hours at 37°C. Cell surface expression of CD107a was used to assess NK cell degranulation or ADCC-like activity as detailed in the Supplementary Materials.

### T cell stimulation and intracellular cytokine staining assays

The assay was performed as described before (*23*). Briefly, ~2 × 10^6^ PBMCs were cultured in 200-μl final volume in 5-ml polypropylene tubes (BD Biosciences, San Diego, CA, USA) in the presence of anti-CD28 (1 μg/ml) and anti-CD49d (1 μg/ml) (BD Biosciences) and the following conditions: (i) negative control with dimethyl sulfoxide only, (ii) whole spike (S) peptide pool 1 (*n* = 253 peptides, 15-mers with 10-residue overlap) (Weiskopf and Sette labs, LJI, La Jolla, CA) at a final concentration of 1 μg/ml, (iii) RBD peptide pool 2 (53 peptide pool, 15-mers with 11-residue overlap, JPT Peptide Technologies, Germany) at a final concentration of 1 μg/ml, (iv) N peptide pool (only prechallenge and postchallenge time points), and (v) phorbol 12-myristate 13-acetate/ionomycin. Brefeldin A was added to all tubes at 10 μg/ml (Sigma-Aldrich, St Louis, MO) and cells were cultured for 6 hours and transferred to 4° before staining for flow cytometry as detailed in the Supplementary Materials.

### PBMC staining for flow cytometry after challenge

PBMCs were stained as described before (*23*) and detailed in the Supplementary Materials.

### MSD cytokine assay

Meso Scale U-PLEX assay (Meso Scale MULTI-ARRAY Technology) commercially available by MSD was used for plasma cytokine detection. The assay was performed according to the manufacturer’s instructions. Details are available in the Supplementary Materials.

### ELISpot assays to quantify ASCs in blood, LN, and BM

ELISpot assays were performed as previously described (*23*, *38*). Details are provided in the Supplementary Materials.

### Viral stock

SARS-CoV-2 used in the challenge study was established as previously reported (*30*). SARS-CoV-2 (NR-52281: BEI Resources, Manassas, VA; USA-WA/2020, lot no. 70033175) was passaged on VeroE6 cells at a multiplicity of infection of 0.01 to produce the infectious viral stock. Additional details are provided in the Supplementary Materials.

### SARS-CoV-2 infection

RMs were infected under anesthesia with ~2.5 × 10^5^ PFU SARS-CoV-2 via both the IN (1 ml) and IT (1 ml) routes ~4 to 6 weeks after the third immunization. At each anesthetic access, pulse oximetry was recorded and RMs were clinically scored for responsiveness and recumbency; discharges; skin condition; respiration, dyspnea, and cough; food consumption; and fecal consistency.

### Sampling of virus in nasal, throat, and BAL

Nasopharyngeal (NP) swabs were collected under anesthesia by using a clean rayon-tipped swab (BactiSwab NPG, Thermo Fisher Scientific, R12300) placed about 2 to 3 cm into the nares. Oropharyngeal swabs were collected under anesthesia using polyester tipped swabs (Puritan Standard Polyester Tipped applicator, polystyrene handle, 25-806 2PD, VWR International) to streak the tonsils and back of throat bilaterally (throat/pharyngeal). The swabs were dipped in 1 ml of viral transport media (VTM; VTM-1L, Labscoop, LLC) and vortexed for 30 s, and the eluate was collected.

To collect BAL, under anesthesia, a fiberoptic bronchoscope (Olympus BF-XP190 EVIS EXERA III ULTRA SLM BRNCH and BF-P190 EVIS EXERA 4.1 mm) was manipulated into the trachea, directed into the primary bronchus, and secured into a distal subsegmental bronchus upon which 35 to 50 ml of normal saline (0.9% NaCl) was administered into the bronchus and reaspirated to obtain a minimum of 20 ml of lavage fluid. BAL was filtered through a 100-μm cell strainer.

### Quantifying VL RNA

SARS-CoV-2 genomic RNA was quantified in NP swabs, throat swabs, and BAL as recently described (*30*, *71*). Swabs were placed in 1 ml of VTM (Labscoop, LLC). Viral RNA was extracted from fresh specimens using the DSP Virus/Pathogen kit on Qia-Symphony SP. Additional details are provided in the Supplementary Materials.

### Histopathology

For histopathologic examination, lung samples were fixed in 4% neutral-buffered PFA for 24 hours at R.T., processed, paraffin-embedded, sectioned at 4 μm, and stained with H&E as previously described (*30*). The H&E slides from all tissues were examined by two board-certified veterinary pathologists. Additional details are provided in the Supplementary Materials.

### Statistics

Two-tailed nonparametric Mann-Whitney *U* test was used to test significance of differences observed in the magnitude of immune responses observed in assays used in the studies. A one-tailed nonparametric Mann-Whitney *U* test was used to test significance of reduction of pathology score in vaccinated animals in comparison with unvaccinated animals. A Wilcoxon signed-rank paired *t* test was used to compare significance of changes in frequencies of PBMCs in comparison with baseline frequencies when performing longitudinal studies of innate responses as well as in the investigation of anamnestic/recall response, if any, after virus challenge (prechallenge versus postchallenge time points). Spearman’s correlation coefficients and *P* values were calculated to assess immunological correlates. Analyses were performed using GraphPad Prism Version 8.0. RMAs using the mixed-effect linear model (*72*) and AUC measurements for VL changes over time (*73*, *74*) were performed separately for nasal swabs, BAL, and throat swabs for both total viral RNA and sgRNA. The AUC is a convenient and simple method to combine multiple readings into a single cumulative index. RMAs were also used to analyze longitudinal changes with Ab titers. Additional details on RMAs are available in the Supplementary Materials.

## Supplementary Material

20210715-1

## Notes

View/request a protocol for this paper from *Bio-protocol*.
