# Supplementary material for: A yeast-expressed RBD-based SARS-CoV-2 vaccine formulated with 3M-052-alum adjuvant promotes protective efficacy in non-human primates
Source: Sci Immunol. 2021 Jul 15;6(61):eabh3634. doi: 10.1126/sciimmunol.abh3634 (PMC9119307; doi:10.1126/sciimmunol.abh3634)
Supplement: 20210715-1 [file sciimmunol.abh3634.v1.pdf]

Cite as: M. Pino *et al.*, *Sci. Immunol.* 10.1126/sciimmunol.abh3634 (2021).

CORONAVIRUS

A yeast expressed RBD-based SARS-CoV-2 vaccine formulated with 3M-052-alum adjuvant promotes protective efficacy in non-human primates

Maria Pino[1*], Talha Abid[1*], Susan Pereira Ribeiro[2], Venkata Viswanadh Edara[1,3,4], Katharine Floyd[1,3,4], Justin C. Smith[5], Muhammad Bilal Latif[2], Gabriela Pacheco-Sanchez[2], Debashis Dutta[1], Shelly Wang[1], Sanjeev Gumber[2,6], Shannon Kirejczyk[2,6], Joyce Cohen[7], Rachelle L. Stammen[7], Sherrie M. Jean[7], Jennifer S. Wood[7], Fawn Connor-Stroud[7], Jeroen Pollet[8,9], Wen-Hsiang Chen[8,9], Junfei Wei[8,9], Bin Zhan[8,9], Jungsoon Lee[8,9], Zhuyun Liu[8,9], Ulrich Strych[8,9], Neeta Shenvi[10], Kirk Easley[10], Daniela Weiskopf[11], Alessandro Sette[11,12], Justin Pollara[13], Dieter Mielke[13], Hongmei Gao[13], Nathan Eisel[13], Celia C. LaBranche[13], Xiaoying Shen[13], Guido Ferrari[13], Georgia D. Tomaras[13], David C. Montefiori[13], Rafick P. Sekaly[1,2], Thomas H. Vanderford[1], Mark A. Tomai[14], Christopher B. Fox[15], Mehul S. Suthar[1,3,4], Pamela A. Kozlowski[5], Peter J. Hotez[8,9], Mirko Paiardini[1,2,#], Maria Elena Bottazzi[8,9,#] and Sudhir Pai Kasturi[1,2,#]

[1]Division of Microbiology and Immunology, Yerkes National Primate Research Center at Emory University, 954 Gatewood Rd, Atlanta, GA, U.S.A. [2]Department of Pathology and Laboratory Medicine, School of Medicine, Emory University, Atlanta, GA, U.S.A. [3]Emory Vaccine Center at Emory University, 954, Gatewood Rd, Atlanta, GA, U.S.A. [4]Centers for Childhood Infections and Vaccines; Children's Healthcare of Atlanta and Emory University, Department of Pediatrics, Atlanta, GA, U.S.A. [5]Department of Microbiology, Immunology, and Parasitology, Louisiana State University Health Sciences Center, New Orleans, Louisiana, U.S.A. [6]Division of Pathology, Yerkes National Primate Research Center, Emory University, Atlanta, GA 30329, USA. [7]Division of Animal Resources, Yerkes National Primate Research Center, Emory University, Atlanta, GA 30329, USA. [8]Texas Children's Center for Vaccine Development, Houston, TX, U.S.A. [9]Department of Pediatrics, Molecular Virology & Microbiology, National School of Tropical Medicine, Baylor College of Medicine, Houston, TX, U.S.A. [10]Department of Biostatistics and Bioinformatics, Emory University, Atlanta, GA, U.S.A. [11]Center for Infectious Disease and Vaccine Research, La Jolla Institute for Immunology (LJI), La Jolla, CA 92037, USA. [12]Department of Medicine, Division of Infectious Diseases and Global Public Health, University of California, San Diego (UCSD), La Jolla, CA 92037, USA. [13]Duke Human Vaccine Institute and Department of Surgery, Duke University Medical Center Durham, NC, USA. [14]3M Corporate Research Materials Laboratory, St. Paul, MN, USA. [15]The Infectious Disease Research Institute, Seattle, WA, USA.

*These authors contributed equally to the work.

*Corresponding author. Email: skastur@emory.edu (S.P.K.); bottazzi@bcm.edu (M.E.B.); mirko.paiardini@emory.edu (M.P.)

Ongoing SARS-CoV-2 vaccine development is focused on identifying stable, cost-effective, and accessible candidates for global use, specifically in low and middle-income countries. Here, we report the efficacy of a rapidly scalable, novel yeast expressed SARS-CoV-2 specific receptor-binding domain (RBD) based vaccine in rhesus macaques. We formulated the RBD immunogen in alum, a licensed and an emerging alum adsorbed TLR-7/8 targeted, 3M-052-alum adjuvants. The RBD+3M-052-alum adjuvanted vaccine promoted better RBD binding and effector antibodies, higher CoV-2 neutralizing antibodies, improved Th1 biased CD4+T cell reactions, and increased CD8+ T cell responses when compared to the alum-alone adjuvanted vaccine. RBD+3M-052-alum induced a significant reduction of SARS-CoV-2 virus in respiratory tract upon challenge, accompanied by reduced lung inflammation when compared with unvaccinated controls. Anti-RBD antibody responses in vaccinated animals inversely correlated with viral load in nasal secretions and BAL. RBD+3M-052-alum blocked a post SARS-CoV-2 challenge increase in CD14+CD16++ intermediate blood monocytes, and Fractalkine, MCP-1, and TRAIL in the plasma. Decreased plasma analytes and intermediate monocyte frequencies correlated with reduced nasal and BAL viral loads. Lastly, RBD-specific plasma cells accumulated in the draining lymph nodes and not in the bone marrow, contrary to previous findings. Together, these data show that a yeast expressed, RBD-based vaccine+3M-052-alum provides robust immune responses and protection against SARS-CoV-2, making it a strong and scalable vaccine candidate.

INTRODUCTION

Infection by beta coronaviruses (CoVs) in humans results in symptoms ranging from mild, caused by common cold-causing strains, to severe acute respiratory syndrome (SARS), caused by select highly virulent strains leading to death in many cases (*1*). COVID-19 is a respiratory infectious disease caused by SARS-CoV-2, a new CoV first reported in Wuhan, China in December 2019 (*2*–*4*). Due to rapid worldwide

spread, the World Health Organization (WHO) declared COVID-19 a pandemic in March 2020 with cases now over 175 million and greater than 3.5 million fatalities globally (*5*). An effective SARS-CoV-2 vaccine is the best way to stem the pandemic (*6*). A historic feat has been achieved in vaccinology by rapid design, testing and approval (< ~1 year) of several SARS-CoV-2 vaccines for use in select countries (*7*). However, there is a continued need for expanding the availability of safe, cost-effective, and scalable candidates for distribution in developing or low to middle-income countries (*8*). Additionally, there is an urgent need to improve cellular and molecular insights of SARS-CoV-2 vaccine-induced protective immunity in pre-clinical studies, preferentially in non-human primates (*7*, *9*).

SARS-CoV-2 infects host cells by binding of its surface transmembrane spike (S) protein to angiotensin-converting enzyme 2 (ACE2) receptors predominantly present on type II pneumocytes in the host respiratory mucosa (*10*). Thus, S protein is the lead target for vaccine design (*11*). Immunogens stabilized in their pre-fusion confirmation (*12*, *13*) elicit strong virus-neutralizing activity upon vaccination (*14*, *15*). Notably, the receptor-binding domain (RBD) in the S protein represents the most dominant target for neutralizing SARS-CoV-2 (*16*, *17*) and RBD targeted binding antibodies (Abs) highly correlate with virus-neutralizing activity (*18*). Several epitopes within RBD contribute to neutralization, thus both monomeric and oligomeric RBD vaccines are being tested (*16*). We have previously reported the protective efficacy of a yeast, *Pichia pastoris,* expressed RBD based vaccine against SARS-CoV in mice (*19*, *20*). The wide availability of yeast expression technology for hepatitis B vaccines in low and middle-income nations highlights a significant advantage for rapid manufacture of yeast-based SARS-CoV-2 vaccines, helping meet the need for inexpensive and accessible COVID-19 vaccines (*8*).

Unlike live attenuated virus-based vaccines, sub-unit vaccines are weakly immunogenic and require the use of adjuvants (*21*, *22*). Adjuvants substantially improve the quantity and quality of vaccine-induced immunity and can facilitate immunogen dose sparing. Pharmaceutically acceptable adjuvants are needed to strongly enhance Ab responses against SARS-CoV-2 protein immunogens. We recently reported that 3M-052, an emerging TLR-7/8 agonist formulated in polymer nanoparticles or adsorbed to alum (3M-052-alum) promotes the durability of HIV envelope (Env) specific long-lived plasma cells (LLPCs) in rhesus macaques (RMs); this correlated with serum Ab responses (*23*). In contrast, alum alone failed to induce LLPCs, an important finding considering that >40 sub-unit vaccines containing aluminum salts have been licensed for use (*24*). Additionally, vaccinating with 3M-052 and HIV-1 Env induced Ab response in the genital mucosa and promotes protection from viral challenge (*25*). As the respiratory mucosa is the primary port of entry of CoVs, inducing SARS-CoV-2 specific Ab responses in the serum and upper (URT) and lower respiratory tracts (LRT) is crucial. The induction of vaccine-specific LLPCs might support the longevity of such Ab responses. There is strong synergy between Ab and CD8+ T cell responses in promoting protective efficacy in NHPs against HIV-1 (*25*, *26*). These findings, along with the emergence of new variants of SARS-CoV-2 that may escape neutralization by Abs (*27*), further emphasizes the need for vaccines capable of inducing balanced CD8+ T cell and humoral immunity against SARS-CoV-2.

Here, we report the immunogenicity and protective efficacy of a novel, yeast expressed, SARS-CoV-2 derived RBD-219-WT (referred to as RBD immunogen in the study) (*28*) based vaccine formulated with adjuvants in RMs. We specifically combined RBD with alum or alum adsorbed 3M-052 (3M-052-alum), two clinically applicable adjuvants in inducing RBD specific humoral and T cell immunity (*29*). We challenged naïve control and vaccinated animals with live SARS-CoV-2 via the intranasal (IN) and intratracheal (IT) routes (*30*) and saw that protective immunity was induced only by the RBD+3M-052-alum vaccine, suggesting its promise as a SARS-CoV-2 vaccine candidate.

RESULTS

Study design and timeline: The SARS-CoV-2 RBD (amino acid residues 332-549 of the Spike protein S1 subunit) was cloned and expressed in the *Pichia pastoris* X-33 system (*20*) and homogeneity of the purified, protein was demonstrated by SDS-PAGE and SE-HPLC. The glycosylated protein migrated as a single band on non-reducing gels and eluted as a single peak from the HPLC column (fig. S1). We had three groups of RMs (n=5 per group), with one unvaccinated group (group 1), one group vaccinated with RBD + alum (group 2) and one with RBD+3M-052-alum (group 3, Fig. 1A). The RBD immunogen was used at 100 μg and alum at 500 μg of Al^{+3} content identical to our recent studies with HIV-1 envelope immunogens and guided by use in licensed vaccines (*31*, *32*). 3M-052 was used at 10μg per dose guided by ongoing human studies(NCT04177355). All animals in groups 2 and 3 received three immunizations at weeks 0, 4, and 9. In view of reports of a wide range of Ab responses achieved by vaccine platforms with varying vaccine doses and timing of immunizations (*33*–*37*), we reasoned that a third vaccination could substantially improve the magnitude and quality (higher neutralizing activity and effector function) as previously observed with HIV-1 immunogens (*23*, *38*). Blood, mucosal (nasal and rectal) swabs, BAL, and bone marrow samples were collected as detailed (Fig. 1B). All animals were challenged with the Washington-1 (WA-1), SARS-CoV-2 strain via a combined IN and IT route of administration approximately one month after the third vaccination. Animals were staggered for viral challenge in three separate groups and were

euthanized at days 7 or 8 after challenge. Blood, nasal, and throat swabs, as well as bronchoalveolar lavage (BAL) samples, were collected at pre and post-challenge time points, and at euthanasia (Fig. 1B).

The RBD+3M-052-alum vaccine induces robust humoral immunity in RMs.

We first investigated serological and mucosal humoral immunity induced by our vaccine. We observed onset of RBD binding Ab responses as early as 4 weeks after the first vaccination which was then substantially increased (~26-30 fold) at week 6 and week 11 after the first and second boost vaccinations in both groups (Fig. 2A). Binding Ab responses were significantly higher in group 3 when compared with group 2 at indicated time points (Fig. 2A) and were persistent in both groups prior to challenge. We next compared and quantified binding Abs to RBD, whole spike (S) and nucleocapsid (N), which ensured recognition of the RBD in a more native S protein-associated form. The kinetics of RBD and S specific Ab responses (fig. S2 A,B) were identical and there was no binding of Abs to N protein detected (fig. S2C). A correlation was observed between anti-RBD Ab responses (fig. S2D).

Live virus neutralizing titers were modest in all vaccinated animals after the 2nd dose, but were boosted to higher levels after the third vaccination (Fig. 2B). A positive correlation was found between RBD binding and live virus-neutralizing activity (Fig. 2C). Additionally, we assayed for neutralizing activity using the pseudovirus with the D614G mutation in the S protein found commonly in most circulating SARS-CoV-2 (*39*). Consistent with live virus neutralizing activity, pseudovirus nAbs were higher in both groups after the third vaccination. (Fig. 2D). We then screened for potential reduction in nAb responses against the spike-bearing pseudoviruses from the Alpha strain and the Beta strain, two SARS-CoV-2 variants of concern (VOCs) that emerged in the United Kingdom and South Africa in 2020 respectively (*27*, *40*). A marginal 1.8 and 1.4 fold reduction in nAb titer against the Alpha strain and a 9.2 fold and a 5.4 fold drop against the Beta strain was observed in serum of groups, 2 and 3 respectively at week 11 (Fig. 2E).

We then assayed for vaccine induced Ab subclasses (IgG1-4) at week 11 (*41*). A significantly higher anti-RBD IgG1 subclass response was induced in group 3 animals in contrast with significantly higher anti-RBD IgG4 isotype in group 2 animals suggesting a skewing to Th1 and Th2 responses by the respective adjuvants (Fig. 2F). Notably, there was also a consistent trend of higher non-neutralizing Ab effector function in group 3 animals using an antibody-dependent phagocytosis (ADP) (Fig. 2G) and a Spike protein-expressing cell Ab binding assay (SECABA) (fig. S2E) most likely contributed by the balance of Ab subclasses. Finally, using a new NK cell-mediated degranulation assay (fig. S3) reflecting antibody dependent cell cytotoxic (ADDC potential), we observed significantly higher responses in serum of animals in group 3 when compared with group 2 (Fig. 2H), and these ADCC responses positively correlated with the binding Ab titers (fig. S2F).

We next assayed for vaccine induced Ab responses in respiratory mucosa. We observed higher anti-RBD binding Ab responses in nasal secretions after the second vaccination at week 6, which continued to increase after the third vaccination most prominently in group 3 (Fig. 2I). IgG responses in nasal secretions positively correlated with live SARS-CoV-2 neutralization titers in serum (fig. S2G), suggesting a potential serum and not mucosal origin for these Abs. Anti-RBD binding Ab responses in BAL were modestly higher in group 3 animals vs. group 2 (Fig. 2J) and correlating with live SARS-CoV-2 neutralization titers in serum as well (fig. S2H). Finally, we observed significantly higher anti-RBD IgG Ab responses in rectal secretions (Fig. 2K), which also positively correlated with live SARS-CoV-2 neutralization titers in serum (Fig. 2SI). Overall, group 3 had better antibody responses compared to group 2, suggesting a positive effect of the 3M-052-adjuvant.

The RBD+3M-052-alum vaccine induces strong and durable RBD-specific CD8+ and Th1-biased CD4+ T cell responses in the blood

We next quantified vaccine specific CD4+ and CD8+ T cell responses in our study (fig. S4A). We observed induction of RBD-specific IFN-γ +CD8+ T cell responses as early as week 1 after the primary vaccination in a few animals (Fig. 3A,B). These responses peaked at week 10 with frequencies significantly higher in group 3 animals in comparison with those in group 2 and persisted on the day of challenge. A Th1 biased, RBD-specific IFN-γ+ CD4+ T cell response was also observed only in group 3 animals peaking at week 5 (Fig. 3C). Consistent with differences observed in RBD-specific IgG subclasses (Fig. 2F), we observed that the RBD-specific IL-4+CD4+ T cell response (Fig. 3D) trended higher in group 2 animals suggesting a Th2 biased response. Indeed, secretion of TNF, another key effector cytokine secreted at peak, was similar to levels of IFN-γ with both RBD-specific CD8+ and CD4+ T cell responses (Fig. S4B,C). In contrast, RBD-specific IL-2+CD4+ T cell responses peaked at the day of challenge (DOC) 4-6 weeks post final vaccination in group 3 animals (fig. S4C). Low levels of vaccine specific IL-17 production in CD4+ and CD8+ T cells was observed in select animals (fig. S4B,C). Overall, our data highlights that a balance in RBD-specific CD4+ and CD8+ T cell responses in addition to the RBD-specific Ab responses is achieved when using the RBD+3M-052-alum vaccine.

The RBD+3M-052-alum vaccine significantly reduces viral load in lower and upper respiratory tracts post SARS-CoV-2 challenge

RMs were challenged ~ a month after the final

immunization via the IN and IT routes with a total dose of ~2.3×10^5 PFU of live SARS-CoV-2, WA-1 isolate. Clinical parameters such as change in body weight, blood oxygen saturation and temperature on the day of and post-challenge until termination were closely monitored as detailed in Table S1. Moderate but no significant changes in these parameters were recorded in comparison with baseline including with clinical scores (cage-side assessment or physical examination under anesthesia), in any of the groups. No substantial change in comparison with baseline was recorded with serum chemistries post-challenge as detailed in Table S2. Both a linear mixed effects statistical model appropriate for repeated-measures analyses and the area under the curve (AUC) measurements were used for statistical inference of viral load changes over in nasal swabs, BAL and throat swabs for both total viral RNA and sgRNA as detailed in methods. Consistent with our recent report (*30*), all unvaccinated animals were infected with SARS-CoV-2, assessed by total RNA detected in BAL fluid at day 2 post challenge (Fig. 4A,B). In contrast, animals vaccinated with RBD+3M-052-alum were protected from SARS-CoV-2 infection by day 2, with 4/5 and all 5/5 animals having undetectable virus at days 4 and necropsy respectively. Animals vaccinated with RBD+alum in comparison with unvaccinated controls had lower VLs on day 4. Additionally, when comparing the overall mean differences between the area under the viral load curves (AUC), a significant difference was observed when comparing group 3 vs. 1 and not when comparing group 2 vs. 1. When sub-genomic (sg) RNA was measured, VL was detected in 100% of unvaccinated animals but in only 60% and 20% of groups 2 and 3 vaccinated animals respectively (Fig. 4C). Notably, only 1/5 animals in group 3 had a detectable VL at day 2 (Fig. 4D), once again significantly lower in comparison with control animals highlighting nearly complete protection at peak with group 3 animals. 4/5 and all 5/5 animals in group 3 had undetectable sgRNA at day 4 and necropsy, respectively. Similar to total RNA with overall AUC measurements, a significant difference again, was only observed between groups 3 and 1 and not when comparing groups 2 and 1. Furthermore, we observed reductions in VL in throat swabs (fig. S5) both with total and sgRNA in group 3 (fig. S5 A-D) where 60%, 80%, and 100% of animals had undetectable total and sgRNA at days 2,4 and 7/8 respectively. Total RNA VLs, were only significantly different in group 3 vs. unvaccinated animals, and no significant differences were observed when comparing group 2 vs. 1. We observed significant differences when comparing AUCs with both total and sgRNAs when comparing group 3 vs. 1 and not group 2. In summary, we observed a significant impact of vaccinating with RBD+3M-052-alum in reducing SARS-CoV-2 viral burden in the LRT.

Persistent total VL in nasal swabs was observed in 100% of control animals from days 1 through necropsy in contrast with rapid control of nasal VL observed by day 2 in many vaccinated animals (Fig. 4E, F). Notably, reduction in VL was again, most apparent when immunizing with RBD+3M-052-alum in comparison with unvaccinated controls. Identical results were observed when measuring sgRNA in nasal swabs with persistent replicating virus observed in 100% of control animals at days 2, 4 in contrast to vaccinated animals, most notably in group 3 where significant reduction of VL was observed at day 2 and 4 (Fig. 4G,H). In contrast with observations in LRT, VLs with both vaccinated groups in comparison with unvaccinated animals were significantly different when measured as AUC and sgRNA highlighting an impact of vaccination on reducing viral burden in the URT. Overall, these data support the use RBD+3M-052-alum vaccine to protect against SARS-CoV-2 infection.

The RBD+3M-052-alum vaccine reduces lung pathology after respiratory challenge with SARS-CoV-2

We next evaluated the impact of our vaccine in minimizing lung pathology post challenge. Lung pathology was determined using hematoxylin and eosin (H&E) staining after animal euthanasia (fig. S6A-C). Total pathology score was calculated considering severity and number of affected lobes while average pathology score was calculated measuring the average severity of abnormalities per affected lobe. The pathology score was reduced in lung sections of animals in group 3 vs. group 1 (Fig. S6D) but not in group 2 vs. 1 or when comparing group 2 vs. 3. Additionally, average pathology score, perivascular cuffing, inflammatory infiltrates, alveolar septal thickening, and type 2 pneumocyte hyperplasia were lower in lung sections of group 3 animals when comparing with those in group 1 (fig. S6E-I). Thus, group 3 RMs had reduced lung pathology compared to group 2 and 1.

Immune correlates of protection upon SARS-CoV-2 challenge in RMs

Ab responses and CD8+T cells have been reported to contribute to protecting against SARS-CoV-2 infection (*42*). Here, we correlated Ab and T cell responses to reduction in viral load both in URT and LRTs. SARS-CoV-2 nAbs, ADCC activity, and anti-RBD binding responses both in serum and nasal secretions at week 11 in the study negatively correlated with levels of total or sgRNA in nasal swabs at day 2 after challenge (Fig. 5A and fig. S7A). A negative correlation was found between anti-RBD Ab responses on the DOC with both total and sgRNA (Fig. 5B and S7B) consistent with the peak Ab responses. We observed no significant association between anti-RBD IFN-γ+CD8+ T or CD4+ T cell responses with total or sgRNA in nasal swabs (Fig. 5B). A negative correlation of nAb, and binding Ab responses was also observed with total RNA but not sgRNA in BAL, while ADCC activity positively correlated with both total and sgRNA (Fig. 5C, D and S7C,D). Anti-RBD-IFN- γ+CD8+ T responses did not correlate with total RNA in BAL and a correlation was found between RBD

specific CD4+ T cell response with total RNA in BAL on DOC (Fig. 5D). Overall, these data highlighted the importance of the induction of persistent anti-RBD serum and mucosal Ab responses in promoting protection against SARS-CoV-2.

The RBD+3M-052-alum vaccine reduces expansion of intermediate blood monocytes post SARS-CoV-2 challenge

Cytokine storm and infiltration of highly activated innate cells (neutrophils, monocytes and macrophages) contribute to lung pathology during SARS-CoV-2 infection in humans and RMs (*30*, *43*). Here, we evaluated changes in the frequencies of peripheral blood mononuclear cells (PBMCs) after SARS-CoV-2 challenge. An increase in frequencies of monocytes was most consistently observed in unvaccinated group 1 animals as assessed by complete blood counts (Table S3). Additionally, we used multi-parameter flow cytometry and identified subsets of blood monocytes as previously described (fig. S8) (*23*). Frequencies of intermediate monocytes increased by day 2 post-challenge most prominently in unvaccinated animals and persisted at higher levels till termination (Fig. 6A,B). In contrast, frequencies of intermediate monocytes were unchanged in vaccinated animals suggesting an impact of vaccine-induced immunity in blocking such an expansion. Also, there was a transient but notable increase in the frequency of blood plasmacytoid dendritic cells (pDCs) at day 1 post-challenge once again only in groups 1 and 2 with no significant changes in frequencies of myeloid DCs, lymphocytes, and NK cells in any groups (fig. S9 and S10).

We next measured 27 analytes in plasma of all animals post challenge (day 0-7/8) using a multiplexed MesoScaleDiscovery (MSD) platform. We first performed a PCA analysis (PC1:16.9%, PC2:16.1%) to evaluate the overall segregation of analytes in all animals upon challenge (fig. S11A). The contribution of analytes (cytokines/chemokines/interleukins and interferons) to each principal component is highlighted in fig.S11B. We performed a multivariate analysis using gap-statistics and K-mean clustering to identify the most prominent clusters in the entire data set (fig. S11C). A total of 12 clusters were identified as tabulated in Fig. 6C. Cluster 12, a combination of Fractalkine (chemokine ligand 1), monocyte chemotactic protein (MCP-1) and tumor necrosis factor (TNF)-related apoptosis inducing ligand or TRAIL was significantly up-regulated in group 1 animals while a significant reduction was observed in group 3 animals compared to groups 1 and 2. Notably, Cluster 8 with IL-18 and Cluster 5 composed of MIP.3b was selectively up-regulated in group 3 and 2 animals respectively highlighting distinct outcomes of vaccination in response to SARS-CoV-2 infection in RMs (Fig. 6C). A positive correlation was observed between Cluster 12 center vs. frequencies of intermediate monocytes at days 2 and 4 (Fig. 6D), a time point at which a peak viral load was observed in LRT and URT (Fig. 4 and fig. S4). Notably, baseline or day of challenge cluster 12 center did not correlate with viral sgRNA in nasal or throat swabs both at days 2 and 4, while a modest association was observed with sgRNA in BAL at day 2 and not with day 4 (Fig. S11D, E). In contrast, Cluster 12 center values positively correlated with the magnitude of sgRNA in nasal, BAL and throat at day 2 and 4, highlighting a potential plasma biomarker of SARS-CoV-2 virus levels in LRT and URTs (Fig. 6E and F). Notably, frequencies of intermediate monocyte also positively correlated with sgRNA in LRT and URT only at day 2 (fig. S11F) and not at day 4 (fig. S11G). A negative correlation of cluster 12 with classical monocytes was observed (fig. S12A). No correlation of cluster 12 with DC subsets or B cells was observed with viral sgRNA levels (Fig. S12B-F). Finally, both frequencies of intermediate monocytes as well as cluster 12 at day 2 and not day 4 post challenge positively correlated with lung pathology (fig. S13A and B); however, VLs post-challenge in the respiratory mucosa did not correlate with lung pathology, with the exception of BAL VL at day 2(fig. S13 C,D). In summary, we report a targeted and early impact of SARS-CoV-2 respiratory infection on blood monocytes which was strongly suppressed in animals vaccinated with RBD+3M-052-alum. We also report the identification of potential plasma biomarkers found in (Cluster-12), Fractalkine, MCP-1 and TRAIL, which correlated with respiratory levels of SARS-CoV-2.

RBD+3M-052-alum vaccine induced plasma cells accumulate in draining lymph nodes and not in the bone marrow

Vaccination of RMs with an HIV-based vaccine using the alum-3M-052 adjuvant compared to alum alone induced high frequencies of HIV-1 Env-specific ASCs in blood as well as successful homing and persistence of LLPCs both in the BM and LNs (*23*). Here, we quantified frequencies of RBD-specific ASCs first in peripheral blood at day 4 after the second and third vaccinations respectively, a time point previously established to be a peak for the presence of ASCs specifically in RMs (*23*, *38*). Higher frequencies of RBD-specific IgG+ and IgA+ ASCs were observed in group 3 animals relative to group 2, both after second and third immunizations (fig. S14A,B,C). We next quantified the frequencies of RBD-specific ASCs in BM aspirates. While RBD specific ASCs were detectable after the second vaccination at week 9, significantly higher frequencies of RBD specific ASCs in the BM were observed at week 11 when vaccinating with RBD+3M-052-alum compared to group 2 (Fig. 7 A, B, C). In contrast to the persistence observed with LLPCs induced by HIV-1 env immunogens in BM (*23*), we saw a sharp reduction in the frequencies of RBD-specific BM ASCs in aspirate samples between week 11 (peak) and week 13 (pre-challenge, Fig. 7B). Euthanizing animals a week post SARS-CoV-2 challenge allowed us to investigate the distribution of ASCs in vaccine draining LNs and in the femur bone marrow. Consistent with

our previous work in mice and NHPs, we observed a striking number of vaccine/RBD specific IgG+ ASCs in draining Iliac and popliteal LNs (Fig. 7D), while no responses were observed in the contralateral LNs (*23*, *44*). These responses were significantly higher in group 3 animals vs. group 2 (Fig. 7D). However, once again in sharp contrast to our HIV-1 data, we saw very low RBD-specific LLPCs in the femur derived BM (Fig. 7D). The lack of accumulation of RBD-specific LLPCs in the BM needs to be further investigated in the context of other SARS-CoV-2 vaccines.

The RBD+3M-052-alum vaccine induces higher expression of tissue homing chemokine markers on vaccine enriched blood ASCs

We next investigated the expression of chemokine markers on blood ASCs before and after the final vaccination using flow cytometry (*45*) (fig. S15) to understand the qualitative impact of adjuvants on vaccine enriched ASCs. ELISpot assays had revealed a significantly higher increase in RBD-specific IgG and IgA secreting cells at day 4 after the final vaccination at week 9 (fig. S14). Consistent with these data, here we saw significantly higher frequencies of CD38+CD80+ (all lineage-cells) cells when vaccinating with RBD+3M-052-alum in comparison with RBD+alum (Fig. 8A,B). Additionally, we also observed higher expression of chemokine receptors, CXCR3 (trafficking to inflamed tissue such as LNs) and CXCR4 (trafficking to the BM) (*46*, *47*) (fig. S15 and Fig. 8C,D), when vaccinating with RBD+3M-052-alum compared to alum alone. A modest increase in CCR7 expression was observed on ASCs when vaccinating with RBD+3M-052-alum and no upregulation of alpha4:beta7 was observed in any group (Fig. 8E,F). There was a positive correlation between blood ASCs in all vaccinated animals at day 4 with corresponding RBD specific IgG+ secreting cells (Fig. 8G). This highlighted the enrichment of vaccine specific ASCs. Frequencies of blood ASCs in all vaccinated animals at day 4, also positively correlated with RBD specific IgG+ASCs in lymph nodes (popliteal +iliac; combined) quantified at euthanasia (Fig. 8H) and with week 11 ASCs in bone marrow aspirates (Fig. 8I) but not with ASCs in long bone femur scoop tissue (Fig. 8J). Overall, these observations further inform on the qualitative impact of alum and 3M-052-alum adjuvants on chemokine receptor expression on vaccine enriched ASCs.

Finally, we evaluated anamnestic RBD-specific T and Ab responses after challenge. No appreciable change in frequencies of RBD-specific CD8+ and CD4+ T cells in comparison with pre-challenge was observed in PBMCs at the time of necropsy (Fig. S16A-F). No changes were also seen in the magnitude of anti-RBD binding Ab responses between the day of challenge and necropsy (Fig. S16G-I). RBD-specific CD8+ and CD4+ T cell responses were also assayed in hilar LNs that drain the lung. No SARS-CoV-2 specific CD8+ (Fig. S17) and CD4+ T cell responses (Fig. S18) were detected in hilar LNs of naïve control animals in group 1 at necropsy. Significantly higher CD8+ T cell and CD4+ T cell responses were observed only in group 3 animals suggesting these responses were likely induced by vaccination and not by the SARS-CoV-2 challenge. Overall, these data inform on the tissue distribution of vaccine-induced B and T cell responses against SARS-CoV-2.

DISCUSSION

Many SARS-CoV-2 vaccine candidates are based on the whole S protein and limited testing in NHPs has been reported with RBD-based vaccines (*7*, *36*, *48–50*). Since RBD targeted binding Abs correlate very strongly with virus-neutralizing activity in natural infections and vaccinations (*18*), an RBD immunogen offers a target for rational vaccine design both immunologically and from a manufacturability point of view (*8*). Our study highlights induction of robust anti-SARS-CoV-2 Ab responses and protection from SARS-CoV-2 infection and pathogenesis specifically when using the RBD+3M-052-alum vaccine formulation.

Consistent with previous reports of live SARS-CoV-2 nAbs induced by chimpanzee adenovirus (ChAdOx-1) (*33*) or with the adenovirus 26 (Ad.26) based viral vectored vaccines in NHPs (*49*), nAbs were observed after two vaccinations in our study. However, these titers were inferior when compared with whole S immunogen based mRNA vaccines after two vaccinations (*36*). One explanation for this outcome is the presence of additional neutralizing and non-neutralizing epitopes contributing to overall higher titers when using a whole S immunogen vs. RBD. Clinical sub-unit vaccines and pre-clinical studies in NHPs have documented significant increases in magnitude (peak) and quality of Ab responses against protein vaccines after three vaccinations (*23*, *38*). We report higher live SARS-CoV-2 nAbs and pseudovirus nAb in group 3 animals after the third vaccination with a relatively lower increase in binding Abs. Additionally, a smaller drop observed in cross neutralizing activity against the Beta strain selectively in animals vaccinated with RBD-3M-052-alum suggests potential maturation of Ab responses. We have recently reported strong germinal center (GC) responses with repeated vaccinations when using 3M-052 and HIV-1 Env immunogens (*23*) which could have contributed to improved nAbs after the third vaccination. Such induction of GC's with RBD vaccination needs to be investigated in future studies. While, a role for non-neutralizing Abs in protection with vaccine-induced immunity is unclear, treatment with monoclonal Abs in therapeutic settings highlights benefits with Fc effector functions in reducing lung pathology (*51*). Based on these reports, we hypothesize that anti-RBD Abs were skewed by adjuvants toward IgG1 (higher effector function in macaques in comparison with IgG3) or IgG4 subclasses (*41*), followed by strong ADCC activity that correlates with VLs in mucosa as observed in groups 3 or 2 respectively, may

differentially contribute to protection.

A protective SARS-CoV-2 vaccine should ideally induce a protective mucosal response. We observed strong anti-RBD Ab responses in nasal, BAL and rectal secretions when vaccinating via the IM route, which may contribute substantially to minimizing transmission and onset of disease followed by a reduction in fecal shedding of virus respectively. It is unknown whether an IN route of vaccination will further improve mucosal immunity with local IgA Abs. Most SARS-CoV-2 vaccines approved under emergency use authorization follow a two dose regimen. Since we observed protective efficacy after three vaccinations, additional investigation including testing in humans with two vaccine doses is critical to assess protective efficacy. Notably, robust induction of cross-CoV neutralizing Ab responses in RMs when vaccinating with a multimeric RBD displaying nanoparticle immunogen with the 3M-052-alum has recently been reported (*52*). These data are consistent with strong immunogenicity of multimeric nanoparticle protein-based construct used in the Novavax vaccine adjuvanted with Matrix-M (*53*). Collectively, we conclude that 3M-052-alum may offer significant promise in improving immunogenicity of both monomeric and multimeric RBD based SARS-CoV-2 vaccines.

Robust CD8+ T cell responses, nAbs and Th1 biased CD4+T cells could also support protective immunity against SARS-CoV-2 (*54*). Specifically, where nAb responses can be sub-optimal against emerging variants of concern (VOCs) (*27*), conserved epitope-based CD8+ T cell responses could support protection. No evidence of induction of robust CD8+ T cell responses was observed when using the recently approved mRNA-1273 vaccine from Moderna, inactivated SARS-CoV-2 from Sinovac or a recombinant protein from Novavax in NHPs (*48*, *53*, *55*). Of note, the saponin-based adjuvant used in the Novavax vaccine induces CD8+ T cell responses with protein immunogens in mice (*56*). To date, SARS-CoV-2 specific CD8+ T cells have only been induced by viral vectored ChAd0X-1 and Ad.26/and or Ad5 in NHPs or with the BNT162b2, a mRNA vaccine in humans (*7*). Induction of anti-RBD CD8+ T cell responses in our current study is in contrast to our inability to consistently induce either anti-simian immunodeficiency (SIV) gag or anti-Env CD8+ T cell responses in RMs with HIV-1 or SIV immunogens when using TLR-7/8 agonist-based adjuvants (*23*, *38*). It is possible that the RBD immunogen is enriched in epitopes for CD8+ T cells or preferentially targets cross-presenting DCs. However, as discussed above, RBD-containing whole S protein-based vaccines expressed in mammalian cells do not induce appreciable CD8+ T cell responses. Alternatively, unique glycosylation patterns of a yeast expressed RBD immunogen (*20*) that could improve targeting of lectin receptors on DC subsets (*57*) including monocyte-derived inflammatory DCs (*58*), may better induce CD8+ T cells. Indeed, this hypothesis is well supported by; a) reported addition of distinct numbers of mannose residues when expressing proteins in mammalian (~8 mannose residues) vs. yeast hosts(up to 20 mannose residues) (*59*) and b) that mannose receptor mediates uptake of soluble mannose expressing antigens for cross-presentation (*60*). In summary, yeast expression of RBD in combination with a Th1 biasing 3M-052 adjuvant likely led to targeting and differentiation of inflammatory DCs, supporting the induction of CD8+ T cells that may improve vaccine efficacy against emerging SARS-CoV-2 variants.

Differentiation of classical to intermediate monocytes has been documented in Dengue virus infections in humans (*61*), as well as when using TLR-7/8 adjuvants in RMs (*23*, *38*). Intermediate monocytes favor B cell differentiation to antibody-secreting cells (ASCs) via secretion of IL-10 and B cell-activating factor (BAFF) (*61*). SARS-CoV-2 is a single-stranded (ssRNA) virus with potential to target TLR-7/8 receptors. Our data supports that respiratory infection with SARS-CoV-2 leads to activation of blood monocytes. Precise immunological mechanisms by which either anti-RBD Ab or T cell responses induced by RBD+3M-052-alum vaccine in respiratory mucosa contribute to blocking activation of peripheral blood monocytes are unclear. We hypothesize that anti-RBD Ab responses in nasal and BAL mucosa could be contributing to rapid clearance of virus via formation of immune complexes and perhaps signaling via Fc inhibitory receptors on innate immune cells as seen with mAbs in therapeutic settings (*51*). Indeed, a larger role for monocytes/macrophages in BAL after SARS-CoV-2 challenge has been reported in both humans and RMs (*30*, *43*). Identification of cluster 12 comprising of monocyte-related analytes (Fractalkine, MCP-1 and TRAIL) in plasma further supports a targeted impact of SARS-CoV-2 infection on monocyte recruitment to lung respiratory mucosa contributing to exaggerated pathogenicity of COVID-19.

Currently, there is limited knowledge on the ability of SARS-CoV-2 vaccines to induce BM homing LLPCs in NHPs or humans which is predictive of the longevity of Ab responses. Here, we document a transient presence of RBD-specific ASCs in RM BM aspirate after the third vaccination that contracts rapidly between weeks 11 and 13 in contrast with our previous work with adjuvanted HIV-1 immunogen specific BM LLPCs (*23*). We also previously documented that repeated BM sampling can deplete finite numbers of plasma cells in aspirates (*23*), and hence additionally verified the presence of RBD-specific LLPCs in the femur BM at necropsy. In contrast with our studies using HIV-1 immunogens, we observed low levels of RBD specific LLPCs in the femur BM scoop tissue. However, we documented robust frequencies of RBD-specific ASCs in both Iliac and popliteal LNs draining the vaccination site consistent with our previous observations both in mice and RMs (*23*, *44*). While quantifying the

relative contributions of LN vs. BM resident LLPCs in supporting Ab responses is challenging, it is reasonable to hypothesize that draining LNs may provide an alternate site of persistence of SARS-CoV-2 vaccine-specific LLPCs that could support durability of RBD specific Ab responses. Increased expression of CXCR3 and higher CCR7 on blood ASCs when vaccinating with RBD+3M-052-alum may support trafficking to inflamed draining LNs. Alternatively, a significant proportion of LN ASCs could have also failed to egress into the periphery. While increased CXCR4 expression on blood ASCs may in part explain transient homing of blood ASCs to the BM, a sharp contraction in frequencies of RBD specific ASCs in the BM suggests a short life span or poor quality of these cells. These findings highlight the need for longitudinal tracking of SARS-CoV-2 plasma cells in parallel with serum Ab responses.

We face continued challenges with supply, distribution under extreme cold-chain requirements and availability of SARS-CoV-2 vaccines in low-income or developing countries. We reasoned that a vaccine design based on a low-cost production process matching existing platforms such as with the manufacture of hepatitis B vaccines could be easily repurposed for local production and meeting global requirements of a SARS-CoV-2 vaccine. Our study supports the testing of an RBD-based immunogen adjuvanted with 3M-052-alum in human trials might offer as a cost-effective, scalable and thermostable SARS-CoV-2 vaccine.

MATERIALS AND METHODS

Study design

The objective of our study was to establish the immunogenicity and protective efficacy of a SARS-CoV-2 derived yeast expressed RBD monomer immunogen. We formulated RBD with two clinically relevant adjuvants in alum and 3M-052-alum. Using the RM model (n=5/treatment group), we tested immunogenicity and protective efficacy of this vaccine upon a respiratory challenge with live SARS-CoV-2 as detailed in Fig. 1. We investigated correlation of vaccine induced Ab and T cell responses with viral loads in the respiratory mucosa upon challenge. Additionally, we evaluated changes in RM blood PBMCs and soluble analytes post-challenge and correlation of such blood parameters with viral loads in the respiratory mucosa. Finally, we investigated the distribution of vaccine specific plasma cells in blood, LNs and BM.

Cloning and expression of SARS-CoV-2 RBD in the yeast *Pichia pastoris*

As recently described (*20*), RBD219-WT in *P. pastoris X33* was produced by fermentation at the 5 L scale and the target protein was purified through a combination of anion exchange, hydrophobic interaction, and size-exclusion chromatography. Additional details are provided in supplementary materials.

Adjuvants and formulations: 3M-052, a TLR-7/8 agonist was provided by 3M Corporate Research and Materials Lab (MN, U.S.A). Aluminum oxyhydroxide (Alhydrogel '85') was procured from Brenntag Biosector, Denmark and formulated and aliquoted for use at The Infectious Disease Research Institute (IDRI). 3M-052 adsorption to alum was facilitated by first preparing a lipid nanosuspension with 3M-052 and distearoyl phosphatidylglycerol (DSPG) at a 1:4 3M-052:DSPG molar ratio which was then mixed with aluminum oxyhydroxide following a procedure adapted from our previous report (*62*).

Animals: Fifteen rhesus macaques, aged ~ 5-7 years (all male) were identified for the study from the Yerkes National Primate Research Center colony. YNPRC's animal care facilities are accredited by both the U.S. Department of Agriculture (USDA) and by the Association for Assessment and Accreditation of Laboratory Animal Care (AAALAC). All animal procedures were performed in accordance with guidelines established by the Emory University Institutional Animal Care and Use Committee Guidelines and those set up by the NIH's Guide for the Care and Use of Laboratory Animals, 8th edition.

Immunization details: Animals were immunized three times in the right calf muscle via the intramuscular (I.M) route of vaccination at weeks 0, 4, and 9 as detailed in the study timeline in Fig. 1. RBD-WT was used at 100µg, alum was used at 500 µg and 3M-052 was used at 10 µg per dose. RBD immunogen stored at -80°C was thawed and mixed with alum or 3M-052-alum adjuvant formulations for 20 min at room temperature (R.T) on an end-to-end shaker to allow adsorption. Saline was used as the diluent to make up volumes and all animals were immunized with 1.0ml of the final inoculum. All immunizations and samplings were performed under sedation with ketamine and/or Telazol.

Analyses of anti-RBD IgG binding Ab responses: RBD-specific binding Abs were assayed using a recently described assay with minimal modifications (*63*). Briefly, RBD coated ELISA plates were incubated with serially diluted sera from pre-vaccination and vaccinated time points, followed by detection using horse radish peroxidase conjugated anti-monkey Ab (SB108A, Southern Biotech, Al, USA) and absorbance analyses post incubation with Tetramethyl benzidine (TMB) substrate. Additional details are included in supplementary materials.

Multiplexed analyses of anti-RBD, spike and nucleocapsid Ab responses:

Serum IgG binding to SARS-CoV-2 Spike, RBD, and nucleocapsid proteins was evaluated using the 4-PLEX SARS-CoV-2 Panel 2 Kit from Meso Scale Discovery (MSD). Briefly, samples were incubated at multiple dilutions on the plates preprinted with SARS-CoV-2 antigens, followed by detection with SULFO-Tag labeled anti-human IgG antibody.

Electrochemiluminescence (ECL) signal from each sample against each antigen was quantified by applying electricity to electrodes built-in assay plates. The magnitude of binding, evaluated as arbitrary units (AU/mL), was calculated by back-fitting the binding ECL on the 4-parameter logistic regression model which was generated by titration of the kit's reference standard with assigned AU concentration.

Pseudovirus neutralizing assay*:*

SARS-CoV-2 neutralization was assessed with Spike-pseudotyped virus in 293T/ACE2 cells as a function of reductions in luciferase (Luc) reporter activity. 293T/ACE2 cells were kindly provided by Drs. Mike Farzan and Huihui Mu at Scripps Research Institute. Cells were maintained in DMEM containing 10% FBS, 25 mM HEPES, 50 µg/ml gentamycin and 3 µg/ml puromycin. An expression plasmid encoding codon-optimized full-length Spike of the Wuhan-1 strain (VRC7480), was provided by Drs. Barney Graham and Kizzmekia Corbett at the Vaccine Research Center, National Institutes of Health (USA). The D614G amino acid change was introduced into VRC7480 by site-directed mutagenesis using the QuikChange Lightning Site-Directed Mutagenesis Kit from Agilent Technologies (Catalog # 210518). The mutation was confirmed by full-length Spike gene sequencing. Pseudo virions were produced in HEK 293T/17 cells (ATCC cat. no. CRL-11268) by transfection using Fugene 6 (Promega Cat#E2692) and a combination of Spike plasmid, lentiviral backbone plasmid (pCMV ΔR8.2) and firefly Luc reporter gene plasmid (pHR' CMV Luc) in a 1:17:17 ratio. Transfections were allowed to proceed for 16-20 hours at 37°C. Media was removed, monolayers rinsed with growth medium, and 15 ml of fresh growth medium added. Pseudovirus-containing culture medium was collected after an additional 2 days of incubation and was clarified of cells by low-speed centrifugation and 0.45 µm micron filtration and stored in aliquots at -80°C. TCID50 assays were performed on thawed aliquots to determine the infectious dose for neutralization assays (RLU 500-1000x background, background usually averages 50-100 RLU).

For neutralization, a pre-titrated dose of virus was incubated with 8 serial 5-fold dilutions of serum samples in duplicate in a total volume of 150 µl for 1hr at 37oC in 96-well flat-bottom poly-L-lysine-coated culture plates (Corning Biocoat). Cells were suspended using TrypLE Select Enzyme solution (Thermo Fisher Scientific) and immediately added to all wells (10,000 cells in 100 µL of growth medium per well). One set of 8 control wells received cells + virus (virus control) and another set of 8 wells received cells only (background control). After 66-72hrs of incubation, the medium was removed by gentle aspiration and 30 µL of Promega 1X lysis buffer was added to all wells. After a 10 min incubation at room temperature, 100 µl of Bright-Glo luciferase reagent was added to all wells. After 1-2 min, 110 µl of the cell lysate was transferred to a black/white plate (Perkin-Elmer). Luminescence was measured using a PerkinElmer Life Sciences, Model Victor2 luminometer. Neutralization titers are the serum dilution at which relative luminescence units (RLU) were reduced by either 50% (ID50) or 80% (ID80) compared to virus control wells after subtraction of background RLUs. Serum samples were heat-inactivated for 30 min at 56°C prior to assay.

Live SARS-CoV-2 neutralizing or focus reduction neutralization titer (FRNT) assay:

Neutralization assays with authentic SARS-CoV-2 virus were performed as previously described (*64*). Briefly, serially diluted sera from vaccinated animals incubated (1 hour) with 100-200FFU infectious clone derived SARS-CoV-2-mNG (*65*) was added to VeroE6 cell monolayer for an hour. After removal of inoculum and incubation with 0.85% methylcellulose containing DMEM for 24 hours, cells were fixed with 2% PFA for 30 min, washed and foci were visualized on a fluorescence ELISpot plate reader (*66*). Additional details on the assay and calculation of nAb titers are detailed in supplementary materials.

ELISA for anti-RBD IgG subclasses

Immulon 4 plates were coated overnight with RBD, except for 2 rows dedicated for standard. These wells were coated with either purified IgG1, IgG2, IgG3 or IgG4 (all from the NHP Reagent Resource). Plates loaded with serum samples were similarly reacted overnight. Plates were treated with 1µg/ml of the following respective monoclonal antibodies to rhesus IgG1, IgG2, IgG3 and IgG4 (all from the NHP Reagent Resource): 3C10.3, 3C10.1, 2G11 and 7A8. After 1h at 37°C, the rhesus subclass-specific mAb was detected with either SBA #1082-08 biotinylated goat anti-mouse IgG2a (for 3C10.3) or SBA #1071-08 biotinylated goat anti-mouse IgG1 (for 3C10.1, 2G11, and 7A8). Plates were then washed and reacted with 1 in 2,000 diluted neutralite avidin-peroxidase (SBA#7200-05) for 30 min at room temperature, washed and developed with TMB (SBA #0410-01).

ELISA for anti-RBD IgG in secretions

Immulon 4 microtiter plates (Fisher) were coated overnight with 100ng per well of the RBD immunogen in PBS. Plates were then washed with PBS containing 0.05% Tween20 (PBST) and blocked for 30min with 0.1% BSA in PBST. Standard and samples diluted in fresh block buffer were then added. The IgG standard was an anti-RBD human IgG antibody (ACRO #SAD-S35). Following overnight storage at 4°C, plates were washed and treated with biotinylated anti-human g chain antibody (SBA#2048-08) for 1h at 37°C. Plates were then washed and reacted with 1/2,000 neutralite avidin-peroxidase (SBA #7200-05) for 30min at room temperature. Plates were washed and developed with TMB (SBA #0410-01). Absorbance was recorded at 370nm after 30min.

Concentrations of antibody were subsequently interpolated from standard curves constructed with SoftMax Pro software (Molecular Devices). For secretions, concentrations of antigen-specific IgG antibodies were normalized by dividing by the concentration of total IgG, measured by ELISA.

ELISA for total IgG in secretions

Immulon 4 plates were coated overnight with 50ng per well of goat anti-monkey IgG (Rockland). Plates were then washed, blocked and loaded with serially diluted secretions. The standard was rhesus IgG (Rockland). Plates were developed as described above with anti-RBD IgG in secretions using biotinylated goat anti-human -g chain antibody (SBA).

Antibody-dependent phagocytosis

Phagocytosis assays were performed as previously described (*67*). Briefly, 1μm avidin-coated fluorescent beads (Molecular Probes) were labeled with biotinylated anti-HIS tag antibody followed by the HIS-tagged RBD immunogen. Beads (25μl) were pre-incubated at 37°C in V-bottom plates with diluted serum samples (25μl). After 1h, 2 × 104 THP-1 cells (in 50μl) were added to every well. After 5h at 37°C in 5% CO2, the cells were washed in DPBS (lacking Ca+2 and Mg+2) then treated with trypsin for 5 min. The cells were washed and resuspended in 1% paraformaldehyde. Fluorescence was analyzed using a FACS Canto (BD Biosciences) and FlowJo software (BD) was used to determine the % of bead+ cells and multiply them by their median fluorescence intensity. A phagocytic score was calculated for each test sample by dividing this value by the average value obtained for 4 RBD naïve sera at the same dilution. A score of 2.0 was considered significant.

Spike protein-expressing cell antibody binding assay (SECABA)

The cell antibody binding assay was performed based on our previously described methods (*68*–*70*) modified to use target cells transfected with plasmids designed to express the SARS-CoV-2 Spike protein (S-protein) with a c-terminus flag tag. Serum from vaccinated animals was serially diluted and incubated with S expressing transfected cells and % rhesus IgG+ cells were quantified as detailed in supplementary materials.

Antibody-dependent NK cell degranulation assay. Cell-surface expression of CD107a was used as a marker for NK cell degranulation, a prerequisite process for ADCC (*71*), performed as previously described (*72*). 293 target T cells transfected with the S protein from G614 variant were incubated at a 1:1 ratio with NK cells from healthy volunteers in the presence of absence of test sera for 6 hours at 37°C. Cell surface expression of CD107a was used to assess NK cell degranulation or ADCC like activity as detailed in supplementary materials.

T cell stimulation and intracellular cytokine staining (ICS) assays:

The assay was performed as described before (*23*). Briefly, ~2 × 10^6 PBMCs, were cultured in 200μl final volume in 5ml polypropylene tubes (BD Biosciences, San Diego, CA, U.S.A) in the presence of anti-CD28 (1μg/ml) and anti-CD49d (1μg/ml) [BD Biosciences] and the following conditions; a) negative control with DMSO only, b) Whole spike (S) peptide pool 1 (n=253 peptides, 15mers with 10 residue overlap), (Weiskopf and Sette labs, LJI, La Jolla, CA) at a final concentration of 1μg/ml, c) RBD peptide pool 2 (53 peptide pool, 15mers with 11 residue overlap, JPT Peptide Technologies, Germany) at a final concentration of 1μg/ml, d) N peptide pool (only pre-challenge and post challenge time points) and e) PMA/Ionomycin. Brefeldin A was added to all tubes at 10μg/ml (Sigma-Aldrich, St Louis, MO) and cells were cultured for an 6 hours and transferred to 4° before staining for flow cytometry as detailed in the supplementary materials.

PBMC staining for flow cytometry post-challenge:

PBMCs were stained as described before (*23*) and detailed in the supplementary materials.

MSD Cytokine Assay

Meso Scale. U-PLEX assay (Meso Scale MULTI-ARRAY Technology) commercially available by Meso Scale Discovery (MSD) was used for plasma cytokine detection. The assay was performed according to the manufacturer's instructions. Details are available in the supplementary materials.

ELISpot assays to quantify ASCs in blood, LN and BM . ELISpot assays were performed as previously described (*23*, *38*). Details are provided in supplementary materials.

Viral stock

SARS-CoV-2 used in the challenge study was established as previously reported (*30*). SARS-CoV-2 (NR-52281: BEI Resources, Manassas, VA; USA-WA/2020, Lot no. 70033175) was passaged on Vero E6 cells at a MOI of 0.01 to produce the infectious viral stock. Additional details are provided in supplementary materials.

SARS-CoV-2 infection

RMs were infected under anesthesia with ~2.5x10^5 plaque forming units (PFU) SARS-CoV-2 via both the intranasal (1 mL) and intratracheal (1 mL) routes ~4-6 weeks after the third immunization. At each anesthetic access pulse oximetry was recorded and RMs were clinically scored for responsiveness and recumbency; discharges; skin condition; respiration, dyspnea, and cough; food consumption; and fecal consistency.

Sampling of virus in nasal, throat and bronchoalveolar lavage (BAL). Nasopharyngeal swabs were collected under anesthesia by using a clean rayon-tipped swab (ThermoFisher Scientific, BactiSwab NPG, R12300) placed approximately 2-3cm into the nares. Oropharyngeal swabs were collected under anesthesia using polyester tipped swabs

(Puritan Standard Polyester Tipped applicator, polystyrene handle, 25-806 2PD, VWR International) to streak the tonsils and back of throat bilaterally (throat/pharyngeal). The swabs were dipped in 1 mL viral transport media (Viral transport Media, VTM-1L, Labscoop, LLC) and vortexed for 30 s, and the eluate was collected.

To collect BAL, under anesthesia, a fiberoptic bronchoscope (Olympus BF-XP190 EVIS EXERA III ULTRA SLM BRNCH and BF-P190 EVIS EXERA 4.1mm) was manipulated into the trachea, directed into the primary bronchus, and secured into a distal subsegmental bronchus upon which 35-50 mL of normal saline (0.9% NaCl) was administered into the bronchus and re-aspirated to obtain a minimum of 20ml of lavage fluid. BAL was filtered through a 100µm cell strainer.

Quantifying viral load RNA: SARS-CoV-2 genomic RNA was quantified in nasopharyngeal (NP) swabs, throat swabs, and BAL as recently described (*30*, *73*). Swabs were placed in 1mL of Viral Transport Medium (VTM; Labscoop, LLC). Viral RNA was extracted from fresh specimens using the DSP Virus/Pathogen kit on Qia-Symphony SP. Additional details are provided in the supplementary materials.

Histopathology:

For histopathologic examination, lung samples were fixed in 4% neutral-buffered paraformaldehyde for 24h at room temperature, processed, paraffin-embedded, sectioned at 4µm, and stained with hematoxylin and eosin (H& E) as previously described (*30*). The H&E slides from all tissues were examined by two board certified veterinary pathologists. Additional details are provided in supplementary materials.

Statistics: Two-tailed non-parametric Mann-Whitney U test was used to test significance of differences observed in the magnitude of immune responses observed in assays used in the studies. A one tailed non-parametric Mann-Whitney U test was used to test significance of reduction of pathology score in vaccinated animals in comparison with unvaccinated animals. A Wilcoxon-signed rank paired *t* test was used to compare significance of changes in frequencies of PBMCs in comparison with baseline frequencies when performing longitudinal studies of innate responses as well as in the investigation of anamnestic/recall response if any post virus challenge (pre-challenge vs. post-challenge time points). Spearman's correlation coefficients and p values were calculated to assess immunological correlates. Analyses were performed using GraphPad PRISM version 8.0. Repeated measures analyses (RMA) using the mixed-effect linear model (*74*) and area under the curve measurements for viral load changes over time (*75*, *76*) were performed separately for nasal swabs, BAL and throat swabs for both total viral RNA and sgRNA. The AUC is a convenient and simple method to combine multiple readings into a single cumulative index. RMA was also used to analyze longitudinal changes with Ab titers. Additional details on RMA are available in supplementary materials.

SUPPLEMENTARY MATERIALS

immunology.sciencemag.org/cgi/content/full/6/61/eabh3634/DC1
Supplemental Materials and Methods
Figs. S1 to S18
Tables S1 to S4

REFERENCES AND NOTES

1. T. S. Fung, D. X. Liu, Human Coronavirus: Host-Pathogen Interaction. *Annu. Rev. Microbiol.* **73**, 529–557 (2019). doi:10.1146/annurev-micro-020518-115759 Medline
2. N. Zhu, D. Zhang, W. Wang, X. Li, B. Yang, J. Song, X. Zhao, B. Huang, W. Shi, R. Lu, P. Niu, F. Zhan, X. Ma, D. Wang, W. Xu, G. Wu, G. F. Gao, W. Tan; China Novel Coronavirus Investigating and Research Team, A Novel Coronavirus from Patients with Pneumonia in China, 2019. *N. Engl. J. Med.* **382**, 727–733 (2020). doi:10.1056/NEJMoa2001017 Medline
3. P. Zhou, X. L. Yang, X. G. Wang, B. Hu, L. Zhang, W. Zhang, H. R. Si, Y. Zhu, B. Li, C. L. Huang, H. D. Chen, J. Chen, Y. Luo, H. Guo, R. D. Jiang, M. Q. Liu, Y. Chen, X. R. Shen, X. Wang, X. S. Zheng, K. Zhao, Q. J. Chen, F. Deng, L. L. Liu, B. Yan, F. X. Zhan, Y. Y. Wang, G. F. Xiao, Z. L. Shi, A pneumonia outbreak associated with a new coronavirus of probable bat origin. *Nature* **579**, 270–273 (2020). doi:10.1038/s41586-020-2012-7 Medline
4. Coronaviridae Study Group of the International Committee on Taxonomy of Viruses, The species Severe acute respiratory syndrome-related coronavirus: Classifying 2019-nCoV and naming it SARS-CoV-2. *Nat. Microbiol.* **5**, 536–544 (2020). doi:10.1038/s41564-020-0695-z Medline
5. E. Dong, H. Du, L. Gardner, An interactive web-based dashboard to track COVID-19 in real time. *Lancet Infect. Dis.* **20**, 533–534 (2020). doi:10.1016/S1473-3099(20)30120-1 Medline
6. B. F. Haynes, L. Corey, P. Fernandes, P. B. Gilbert, P. J. Hotez, S. Rao, M. R. Santos, H. Schuitemaker, M. Watson, A. Arvin, Prospects for a safe COVID-19 vaccine. *Sci. Transl. Med.* **12**, eabe0948 (2020). doi:10.1126/scitranslmed.abe0948 Medline
7. L. Grigoryan, B. Pulendran, The immunology of SARS-CoV-2 infections and vaccines. *Semin. Immunol.* **50**, 101422 (2020). doi:10.1016/j.smim.2020.101422 Medline
8. P. J. Hotez, M. E. Bottazzi, Developing a low-cost and accessible COVID-19 vaccine for global health. *PLOS Negl. Trop. Dis.* **14**, e0008548 (2020). doi:10.1371/journal.pntd.0008548 Medline
9. M. Connors, B. S. Graham, H. C. Lane, A. S. Fauci, SARS-CoV-2 Vaccines: Much Accomplished, Much to Learn. *Ann. Intern. Med.* **174**, 687–690 (2021). doi:10.7326/M21-0111 Medline
10. A. C. Walls, Y. J. Park, M. A. Tortorici, A. Wall, A. T. McGuire, D. Veesler, Structure, Function, and Antigenicity of the SARS-CoV-2 Spike Glycoprotein. *Cell* **183**, 1735 (2020). doi:10.1016/j.cell.2020.11.032 Medline
11. D. Wrapp, N. Wang, K. S. Corbett, J. A. Goldsmith, C. L. Hsieh, O. Abiona, B. S. Graham, J. S. McLellan, Cryo-EM Structure of the 2019-nCoV Spike in the Prefusion Conformation. *bioRxiv*, (2020).
12. M. C. Crank, T. J. Ruckwardt, M. Chen, K. M. Morabito, E. Phung, P. J. Costner, L. A. Holman, S. P. Hickman, N. M. Berkowitz, I. J. Gordon, G. V. Yamshchikov, M. R. Gaudinski, A. Kumar, L. A. Chang, S. M. Moin, J. P. Hill, A. T. DiPiazza, R. M. Schwartz, L. Kueltzo, J. W. Cooper, P. Chen, J. A. Stein, K. Carlton, J. G. Gall, M. C. Nason, P. D. Kwong, G. L. Chen, J. R. Mascola, J. S. McLellan, J. E. Ledgerwood, B. S. Graham; VRC 317 Study Team, A proof of concept for structure-based vaccine design targeting RSV in humans. *Science* **365**, 505–509 (2019). doi:10.1126/science.aav9033 Medline
13. J. Pallesen, N. Wang, K. S. Corbett, D. Wrapp, R. N. Kirchdoerfer, H. L. Turner, C.

A. Cottrell, M. M. Becker, L. Wang, W. Shi, W. P. Kong, E. L. Andres, A. N. Kettenbach, M. R. Denison, J. D. Chappell, B. S. Graham, A. B. Ward, J. S. McLellan, Immunogenicity and structures of a rationally designed prefusion MERS-CoV spike antigen. *Proc. Natl. Acad. Sci. U.S.A.* **114**, E7348–E7357 (2017). doi:10.1073/pnas.1707304114 Medline

14. F. Amanat, F. Krammer, SARS-CoV-2 Vaccines: Status Report. *Immunity* **52**, 583–589 (2020). doi:10.1016/j.immuni.2020.03.007 Medline

15. L. R. Baden, H. M. El Sahly, B. Essink, K. Kotloff, S. Frey, R. Novak, D. Diemert, S. A. Spector, N. Rouphael, C. B. Creech, J. McGettigan, S. Kehtan, N. Segall, J. Solis, A. Brosz, C. Fierro, H. Schwartz, K. Neuzil, L. Corey, P. Gilbert, H. Janes, D. Follmann, M. Marovich, J. Mascola, L. Polakowski, J. Ledgerwood, B. S. Graham, H. Bennett, R. Pajon, C. Knightly, B. Leav, W. Deng, H. Zhou, S. Han, M. Ivarsson, J. Miller, T. Zaks; COVE Study Group, Efficacy and Safety of the mRNA-1273 SARS-CoV-2 Vaccine. *N. Engl. J. Med.* **384**, 403–416 (2021). Medline

16. L. Dai, G. F. Gao, Viral targets for vaccines against COVID-19. *Nat. Rev. Immunol.* **21**, 73–82 (2021). Medline

17. L. Premkumar, B. Segovia-Chumbez, R. Jadi, D. R. Martinez, R. Raut, A. Markmann, C. Cornaby, L. Bartelt, S. Weiss, Y. Park, C. E. Edwards, E. Weimer, E. M. Scherer, N. Rouphael, S. Edupuganti, D. Weiskopf, L. V. Tse, Y. J. Hou, D. Margolis, A. Sette, M. H. Collins, J. Schmitz, R. S. Baric, A. M. de Silva, The receptor binding domain of the viral spike protein is an immunodominant and highly specific target of antibodies in SARS-CoV-2 patients. *Sci. Immunol.* **5**, eabc8413 (2020). doi:10.1126/sciimmunol.abc8413 Medline

18. M. S. Suthar, M. G. Zimmerman, R. C. Kauffman, G. Mantus, S. L. Linderman, W. H. Hudson, A. Vanderheiden, L. Nyhoff, C. W. Davis, O. Adekunle, M. Affer, M. Sherman, S. Reynolds, H. P. Verkerke, D. N. Alter, J. Guarner, J. Bryksin, M. C. Horwath, C. M. Arthur, N. Saakadze, G. H. Smith, S. Edupuganti, E. M. Scherer, K. Hellmeister, A. Cheng, J. A. Morales, A. S. Neish, S. R. Stowell, F. Frank, E. Ortlund, E. J. Anderson, V. D. Menachery, N. Rouphael, A. K. Mehta, D. S. Stephens, R. Ahmed, J. D. Roback, J. Wrammert, Rapid Generation of Neutralizing Antibody Responses in COVID-19 Patients. *Cell Rep Med* **1**, 100040 (2020). doi:10.1016/j.xcrm.2020.100040 Medline

19. W. H. Chen, L. Du, S. M. Chag, C. Ma, N. Tricoche, X. Tao, C. A. Seid, E. M. Hudspeth, S. Lustigman, C. T. Tseng, M. E. Bottazzi, P. J. Hotez, B. Zhan, S. Jiang, Yeast-expressed recombinant protein of the receptor-binding domain in SARS-CoV spike protein with deglycosylated forms as a SARS vaccine candidate. *Hum. Vaccin. Immunother.* **10**, 648–658 (2014). doi:10.4161/hv.27464 Medline

20. W. H. Chen, X. Tao, A. Agrawal, A. Algaissi, B. H. Peng, J. Pollet, U. Strych, M. E. Bottazzi, P. J. Hotez, S. Lustigman, L. Du, S. Jiang, C. K. Tseng, Yeast-Expressed SARS-CoV Recombinant Receptor-Binding Domain (RBD219-N1) Formulated with Alum Induces Protective Immunity and Reduces Immune Enhancement. *bioRxiv*, (2020).

21. S. G. Reed, M. T. Orr, C. B. Fox, Key roles of adjuvants in modern vaccines. *Nat. Med.* **19**, 1597–1608 (2013). doi:10.1038/nm.3409 Medline

22. M. Kwissa, S. P. Kasturi, B. Pulendran, The science of adjuvants. *Expert Rev. Vaccines* **6**, 673–684 (2007). doi:10.1586/14760584.6.5.673 Medline

23. S. P. Kasturi, M. A. U. Rasheed, C. Havenar-Daughton, M. Pham, T. Legere, Z. J. Sher, Y. Kovalenkov, S. Gumber, J. Y. Huang, R. Gottardo, W. Fulp, A. Sato, S. Sawant, S. Stanfield-Oakley, N. Yates, C. LaBranche, S. M. Alam, G. Tomaras, G. Ferrari, D. Montefiori, J. Wrammert, F. Villinger, M. Tomai, J. Vasilakos, C. B. Fox, S. G. Reed, B. F. Haynes, S. Crotty, R. Ahmed, B. Pulendran, 3M-052, a synthetic TLR-7/8 agonist, induces durable HIV-1 envelope-specific plasma cells and humoral immunity in nonhuman primates. *Sci. Immunol.* **5**, eabb1025 (2020). doi:10.1126/sciimmunol.abb1025 Medline

24. H. HogenEsch, D. T. O'Hagan, C. B. Fox, Optimizing the utilization of aluminum adjuvants in vaccines: You might just get what you want. *NPJ Vaccines* **3**, 51 (2018). doi:10.1038/s41541-018-0089-x Medline

25. C. Petitdemange, S. P. Kasturi, P. A. Kozlowski, R. Nabi, C. F. Quarnstrom, P. B. J. Reddy, C. A. Derdeyn, L. M. Spicer, P. Patel, T. Legere, Y. O. Kovalenkov, C. C. Labranche, F. Villinger, M. Tomai, J. Vasilakos, B. Haynes, C. Y. Kang, J. S. Gibbs, J. W. Yewdell, D. Barouch, J. Wrammert, D. Montefiori, E. Hunter, R. R. Amara, D. Masopust, B. Pulendran, Vaccine induction of antibodies and tissue-resident CD8+ T cells enhances protection against mucosal SHIV-infection in young macaques. *JCI Insight* **4**, e126047 (2019). doi:10.1172/jci.insight.126047 Medline

26. P. S. Arunachalam, T. P. Charles, V. Joag, V. S. Bollimpelli, M. K. D. Scott, F. Wimmers, S. L. Burton, C. C. Labranche, C. Petitdemange, S. Gangadhara, T. M. Styles, C. F. Quarnstrom, K. A. Walter, T. J. Ketas, T. Legere, P. B. Jagadeesh Reddy, S. P. Kasturi, A. Tsai, B. Z. Yeung, S. Gupta, M. Tomai, J. Vasilakos, G. M. Shaw, C. Y. Kang, J. P. Moore, S. Subramaniam, P. Khatri, D. Montefiori, P. A. Kozlowski, C. A. Derdeyn, E. Hunter, D. Masopust, R. R. Amara, B. Pulendran, T cell-inducing vaccine durably prevents mucosal SHIV infection even with lower neutralizing antibody titers. *Nat. Med.* **26**, 932–940 (2020). doi:10.1038/s41591-020-0858-8 Medline

27. M. Makoni, South Africa responds to new SARS-CoV-2 variant. *Lancet* **397**, 267 (2021). doi:10.1016/S0140-6736(21)00144-6 Medline

28. W. H. Chen, J. Wei, R. T. Kundu, R. Adhikari, Z. Liu, J. Lee, L. Versteeg, C. Poveda, B. Keegan, M. J. Villar, A. C. de Araujo Leao, J. A. Rivera, P. M. Gillespie, J. Pollet, U. Strych, B. Zhan, P. J. Hotez, M. E. Bottazzi, Genetic modification to design a stable yeast-expressed recombinant SARS-CoV-2 receptor binding domain as a COVID-19 vaccine candidate. *Biochim. Biophys. Acta, Gen. Subj.* **1865**, 129893 (2021). doi:10.1016/j.bbagen.2021.129893 Medline

29. P. J. Hotez, D. B. Corry, U. Strych, M. E. Bottazzi, COVID-19 vaccines: Neutralizing antibodies and the alum advantage. *Nat. Rev. Immunol.* **20**, 399–400 (2020). doi:10.1038/s41577-020-0358-6 Medline

30. T. N. Hoang, M. Pino, A. K. Boddapati, E. G. Viox, C. E. Starke, A. A. Upadhyay, S. Gumber, M. Nekorchuk, K. Busman-Sahay, Z. Strongin, J. L. Harper, G. K. Tharp, K. L. Pellegrini, S. Kirejczyk, K. Zandi, S. Tao, T. R. Horton, E. N. Beagle, E. A. Mahar, M. Y. H. Lee, J. Cohen, S. M. Jean, J. S. Wood, F. Connor-Stroud, R. L. Stammen, O. M. Delmas, S. Wang, K. A. Cooney, M. N. Sayegh, L. Wang, P. D. Filev, D. Weiskopf, G. Silvestri, J. Waggoner, A. Piantadosi, S. P. Kasturi, H. Al-Shakhshir, S. P. Ribeiro, R. P. Sekaly, R. D. Levit, J. D. Estes, T. H. Vanderford, R. F. Schinazi, S. E. Bosinger, M. Paiardini, Baricitinib treatment resolves lower-airway macrophage inflammation and neutrophil recruitment in SARS-CoV-2-infected rhesus macaques. *Cell* **184**, 460–475.e21 (2021). Medline

31. V. Excepients.

32. A. Monie, C. F. Hung, R. Roden, T. C. Wu, Cervarix: A vaccine for the prevention of HPV 16, 18-associated cervical cancer. *Biologics* **2**, 97–105 (2008). Medline

33. N. van Doremalen, T. Lambe, A. Spencer, S. Belij-Rammerstorfer, J. N. Purushotham, J. R. Port, V. A. Avanzato, T. Bushmaker, A. Flaxman, M. Ulaszewska, F. Feldmann, E. R. Allen, H. Sharpe, J. Schulz, M. Holbrook, A. Okumura, K. Meade-White, L. Pérez-Pérez, N. J. Edwards, D. Wright, C. Bissett, C. Gilbride, B. N. Williamson, R. Rosenke, D. Long, A. Ishwarbhai, R. Kailath, L. Rose, S. Morris, C. Powers, J. Lovaglio, P. W. Hanley, D. Scott, G. Saturday, E. de Wit, S. C. Gilbert, V. J. Munster, ChAdOx1 nCoV-19 vaccine prevents SARS-CoV-2 pneumonia in rhesus macaques. *Nature* **586**, 578–582 (2020). doi:10.1038/s41586-020-2608-y Medline

34. J. Yu, L. H. Tostanoski, L. Peter, N. B. Mercado, K. McMahan, S. H. Mahrokhian, J. P. Nkolola, J. Liu, Z. Li, A. Chandrashekar, D. R. Martinez, C. Loos, C. Atyeo, S. Fischinger, J. S. Burke, M. D. Slein, Y. Chen, A. Zuiani, F. J. N. Lelis, M. Travers, S. Habibi, L. Pessaint, A. Van Ry, K. Blade, R. Brown, A. Cook, B. Finneyfrock, A. Dodson, E. Teow, J. Velasco, R. Zahn, F. Wegmann, E. A. Bondzie, G. Dagotto, M. S. Gebre, X. He, C. Jacob-Dolan, M. Kirilova, N. Kordana, Z. Lin, L. F. Maxfield, F. Nampanya, R. Nityanandam, J. D. Ventura, H. Wan, Y. Cai, B. Chen, A. G. Schmidt, D. R. Wesemann, R. S. Baric, G. Alter, H. Andersen, M. G. Lewis, D. H. Barouch, DNA vaccine protection against SARS-CoV-2 in rhesus macaques. *Science* **369**, 806–811 (2020). doi:10.1126/science.abc6284 Medline

35. N. B. Mercado, R. Zahn, F. Wegmann, C. Loos, A. Chandrashekar, J. Yu, J. Liu, L.

Peter, K. McMahan, L. H. Tostanoski, X. He, D. R. Martinez, L. Rutten, R. Bos, D. van Manen, J. Vellinga, J. Custers, J. P. Langedijk, T. Kwaks, M. J. G. Bakkers, D. Zuijdgeest, S. K. Rosendahl Huber, C. Atyeo, S. Fischinger, J. S. Burke, J. Feldman, B. M. Hauser, T. M. Caradonna, E. A. Bondzie, G. Dagotto, M. S. Gebre, E. Hoffman, C. Jacob-Dolan, M. Kirilova, Z. Li, Z. Lin, S. H. Mahrokhian, L. F. Maxfield, F. Nampanya, R. Nityanandam, J. P. Nkolola, S. Patel, J. D. Ventura, K. Verrington, H. Wan, L. Pessaint, A. Van Ry, K. Blade, A. Strasbaugh, M. Cabus, R. Brown, A. Cook, S. Zouantchangadou, E. Teow, H. Andersen, M. G. Lewis, Y. Cai, B. Chen, A. G. Schmidt, R. K. Reeves, R. S. Baric, D. A. Lauffenburger, G. Alter, P. Stoffels, M. Mammen, J. Van Hoof, H. Schuitemaker, D. H. Barouch, Single-shot Ad26 vaccine protects against SARS-CoV-2 in rhesus macaques. *Nature* **586**, 583–588 (2020). doi:10.1038/s41586-020-2607-z Medline

36. K. S. Corbett, B. Flynn, K. E. Foulds, J. R. Francica, S. Boyoglu-Barnum, A. P. Werner, B. Flach, S. O'Connell, K. W. Bock, M. Minai, B. M. Nagata, H. Andersen, D. R. Martinez, A. T. Noe, N. Douek, M. M. Donaldson, N. N. Nji, G. S. Alvarado, D. K. Edwards, D. R. Flebbe, E. Lamb, N. A. Doria-Rose, B. C. Lin, M. K. Louder, S. O'Dell, S. D. Schmidt, E. Phung, L. A. Chang, C. Yap, J. M. Todd, L. Pessaint, A. Van Ry, S. Browne, J. Greenhouse, T. Putman-Taylor, A. Strasbaugh, T. A. Campbell, A. Cook, A. Dodson, K. Steingrebe, W. Shi, Y. Zhang, O. M. Abiona, L. Wang, A. Pegu, E. S. Yang, K. Leung, T. Zhou, I. T. Teng, A. Widge, I. Gordon, L. Novik, R. A. Gillespie, R. J. Loomis, J. I. Moliva, G. Stewart-Jones, S. Himansu, W. P. Kong, M. C. Nason, K. M. Morabito, T. J. Ruckwardt, J. E. Ledgerwood, M. R. Gaudinski, P. D. Kwong, J. R. Mascola, A. Carfi, M. G. Lewis, R. S. Baric, A. McDermott, I. N. Moore, N. J. Sullivan, M. Roederer, R. A. Seder, B. S. Graham, Evaluation of the mRNA-1273 Vaccine against SARS-CoV-2 in Nonhuman Primates. *N. Engl. J. Med.* **383**, 1544–1555 (2020). doi:10.1056/NEJMoa2024671 Medline

37. U. Sahin, A. Muik, E. Derhovanessian, I. Vogler, L. M. Kranz, M. Vormehr, A. Baum, K. Pascal, J. Quandt, D. Maurus, S. Brachtendorf, V. Lörks, J. Sikorski, R. Hilker, D. Becker, A. K. Eller, J. Grützner, C. Boesler, C. Rosenbaum, M. C. Kühnle, U. Luxemburger, A. Kemmer-Brück, D. Langer, M. Bexon, S. Bolte, K. Karikó, T. Palanche, B. Fischer, A. Schultz, P. Y. Shi, C. Fontes-Garfias, J. L. Perez, K. A. Swanson, J. Loschko, I. L. Scully, M. Cutler, W. Kalina, C. A. Kyratsous, D. Cooper, P. R. Dormitzer, K. U. Jansen, Ö. Türeci, COVID-19 vaccine BNT162b1 elicits human antibody and T_H1 T cell responses. *Nature* **586**, 594–599 (2020). doi:10.1038/s41586-020-2814-7 Medline

38. S. P. Kasturi, P. A. Kozlowski, H. I. Nakaya, M. C. Burger, P. Russo, M. Pham, Y. Kovalenkov, E. L. V. Silveira, C. Havenar-Daughton, S. L. Burton, K. M. Kilgore, M. J. Johnson, R. Nabi, T. Legere, Z. J. Sher, X. Chen, R. R. Amara, E. Hunter, S. E. Bosinger, P. Spearman, S. Crotty, F. Villinger, C. A. Derdeyn, J. Wrammert, B. Pulendran, Adjuvanting a Simian Immunodeficiency Virus Vaccine with Toll-Like Receptor Ligands Encapsulated in Nanoparticles Induces Persistent Antibody Responses and Enhanced Protection in TRIM5α Restrictive Macaques. *J. Virol.* **91**, ••• (2017). doi:10.1128/JVI.01844-16 Medline

39. D. Weissman, M. G. Alameh, T. de Silva, P. Collini, H. Hornsby, R. Brown, C. C. LaBranche, R. J. Edwards, L. Sutherland, S. Santra, K. Mansouri, S. Gobeil, C. McDanal, N. Pardi, N. Hengartner, P. J. C. Lin, Y. Tam, P. A. Shaw, M. G. Lewis, C. Boesler, U. Şahin, P. Acharya, B. F. Haynes, B. Korber, D. C. Montefiori, D614G Spike Mutation Increases SARS CoV-2 Susceptibility to Neutralization. *Cell Host Microbe* **29**, 23–31.e4 (2021). doi:10.1016/j.chom.2020.11.012 Medline

40. N. L. Washington, K. Gangavarapu, M. Zeller, A. Bolze, E. T. Cirulli, K. M. Schiabor Barrett, B. B. Larsen, C. Anderson, S. White, T. Cassens, S. Jacobs, G. Levan, J. Nguyen, J. M. Ramirez 3rd, C. Rivera-Garcia, E. Sandoval, X. Wang, D. Wong, E. Spencer, R. Robles-Sikisaka, E. Kurzban, L. D. Hughes, X. Deng, C. Wang, V. Servellita, H. Valentine, P. De Hoff, P. Seaver, S. Sathe, K. Gietzen, B. Sickler, J. Antico, K. Hoon, J. Liu, A. Harding, O. Bakhtar, T. Basler, B. Austin, D. MacCannell, M. Isaksson, P. G. Febbo, D. Becker, M. Laurent, E. McDonald, G. W. Yeo, R. Knight, L. C. Laurent, E. de Feo, M. Worobey, C. Y. Chiu, M. A. Suchard, J. T. Lu, W. Lee, K. G. Andersen, Emergence and rapid transmission of SARS-CoV-2 B.1.1.7 in the United States. *Cell* **184**, 2587–2594.e7 (2021). doi:10.1016/j.cell.2021.03.052 Medline

41. A. W. Boesch, N. Y. Osei-Owusu, A. R. Crowley, T. H. Chu, Y. N. Chan, J. A. Weiner, P. Bharadwaj, R. Hards, M. E. Adamo, S. A. Gerber, S. L. Cocklin, J. E. Schmitz, A. R. Miles, J. W. Eckman, A. J. Belli, K. A. Reimann, M. E. Ackerman, Biophysical and Functional Characterization of Rhesus Macaque IgG Subclasses. *Front. Immunol.* **7**, 589 (2016). doi:10.3389/fimmu.2016.00589 Medline

42. K. McMahan, J. Yu, N. B. Mercado, C. Loos, L. H. Tostanoski, A. Chandrashekar, J. Liu, L. Peter, C. Atyeo, A. Zhu, E. A. Bondzie, G. Dagotto, M. S. Gebre, C. Jacob-Dolan, Z. Li, F. Nampanya, S. Patel, L. Pessaint, A. Van Ry, K. Blade, J. Yalley-Ogunro, M. Cabus, R. Brown, A. Cook, E. Teow, H. Andersen, M. G. Lewis, D. A. Lauffenburger, G. Alter, D. H. Barouch, Correlates of protection against SARS-CoV-2 in rhesus macaques. *Nature* **590**, 630–634 (2021). Medline

43. P. S. Arunachalam, F. Wimmers, C. K. P. Mok, R. A. P. M. Perera, M. Scott, T. Hagan, N. Sigal, Y. Feng, L. Bristow, O. Tak-Yin Tsang, D. Wagh, J. Coller, K. L. Pellegrini, D. Kazmin, G. Alaaeddine, W. S. Leung, J. M. C. Chan, T. S. H. Chik, C. Y. C. Choi, C. Huerta, M. Paine McCullough, H. Lv, E. Anderson, S. Edupuganti, A. A. Upadhyay, S. E. Bosinger, H. T. Maecker, P. Khatri, N. Rouphael, M. Peiris, B. Pulendran, Systems biological assessment of immunity to mild versus severe COVID-19 infection in humans. *Science* **369**, 1210–1220 (2020). doi:10.1126/science.abc6261 Medline

44. S. P. Kasturi, I. Skountzou, R. A. Albrecht, D. Koutsonanos, T. Hua, H. I. Nakaya, R. Ravindran, S. Stewart, M. Alam, M. Kwissa, F. Villinger, N. Murthy, J. Steel, J. Jacob, R. J. Hogan, A. García-Sastre, R. Compans, B. Pulendran, Programming the magnitude and persistence of antibody responses with innate immunity. *Nature* **470**, 543–547 (2011). doi:10.1038/nature09737 Medline

45. E. L. Silveira, S. P. Kasturi, Y. Kovalenkov, A. U. Rasheed, P. Yeiser, Z. S. Jinnah, T. H. Legere, B. Pulendran, F. Villinger, J. Wrammert, Vaccine-induced plasmablast responses in rhesus macaques: Phenotypic characterization and a source for generating antigen-specific monoclonal antibodies. *J. Immunol. Methods* **416**, 69–83 (2015). Medline

46. E. J. Kunkel, E. C. Butcher, Plasma-cell homing. *Nat. Rev. Immunol.* **3**, 822–829 (2003). doi:10.1038/nri1203 Medline

47. A. E. Hauser, G. F. Debes, S. Arce, G. Cassese, A. Hamann, A. Radbruch, R. A. Manz, Chemotactic responsiveness toward ligands for CXCR3 and CXCR4 is regulated on plasma blasts during the time course of a memory immune response. *J. Immunol.* **169**, 1277–1282 (2002). doi:10.4049/jimmunol.169.3.1277 Medline

48. Q. Gao, L. Bao, H. Mao, L. Wang, K. Xu, M. Yang, Y. Li, L. Zhu, N. Wang, Z. Lv, H. Gao, X. Ge, B. Kan, Y. Hu, J. Liu, F. Cai, D. Jiang, Y. Yin, C. Qin, J. Li, X. Gong, X. Lou, W. Shi, D. Wu, H. Zhang, L. Zhu, W. Deng, Y. Li, J. Lu, C. Li, X. Wang, W. Yin, Y. Zhang, C. Qin, Development of an inactivated vaccine candidate for SARS-CoV-2. *Science* **369**, 77–81 (2020). doi:10.1126/science.abc1932 Medline

49. N. B. Mercado, R. Zahn, F. Wegmann, C. Loos, A. Chandrashekar, J. Yu, J. Liu, L. Peter, K. McMahan, L. H. Tostanoski, X. He, D. R. Martinez, L. Rutten, R. Bos, D. van Manen, J. Vellinga, J. Custers, J. P. Langedijk, T. Kwaks, M. J. G. Bakkers, D. Zuijdgeest, S. K. Rosendahl Huber, C. Atyeo, S. Fischinger, J. S. Burke, J. Feldman, B. M. Hauser, T. M. Caradonna, E. A. Bondzie, G. Dagotto, M. S. Gebre, E. Hoffman, C. Jacob-Dolan, M. Kirilova, Z. Li, Z. Lin, S. H. Mahrokhian, L. F. Maxfield, F. Nampanya, R. Nityanandam, J. P. Nkolola, S. Patel, J. D. Ventura, K. Verrington, H. Wan, L. Pessaint, A. Van Ry, K. Blade, A. Strasbaugh, M. Cabus, R. Brown, A. Cook, S. Zouantchangadou, E. Teow, H. Andersen, M. G. Lewis, Y. Cai, B. Chen, A. G. Schmidt, R. K. Reeves, R. S. Baric, D. A. Lauffenburger, G. Alter, P. Stoffels, M. Mammen, J. Van Hoof, H. Schuitemaker, D. H. Barouch, Single-shot Ad26 vaccine protects against SARS-CoV-2 in rhesus macaques. *Nature* **586**, 583–588 (2020). doi:10.1038/s41586-020-2607-z Medline

50. F. P. Polack, S. J. Thomas, N. Kitchin, J. Absalon, A. Gurtman, S. Lockhart, J. L. Perez, G. Pérez Marc, E. D. Moreira, C. Zerbini, R. Bailey, K. A. Swanson, S. Roychoudhury, K. Koury, P. Li, W. V. Kalina, D. Cooper, R. W. Frenck Jr., L. L. Hammitt, Ö. Türeci, H. Nell, A. Schaefer, S. Ünal, D. B. Tresnan, S. Mather, P. R. Dormitzer, U. Şahin, K. U. Jansen, W. C. Gruber; C4591001 Clinical Trial Group, Safety and Efficacy of the BNT162b2 mRNA Covid-19 Vaccine. *N. Engl. J. Med.*

383, 2603–2615 (2020). doi:10.1056/NEJMoa2034577 Medline

51. E. S. Winkler, P. Gilchuk, J. Yu, A. L. Bailey, R. E. Chen, S. J. Zost, H. Jang, Y. Huang, J. D. Allen, J. B. Case, R. E. Sutton, R. H. Carnahan, T. L. Darling, A. C. M. Boon, M. Mack, R. D. Head, T. M. Ross, J. E. Crowe, M. S. Diamond, Human neutralizing antibodies against SARS-CoV-2 require intact Fc effector functions and monocytes for optimal therapeutic protection. *bioRxiv*, (2020).

52. K. O. Saunders, E. Lee, R. Parks, D. R. Martinez, D. Li, H. Chen, R. J. Edwards, S. Gobeil, M. Barr, K. Mansouri, S. M. Alam, L. L. Sutherland, F. Cai, A. M. Sanzone, M. Berry, K. Manne, K. W. Bock, M. Minai, B. M. Nagata, A. B. Kapingidza, M. Azoitei, L. V. Tse, T. D. Scobey, R. L. Spreng, R. W. Rountree, C. T. DeMarco, T. N. Denny, C. W. Woods, E. W. Petzold, J. Tang, T. H. Oguin 3rd, G. D. Sempowski, M. Gagne, D. C. Douek, M. A. Tomai, C. B. Fox, R. Seder, K. Wiehe, D. Weissman, N. Pardi, H. Golding, S. Khurana, P. Acharya, H. Andersen, M. G. Lewis, I. N. Moore, D. C. Montefiori, R. S. Baric, B. F. Haynes, Neutralizing antibody vaccine for pandemic and pre-emergent coronaviruses. *Nature* **594**, 553–559 (2021). doi:10.1038/s41586-021-03594-0 Medline

53. M. Guebre-Xabier, N. Patel, J. H. Tian, B. Zhou, S. Maciejewski, K. Lam, A. D. Portnoff, M. J. Massare, M. B. Frieman, P. A. Piedra, L. Ellingsworth, G. Glenn, G. Smith, NVX-CoV2373 vaccine protects cynomolgus macaque upper and lower airways against SARS-CoV-2 challenge. *Vaccine* **38**, 7892–7896 (2020). doi:10.1016/j.vaccine.2020.10.064 Medline

54. M. Jeyanathan, S. Afkhami, F. Smaill, M. S. Miller, B. D. Lichty, Z. Xing, Immunological considerations for COVID-19 vaccine strategies. *Nat. Rev. Immunol.* **20**, 615–632 (2020). doi:10.1038/s41577-020-00434-6 Medline

55. K. S. Corbett, B. Flynn, K. E. Foulds, J. R. Francica, S. Boyoglu-Barnum, A. P. Werner, B. Flach, S. O'Connell, K. W. Bock, M. Minai, B. M. Nagata, H. Andersen, D. R. Martinez, A. T. Noe, N. Douek, M. M. Donaldson, N. N. Nji, G. S. Alvarado, D. K. Edwards, D. R. Flebbe, E. Lamb, N. A. Doria-Rose, B. C. Lin, M. K. Louder, S. O'Dell, S. D. Schmidt, E. Phung, L. A. Chang, C. Yap, J. M. Todd, L. Pessaint, A. Van Ry, S. Browne, J. Greenhouse, T. Putman-Taylor, A. Strasbaugh, T. A. Campbell, A. Cook, A. Dodson, K. Steingrebe, W. Shi, Y. Zhang, O. M. Abiona, L. Wang, A. Pegu, E. S. Yang, K. Leung, T. Zhou, I. T. Teng, A. Widge, I. Gordon, L. Novik, R. A. Gillespie, R. J. Loomis, J. I. Moliva, G. Stewart-Jones, S. Himansu, W. P. Kong, M. C. Nason, K. M. Morabito, T. J. Ruckwardt, J. E. Ledgerwood, M. R. Gaudinski, P. D. Kwong, J. R. Mascola, A. Carfi, M. G. Lewis, R. S. Baric, A. McDermott, I. N. Moore, N. J. Sullivan, M. Roederer, R. A. Seder, B. S. Graham, Evaluation of the mRNA-1273 Vaccine against SARS-CoV-2 in Nonhuman Primates. *N. Engl. J. Med.* **383**, 1544–1555 (2020). doi:10.1056/NEJMoa2024671 Medline

56. N. S. Wilson, B. Yang, A. B. Morelli, S. Koernig, A. Yang, S. Loeser, D. Airey, L. Provan, P. Hass, H. Braley, S. Couto, D. Drane, J. Boyle, G. T. Belz, A. Ashkenazi, E. Maraskovsky, ISCOMATRIX vaccines mediate CD8+ T-cell cross-priming by a MyD88-dependent signaling pathway. *Immunol. Cell Biol.* **90**, 540–552 (2012). doi:10.1038/icb.2011.71 Medline

57. M. Cabeza-Cabrerizo, A. Cardoso, C. M. Minutti, M. Pereira da Costa, C. Reis E Sousa, Dendritic Cells Revisited. *Annu. Rev. Immunol.* **39**, 131–166 (2021). doi:10.1146/annurev-immunol-061020-053707 Medline

58. E. Segura, A. L. Albiston, I. P. Wicks, S. Y. Chai, J. A. Villadangos, Different cross-presentation pathways in steady-state and inflammatory dendritic cells. *Proc. Natl. Acad. Sci. U.S.A.* **106**, 20377–20381 (2009). doi:10.1073/pnas.0910295106 Medline

59. A. M. Vieira Gomes, T. Souza Carmo, L. Silva Carvalho, F. Mendonça Bahia, N. S. Parachin, Comparison of Yeasts as Hosts for Recombinant Protein Production. *Microorganisms* **6**, 38 (2018). doi:10.3390/microorganisms6020038 Medline

60. S. Burgdorf, V. Lukacs-Kornek, C. Kurts, The mannose receptor mediates uptake of soluble but not of cell-associated antigen for cross-presentation. *J. Immunol.* **176**, 6770–6776 (2006). doi:10.4049/jimmunol.176.11.6770 Medline

61. M. Kwissa, H. I. Nakaya, N. Onlamoon, J. Wrammert, F. Villinger, G. C. Perng, S. Yoksan, K. Pattanapanyasat, K. Chokephaibulkit, R. Ahmed, B. Pulendran, Dengue virus infection induces expansion of a CD14(+)CD16(+) monocyte population that stimulates plasmablast differentiation. *Cell Host Microbe* **16**, 115–127 (2014). doi:10.1016/j.chom.2014.06.001 Medline

62. C. B. Fox, M. T. Orr, N. Van Hoeven, S. C. Parker, T. J. Mikasa, T. Phan, E. A. Beebe, G. I. Nana, S. W. Joshi, M. A. Tomai, J. Elvecrog, T. R. Fouts, S. G. Reed, Adsorption of a synthetic TLR7/8 ligand to aluminum oxyhydroxide for enhanced vaccine adjuvant activity: A formulation approach. *J. Control. Release* **244** (Pt A), 98–107 (2016). doi:10.1016/j.jconrel.2016.11.011 Medline

63. D. Stadlbauer, F. Amanat, V. Chromikova, K. Jiang, S. Strohmeier, G. A. Arunkumar, J. Tan, D. Bhavsar, C. Capuano, E. Kirkpatrick, P. Meade, R. N. Brito, C. Teo, M. McMahon, V. Simon, F. Krammer, SARS-CoV-2 Seroconversion in Humans: A Detailed Protocol for a Serological Assay, Antigen Production, and Test Setup. *Curr. Protoc. Microbiol.* **57**, e100 (2020). doi:10.1002/cpmc.100 Medline

64. A. Vanderheiden, V. V. Edara, K. Floyd, R. C. Kauffman, G. Mantus, E. Anderson, N. Rouphael, S. Edupuganti, P. Y. Shi, V. D. Menachery, J. Wrammert, M. S. Suthar, Development of a Rapid Focus Reduction Neutralization Test Assay for Measuring SARS-CoV-2 Neutralizing Antibodies. *Curr. Protoc. Immunol.* **131**, e116 (2020). doi:10.1002/cpim.116 Medline

65. X. Xie, A. Muruato, K. G. Lokugamage, K. Narayanan, X. Zhang, J. Zou, J. Liu, C. Schindewolf, N. E. Bopp, P. V. Aguilar, K. S. Plante, S. C. Weaver, S. Makino, J. W. LeDuc, V. D. Menachery, P. Y. Shi, An Infectious cDNA Clone of SARS-CoV-2. *Cell Host Microbe* **27**, 841–848.e3 (2020). doi:10.1016/j.chom.2020.04.004 Medline

66. L. C. Katzelnick, A. Coello Escoto, B. D. McElvany, C. Chávez, H. Salje, W. Luo, I. Rodriguez-Barraquer, R. Jarman, A. P. Durbin, S. A. Diehl, D. J. Smith, S. S. Whitehead, D. A. T. Cummings, Viridot: An automated virus plaque (immunofocus) counter for the measurement of serological neutralizing responses with application to dengue virus. *PLOS Negl. Trop. Dis.* **12**, e0006862 (2018). doi:10.1371/journal.pntd.0006862 Medline

67. A. Verma, B. A. Schmidt, S. R. Elizaldi, N. K. Nguyen, K. A. Walter, Z. Beck, H. V. Trinh, A. R. Dinasarapu, Y. S. Lakshmanappa, N. N. Rane, G. R. Matyas, M. Rao, X. Shen, G. D. Tomaras, C. C. LaBranche, K. A. Reimann, D. H. Foehl, J. S. Gach, D. N. Forthal, P. A. Kozlowski, R. R. Amara, S. S. Iyer, Impact of T_h1 CD4 Follicular Helper T Cell Skewing on Antibody Responses to an HIV-1 Vaccine in Rhesus Macaques. *J. Virol.* **94**, ••• (2020). doi:10.1128/JVI.01737-19 Medline

68. G. Ferrari, J. Pollara, D. Kozink, T. Harms, M. Drinker, S. Freel, M. A. Moody, S. M. Alam, G. D. Tomaras, C. Ochsenbauer, J. C. Kappes, G. M. Shaw, J. A. Hoxie, J. E. Robinson, B. F. Haynes, An HIV-1 gp120 envelope human monoclonal antibody that recognizes a C1 conformational epitope mediates potent antibody-dependent cellular cytotoxicity (ADCC) activity and defines a common ADCC epitope in human HIV-1 serum. *J. Virol.* **85**, 7029–7036 (2011). doi:10.1128/JVI.00171-11 Medline

69. J. Pollara, M. Bonsignori, M. A. Moody, P. Liu, S. M. Alam, K. K. Hwang, T. C. Gurley, D. M. Kozink, L. C. Armand, D. J. Marshall, J. F. Whitesides, J. Kaewkungwal, S. Nitayaphan, P. Pitisuttithum, S. Rerks-Ngarm, M. L. Robb, R. J. O'Connell, J. H. Kim, N. L. Michael, D. C. Montefiori, G. D. Tomaras, H. X. Liao, B. F. Haynes, G. Ferrari, HIV-1 vaccine-induced C1 and V2 Env-specific antibodies synergize for increased antiviral activities. *J. Virol.* **88**, 7715–7726 (2014). doi:10.1128/JVI.00156-14 Medline

70. S. P. Perfetto, D. Ambrozak, R. Nguyen, P. K. Chattopadhyay, M. Roederer, Quality assurance for polychromatic flow cytometry using a suite of calibration beads. *Nat. Protoc.* **7**, 2067–2079 (2012). doi:10.1038/nprot.2012.126 Medline

71. G. Alter, J. M. Malenfant, M. Altfeld, CD107a as a functional marker for the identification of natural killer cell activity. *J. Immunol. Methods* **294**, 15–22 (2004). doi:10.1016/j.jim.2004.08.008 Medline

72. C. S. Nelson, T. Huffman, J. A. Jenks, E. Cisneros de la Rosa, G. Xie, N. Vandergrift, R. F. Pass, J. Pollara, S. R. Permar, HCMV glycoprotein B subunit

vaccine efficacy mediated by nonneutralizing antibody effector functions. *Proc. Natl. Acad. Sci. U.S.A.* **115**, 6267–6272 (2018). doi:10.1073/pnas.1800177115 Medline

73. A. D. Davidson, M. K. Williamson, S. Lewis, D. Shoemark, M. W. Carroll, K. J. Heesom, M. Zambon, J. Ellis, P. A. Lewis, J. A. Hiscox, D. A. Matthews, Characterisation of the transcriptome and proteome of SARS-CoV-2 reveals a cell passage induced in-frame deletion of the furin-like cleavage site from the spike glycoprotein. *Genome Med.* **12**, 68 (2020). doi:10.1186/s13073-020-00763-0 Medline

74. J. Harper, N. Huot, L. Micci, G. Tharp, C. King, P. Rascle, N. Shenvi, H. Wang, C. Galardi, A. A. Upadhyay, F. Villinger, J. Lifson, G. Silvestri, K. Easley, B. Jacquelin, S. Bosinger, M. Müller-Trutwin, M. Paiardini, IL-21 and IFNα therapy rescues terminally differentiated NK cells and limits SIV reservoir in ART-treated macaques. *Nat. Commun.* **12**, 2866 (2021). doi:10.1038/s41467-021-23189-7 Medline

75. J. C. Pruessner, C. Kirschbaum, G. Meinlschmid, D. H. Hellhammer, Two formulas for computation of the area under the curve represent measures of total hormone concentration versus time-dependent change. *Psychoneuroendocrinology* **28**, 916–931 (2003). doi:10.1016/S0306-4530(02)00108-7 Medline

76. J. N. Matthews, D. G. Altman, M. J. Campbell, P. Royston, Analysis of serial measurements in medical research. *BMJ* **300**, 230–235 (1990). doi:10.1136/bmj.300.6719.230 Medline

Acknowledgments: We would like to thank all animal staff for assistance on the RM study as well as Dr. Kalpana Patel and Maureen Thompson for guidance with work at the biosafety level 3 for SARS-CoV-2 research at the Yerkes National Primate Research Center at Emory University. We thank Dr. Fred Cassels, Leader, Enteric and Diarrheal Diseases, PATH Center for Vaccine Innovation and Access for his invaluable contribution with study design, assays and manuscript review. We thank Dr. Ravi Hingorani, at BD Biosciences for inputs on multi-parameter flow cytometry. **Funding:** This work was supported by the Yerkes Pilot Research Pilot Program (part of the Yerkes NPRC Base Grant, P51-OD011132) to S.P.K. and additionally funded from intramural funds contributed by Texas Children's Center for Vaccine Development at Baylor College of Medicine (M.E.B and P.H). M.PA is supported by Fast Grants Awards 2144 and 22206 and William and Lula Pitts Foundation. The research reported in this publication is also supported in part by an Emory EVPHA Synergy Fund award (M.S.S.), COVID-Catalyst-I3 Funds from the Woodruff Health Sciences Center (M.S.S), Center for Childhood Infections and Vaccines (M.S.S), Children's Healthcare of Atlanta (M.S.S), Woodruff Health Sciences Center 2020 COVID-19 CURE Award (M.S.S), and by the National Institutes of Health (NIH) through the National Institute for Allergy and Infectious Diseases under the award numbers ORIP/OD P51OD011132 (M.S.S), 3U19AI057266-17S1, HIPC COVID-19 Supplement U19AI090023. The CFAR Immunology/Emory Vaccine Center Flow cytometry core is supported by the NIH grant (P30 A050509). This work has been supported by NIH contract 75N93019C00065 (A.S, D.W). This work has also been supported by NIH/NIAID contract # HHSN272201800004C (D.C.M, C.C.L, G.D.T, H.G and X.S). **Author contributions:** S.P.K, M.E.B, C.B.F, P.H and M.T conceptualized the study; S.P.K, M.E.B, M.Pa, M.Pi, F.C, U.S and P.H designed and were responsible for the overall conduct of the study; W.H.C, J.P, J.W, B.Z, J.L, Z.L produced, purified and characterized the RBD immunogen under the supervision of M.E.B and P.H; M.Pi and T.A processed all samples collected during immunization with assistance from D.D under the supervision of S.P.K and M.Pa; M.Pi and S.P.K handled all post infection tissues samples in an ABSL-3 suite; D.D and S.P.K assayed binding ELISA titers; H.M and C.L.B assayed for pseudovirus neutralizing Ab response under the supervision of D.C.M; X.S and N.E performed the multiplex binding ELISA under the supervision of G.D.T; V.V.E and K.F assayed for FRNT based live SARS-CoV-2 neutralization under the supervision of M.S.S; J.C.M assayed for anti-RBD IgG in nasal, BAL and rectal secretions as well as IgG subclasses in serum under the supervision of P.K; J.P and D.M designed the SECABA for spike expressing cell binding antibody and NK cell degranulation assay under the supervision of G.F; M.Pi, T.A performed T cell ICS assays, ASC assays in blood, LN's and B.M, as well as all flow cytometry assays under the supervision of S.P.K and M.Pa; S.W and T.H.V assayed for viral load in nasal, BAL and throat samples; J.C, R.L.S. S.M.J, J.S.W, F.C.S organized and handled all procedures with the live SARS-CoV-2 challenge in the ABSL-3 at YNPRC; S.G and S.H performed euthanasia post-challenge and assessed lung-pathology; M.B.L, G.P.S performed the multiplex assay with post-challenge plasma for innate biomarkers under the supervision of S.P.R and R.P.S; D.W and A.S provided the S and N peptide megapool (MP) and guidance on the T cell ICS assays; C.B.F and M.T provided adjuvants/formulations and guided preparation of the inoculum; N.S and K.E performed the statistical analyses on challenge outcome and provided input on statistics used in the study; S.P.K, M.Pi and M.Pa were responsible for all data analyses and figure graphs with critical inputs from S.P.R; S.P.K, and M.Pi wrote the manuscript with critical inputs from M.E.B, P.H and M.Pa and suggestions from all co-authors. **Competing interests:** M.E.B, P.H, J.P, U.S, B.Z, J. W, W.H.C, and Z, J. L are developers of a COVID-19 RBD vaccine construct, which was licensed by Baylor College of Medicine to Biological E Ltd., a commercial vaccine manufacturer for scale-up, production, testing and licensure. M.T is an employee of 3M Corporate Research Materials Laboratory and is involved in the development of 3M-052, the TLR-7/8 targeted agonist. C.B.F is a member of the Scientific Advisory Board of MaxHealth Biotechnology LLC. C.B.F is an inventor on patent application (US 2019/0142935) held/submitted by IDRI that covers the formulation of TLR agonists with lipid excipients and aluminum salts. A.S. is currently a consultant for Gritstone, Flow Pharma, Arcturus, Epitogenesis, Oxfordimmunotech, Caprion and Avalia. LJI has filed for patent protection for various aspects of T cell epitope and vaccine design work All other authors declare they have no competing interests. **Data and materials availability:** All data needed to evaluate the conclusions in the paper are present in the paper or the Supplementary Materials. This work is licensed under a Creative Commons Attribution 4.0 International (CC BY 4.0) license, which permits unrestricted use, distribution, and reproduction in any medium, provided the original work is properly cited. To view a copy of this license, visit https://creativecommons.org/licenses/by/4.0/. This license does not apply to figures/photos/artwork or other content included in the article that is credited to a third party; obtain authorization from the rights holder before using such material.

Submitted 3 March 2021
Accepted 9 July 2021
Published First Release 15 July 2021
10.1126/sciimmunol.abh3634

A

Study design

Group	Number of animals	Immunogen (SARS-CoV-2 RBD)	Adjuvant	Route of vaccination
1	5	——	——	——
2	5	RBD monomer	alum	Intramuscular
3	5	RBD monomer	3M-052-alum	Intramuscular

B

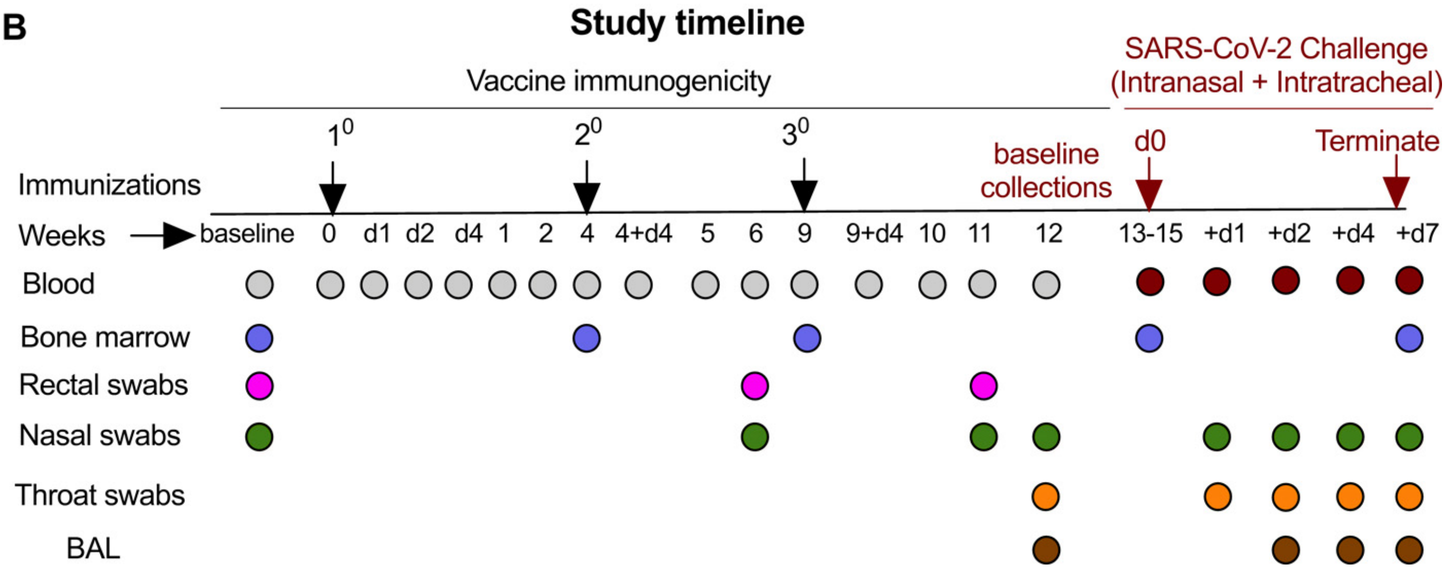

Fig. 1. Study design and timeline: A) Two groups of RMs (n=5 per group) were immunized with a SARS-CoV-2 derived RBD monomer immunogen plus alum alone (group 2) or a combination of 3M-052 and alum (group 3). An additional n = 5 animals included as naïve/unvaccinated controls (group 1) in the study when challenging with SARS-CoV-2. B) Animals in groups 2 and 3 were vaccinated three times at time points indicated in the study timeline. Blood, bone marrow aspirates, nasal and rectal swabs were collected from animals at indicated time points for various assays described in the study. All vaccinated and the naïve control animals were challenged ~ one month post the third vaccination with ~2.3×10^5 PFU of SARS-CoV-2, WA-1 strain via the intranasal and intratracheal routes. Viral loads were quantified in the upper (nasal) and lower respiratory tracts (BAL) as well as in the throat. Animals were sacrificed one week after challenge and anamnestic immune responses if any induced were also quantified post necropsy.

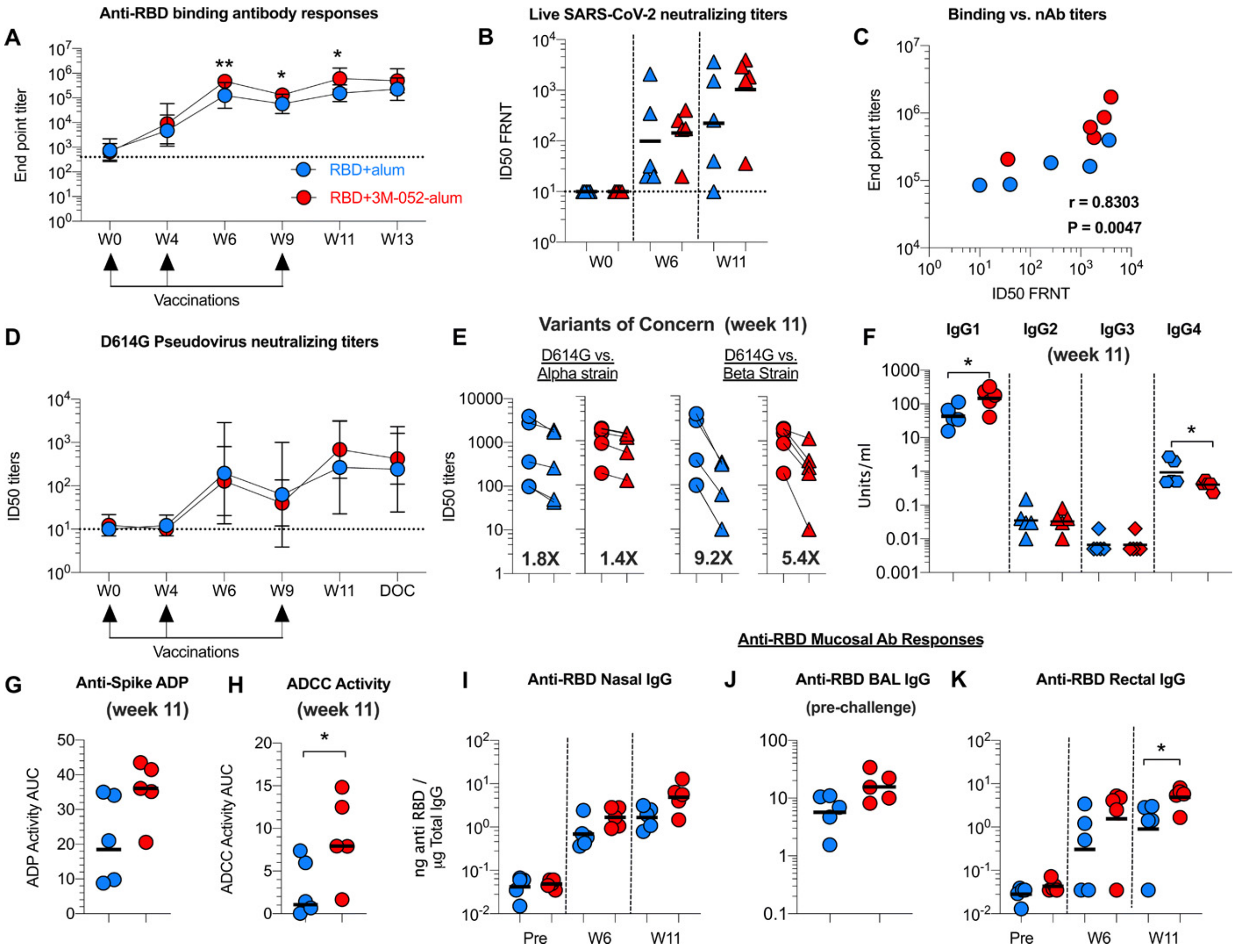

Fig. 2. The RBD+3M-052-alum vaccine induces robust humoral immunity in RMs. A) Line graphs indicate end point titers of anti-RBD immunogen-specific binding Ab responses in RM serum. Geometric mean titers (GMT) with 95% CI are shown (n = 5 per group). The dotted line at 400 indicates the start of serial dilution and a value assigned to animals with no background binding activity. B) Live SARS-CoV-2 neutralization activity using a FRNT assay as detailed in the materials and methods. Horizontal bars indicate GMT. C) The correlation between endpoint titers and live SARS-CoV-2 neutralization activity. D) Line graphs indicate pseudovirus neutralization titers. All values below the limit of detection were assigned a value of 10 for plotting. Geometric mean titers (GMT) with 95% CI are shown (n=5 per group). E) Scatter plots indicate pseudovirus neutralization titers against the SARS-CoV-2 (WA-1) compared with the Alpha and Beta variants of concern (VOCs) respectively. Bold numbers indicate fold change in GMT. F) Scatter plots indicates anti-RBD Ab IgG isotypes assayed at week 11. G) Scatter plots show antibody dependent phagocytic (ADP) activity in serum at week 11. H) Scatter plots indicate antibody-dependent NK cell degranulation activity against target cells when expressing protein with the mutated G614. I) Scatter plots indicate anti-RBD immunogen-specific binding Ab activity in nasal swabs. J) Scatter plots show anti-RBD specific binding Ab activity in BAL. Pre-challenge refers to 5 days prior to SARS-CoV-2 challenge. K) Graph indicates anti-RBD immunogen-specific binding Ab activity in rectal swabs. Horizontal lines in graphs (F-K) show the Geometric mean. Repeated measures analyses were used to test for significant statistical differences in antibody titers and pseudo neutralization titers (A, D) measured longitudinally over time as detailed in methods. P = 0.003 at W6, 0.02 at W9 and 0.002 at W11 in panel A. The difference in magnitude of Ab response in B, F-K was tested using a non-parametric two-tailed Mann-Whitney test using the GraphPad Prism software version 8.0. A value of $P<0.05$ was considered significant. P = 0.0317 for IgG1 and P =0.0476 for IgG4 in panel F. P = 0.0317 in panel H and K. Spearman's correlation analysis was used to compare the correlation between the magnitude of immune responses in C). Spearman's r and p values are indicated on the graphs.

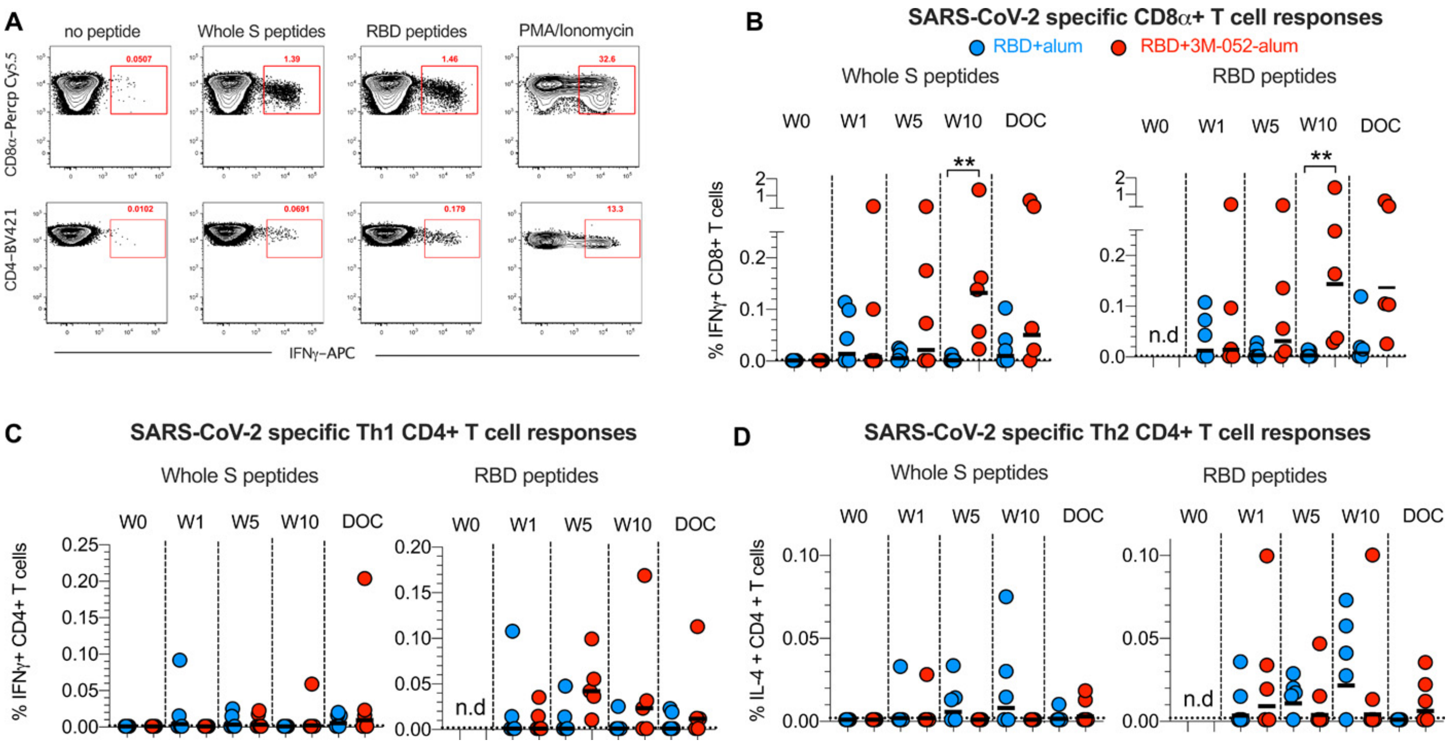

Fig. 3. The RBD+3M-052-alum vaccine in comparison with RBD+alum induces significantly higher RBD-specific CD8+ T cells and a Th1 biased CD4+ T cell response. A) Representative flow cytometry plots show IFN-γ secreting CD8+ and CD4+ T cell responses to whole S megapool and RBD specific peptide pools stimulations ex-vivo, as well as no peptide and PMA + Ionomycin stimulated negative and positive assay controls respectively. Scatter plots summarize frequencies of B) IFN-γ + CD8+ T cells, C) IFN-γ + CD4+ T cells and D) IL-4 + CD4+ T cells when stimulating with whole S megapool and RBD peptide pools. Day of challenge = DOC. Horizontal bars represent the geometric mean. A two tailed Mann-Whitney test was used to compare the significance of differences between groups 2 and 3. ** represents a P value = 0.0079 in panel B.

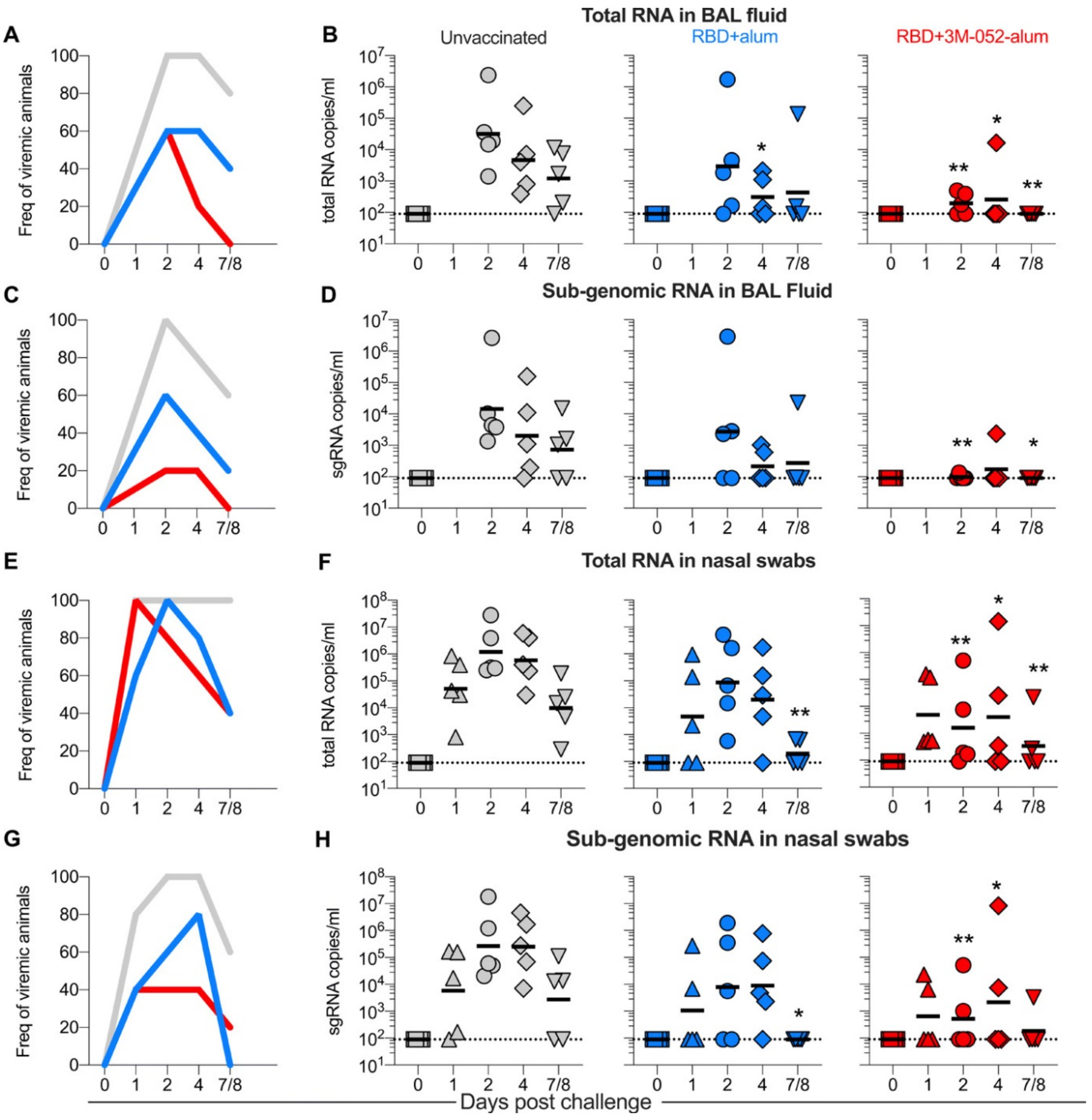

Fig. 4. The RBD+3M-052-alum vaccine significantly reduces total and replicating SARS-CoV-2 in BAL and nasal swabs of RMs post respiratory challenge. A) Line graph indicates frequencies of animals testing positive for total RNA in BAL post challenge. B) Scatter plots indicate total SARS-CoV-2 RNA levels measured in BAL post challenge in all treatment groups. C) Line graph indicates frequencies of animals testing positive for sub-genomic (sgRNA) in BAL post challenge. D) Scatter plots indicate sgRNA levels measured in BAL post challenge. E) Line graph indicates frequencies of animals testing positive for total RNA in nasal swabs post challenge. F) Scatter plots indicate total SARS-CoV-2 RNA levels measured in nasal swabs post challenge. G) Line graph indicates frequencies of animals testing positive for sgRNA in nasal swabs post challenge. H) Scatter plots indicate sgRNA levels measured in nasal swabs post challenge. Horizontal lines in graphs B,D,F,H represent Geometric means. Repeated measures analyses was performed on data with viral loads to compare differences over time between the study groups as detailed in methods. P = 0.029 for group 2 vs 1 in panel B and P <0.001, 0.045 and 0.006 for group 3 vs. 1 at days 2,4 and 7/8 in Panel B. P=<0.001 and 0.024 at days 2 and 7/8 in panel D. In panel F, P=<0.001 for group 2 vs.1 at day 7/8, and P<0.001, 0.032 and 0.017 at days 2,4 and 7/8 for group 3 vs. 1. In panel H, P = 0.012 for group 2 vs. 1 at day 7/8 and P<0.001 and 0.041 at days 2 and 4 for group 3 vs. 1. For overall VL AUC measurements (see materials and methods), P = 0.003 for group 3 vs. 1 and 0.175 for group 2 vs.1 in panel B. P = 0.012 for group 3 vs. 1 and 0.282 for group 2 vs. 1 in panel D. In panel F, P = 0.01 for group 3 vs. 1 and P = 0.006 for group 2 vs.1. Finally, P = 0.014 for group 3 vs. 1 and 0.018 for group 2 vs. 1 in Panel H..

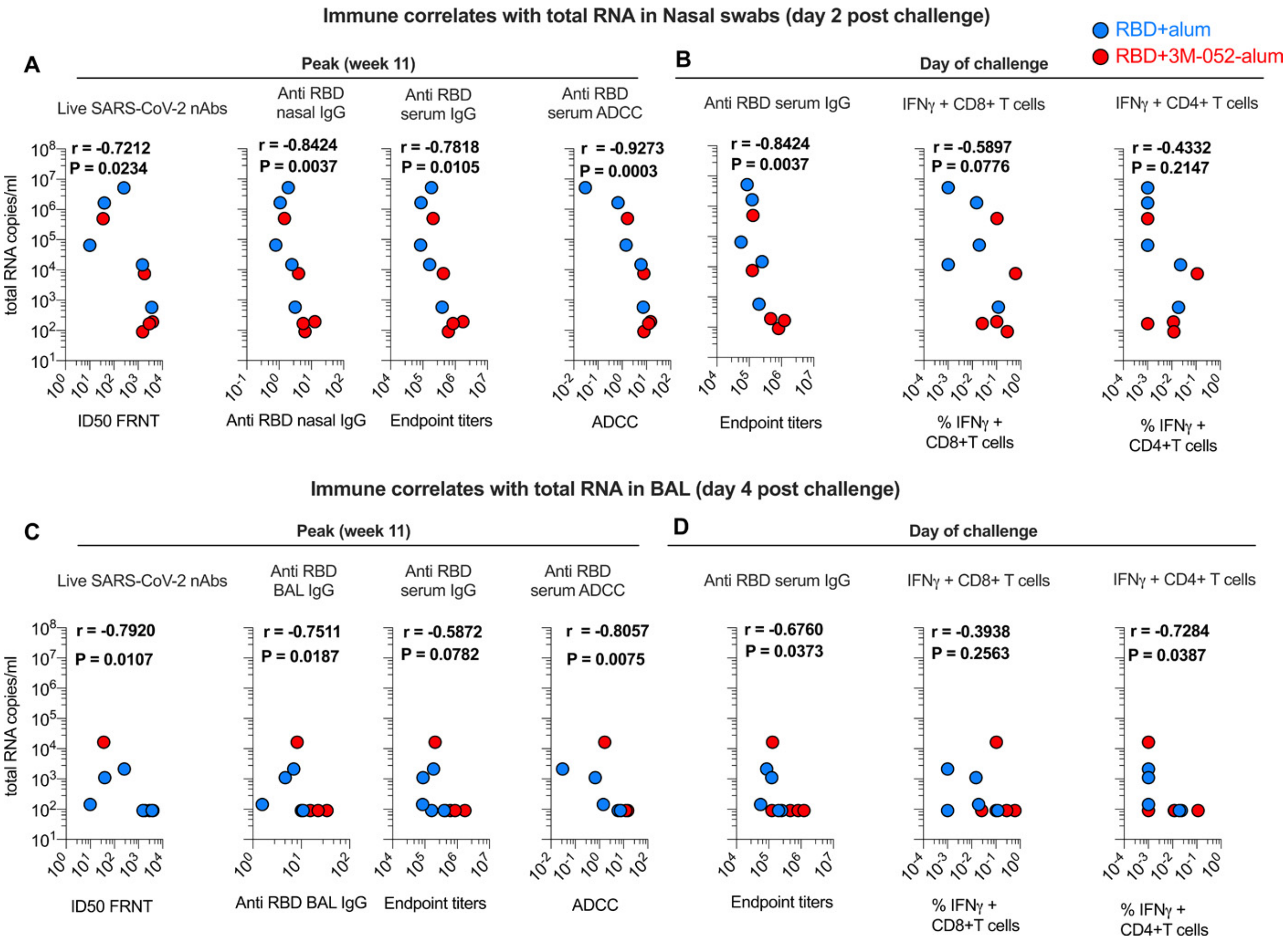

Fig. 5. Anti-RBD Ab responses correlate with total viral RNA in URT and LRT. Correlations of anti-RBD Ab and T cell responses with viral load in URT and LRT were evaluated at both peak and the day of challenge (DOC). A) and B) highlight correlations between Ab and T cell responses induced by vaccines in the study with total SARS-CoV-2 viral RNA in nasal swabs. C) and D) highlight correlations between Ab and T cell responses induced by vaccination with total SARS-CoV-2 viral RNA in BAL. Spearman's correlation was used to identify significance. P and r-values of the Spearman's correlation test are indicated.

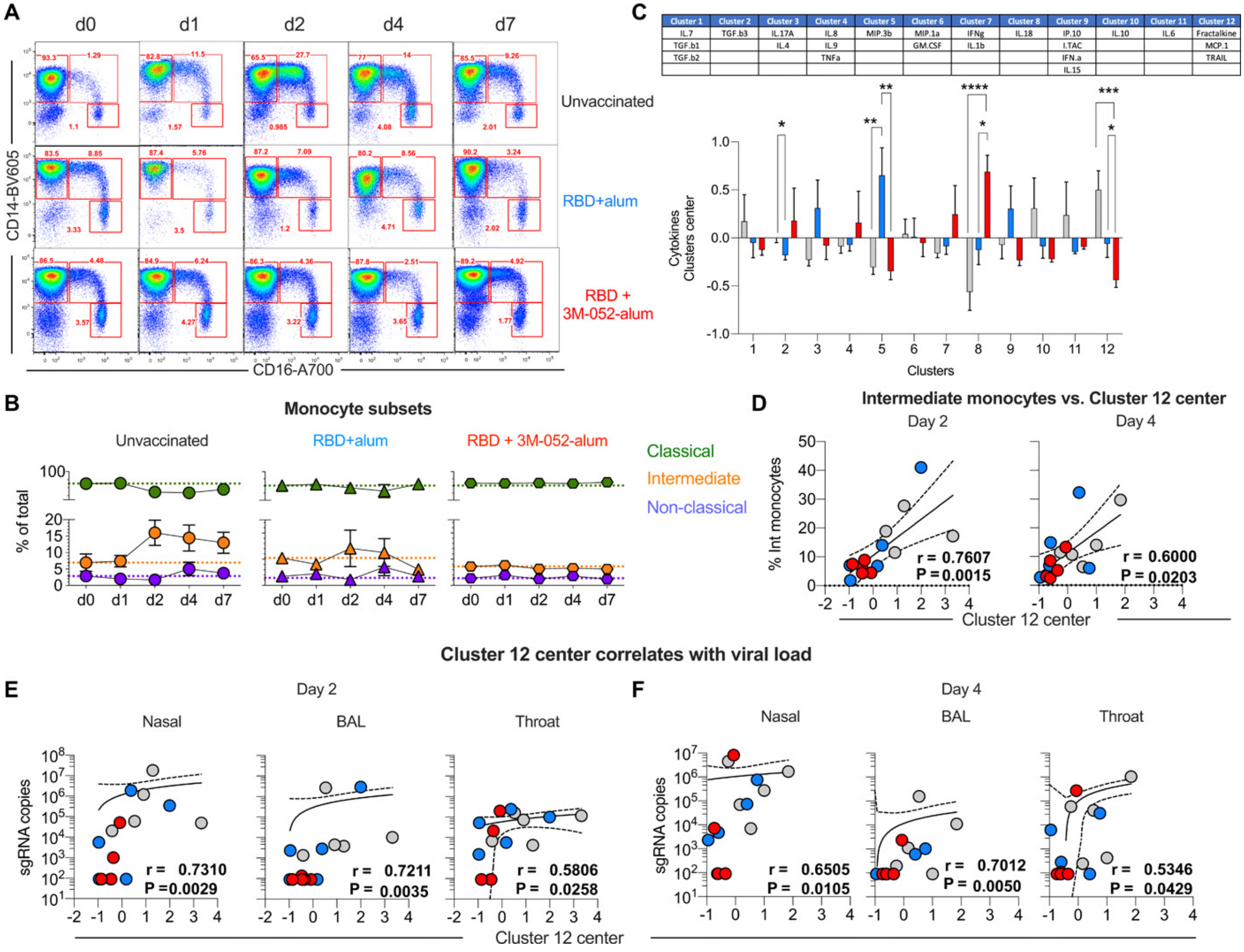

Cluster 1	Cluster 2	Cluster 3	Cluster 4	Cluster 5	Cluster 6	Cluster 7	Cluster 8	Cluster 9	Cluster 10	Cluster 11	Cluster 12
IL.7	TGF.b3	IL.17A	IL.8	MIP.3b	MIP.1a	IFNg	IL.18	IP.10	IL.10	IL.6	Fractalkine
TGF.b1		IL.4	IL.9		GM.CSF	IL.1b		I.TAC			MCP.1
TGF.b2			TNFa					IFN.a			TRAIL
								IL.15			

Fig. 6. Intermediate (CD14+CD16++) monocytes in blood and a cluster of chemokines strongly correlate with viral load upon SARS-CoV-2 challenge in RMs. We have highlighted a gating strategy to identify monocyte subsets in fig. S7. A) Representative flow plots show changes in classical (CM), intermediate (IM) and non-classical (NCM) monocytes at all days post respiratory SARS-CoV-2 challenge in unvaccinated and vaccinated RMs. B) Line graphs summarize proportional changes in monocyte subsets in all n = 5 animals per treatment group. Mean and standard error (SEM) are reported for all time points sampled. C) Plasma soluble factor components in each cluster (upper table); the x and y axes represent clusters and contributions to each treatment group. Bars for unvaccinated (controls) are presented in light grey, in blue for RBD + alum and in red for RBD+alum+3M-052. Error bars indicate standard errors of the means (SEM). Asterisks *, ** and ***, ****, indicate; P value = 0.04 for cluster 2, P= 0.004 for group 2 vs. 1 and 0.0034 for group 3 vs. 2 for cluster 5, P <0.0001 for group 3 vs. 1 and 0.01 for group 3 vs. 2 for cluster 8, and P =0.003 group 3 vs. 1 and 0.049 for group 3 vs. 2 with cluster 12. D) Correlation of the Cluster 12 center with the frequencies of IM's at days 2 and 4 (peak), E) Correlation of the cluster 12 center with sgRNA in nasal and throat swabs as well as BAL at day 2 and F) at day 4. Spearman's correlation was used to identify significance. P and r-values of the Spearman's correlation test are indicated.

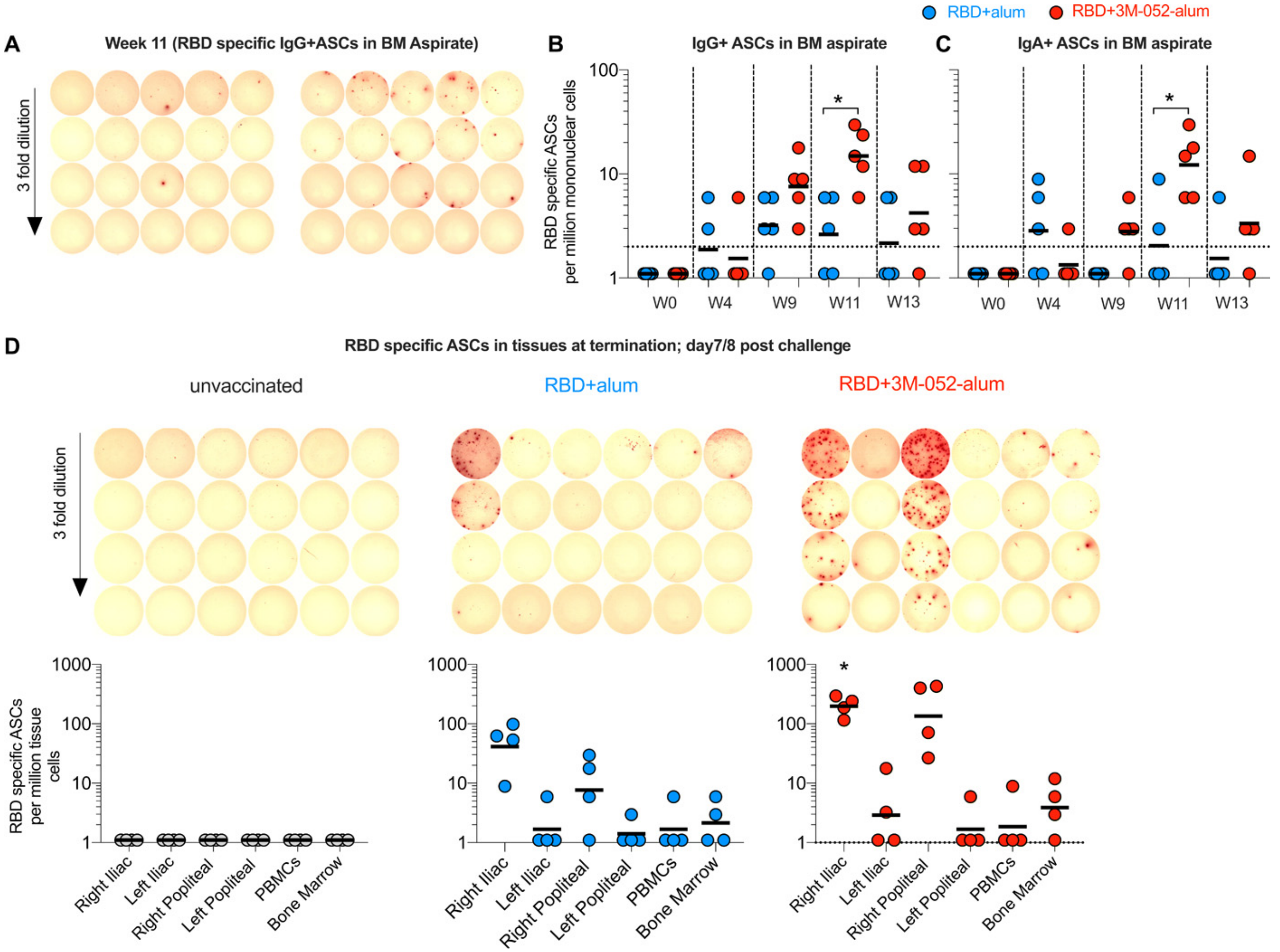

Fig. 7. The RBD+3M-052-alum vaccine in comparison with RBD+alum induces significantly higher RBD specific ASCs in draining LNs. RBD-specific ASCs were enumerated using an ELISpot assay. A) Scanned ELISpot plate images of RBD-specific ASCs at week 11 assayed in BM aspirate are shown. B) Scatter plots summarize frequencies of IgG secreting RBD-specific ASCs in BM aspirates collected before and after vaccination up to a week prior to challenge. C) Scatter plots summarize frequencies of IgA secreting RBD-specific ASCs in BM aspirates. D) Scanned ELISPOT plate images of RBD-specific ASCs in draining and non-draining Iliac and popliteal LNs, PBMCs and BM long bone (femur) scoop tissue at necropsy post challenge is shown for one representative animal in each treatment group. Scatter plots below the scanned images are aligned to indicate the tissue in each column of the ELISpot plate. Data reported here summarizes frequencies of IgG secreting ASCs in LNs, PBMCs and BM of n = 4 animals per treatment group. The significance of the difference in the frequencies of RBD-specific ASCs in tissues was established using a two-tailed Mann-Whitney test. Values are P = 0.023 in panel B, 0.032 in panel C and 0.0238 in panel D when comparing group 3 vs. 2.

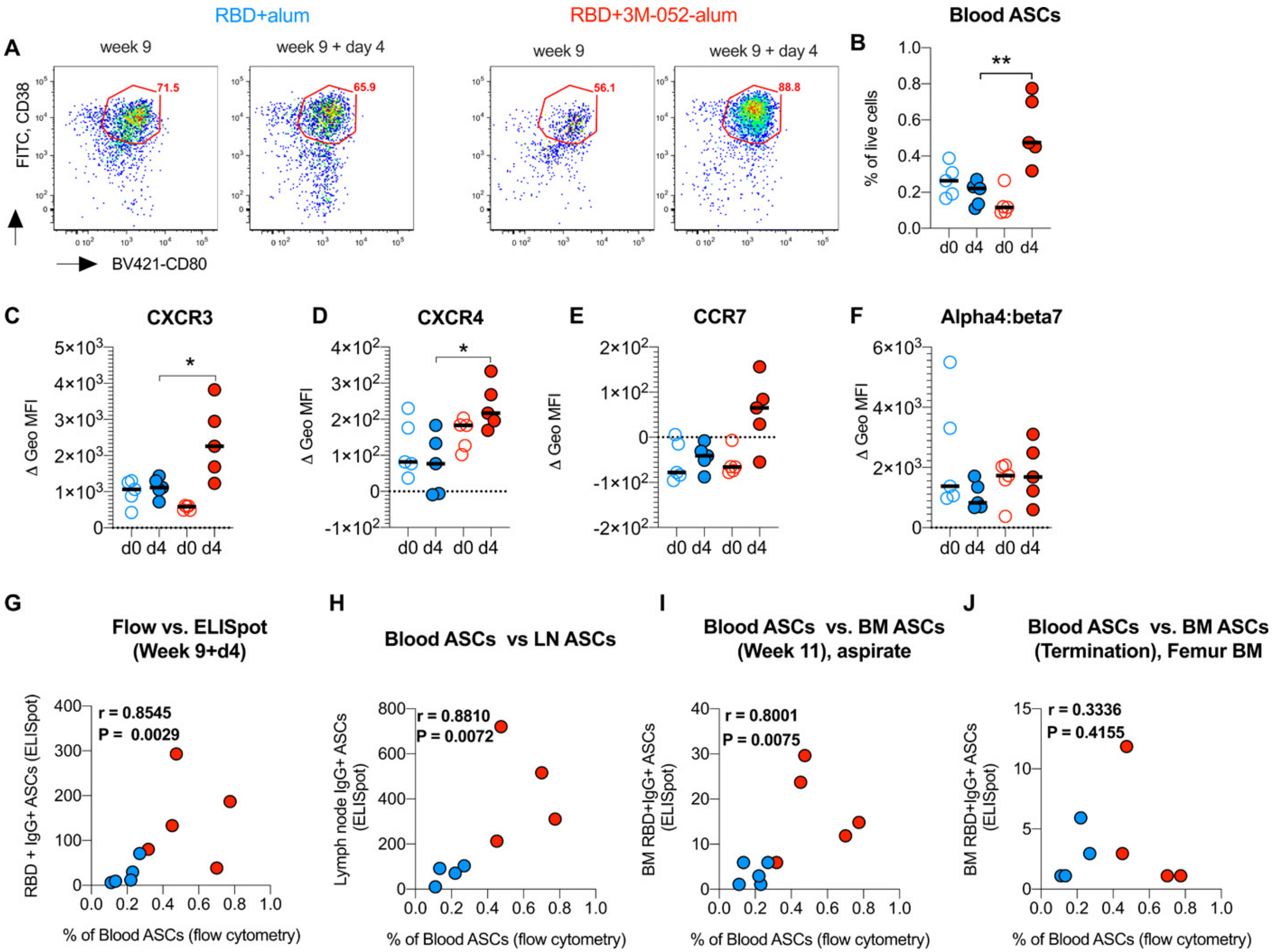

Fig. 8. The RBD+3M-052-alum vaccine in comparison with RBD+alum induces increased frequencies of blood ASCs and up-regulation of tissue homing markers. A) Flow plots highlight CD38+CD80+ASCs (gating strategy in Fig. S12) from one animal each vaccinated with RBD+alum or RBD+3M-052-alum adjuvants at week 9 and 9+day 4. B) Scatter plots summarize frequencies of blood ASCs pre and post vaccination at week 9. Scatter plots in C, D, E and F summarize the change in differential Geometric mean fluorescence intensity (stain – isotype) of chemokine receptors CXCR3, CXCR4, CCR7 and alpha4:beta7 on blood ASCs, before and day 4 post vaccination at week 9 in the study. G) Correlation of flow-based frequencies of total blood ASCs with RBD-specific ELISpot based IgG+ ASCs at day week 9+day 4 in the study is shown. H) Correlation of RBD-specific IgG+ blood ASCs (week 9+day 4) with IgG+ ASCs in draining LNs at termination post challenge is shown. I) Correlation of RBD-specific IgG+ blood ASCs (week 9+day 4) with IgG+ ASCs in BM aspirates at week 11 is shown. J) Correlation of RBD specific IgG+ blood ASCs (week 9+day 4) with IgG+ ASCs in femur BM scoop tissue at necropsy is shown. Statistical significance of the difference in responses measured was tested using a non-parametric two-tailed Mann-Whitney test. A value of $p<0.05$ was used as significant. Values are $P = 0.0079$ in panel B, 0.03 in C, 0.0159 in D.
