# Supplementary material for: A yeast-expressed RBD-based SARS-CoV-2 vaccine formulated with 3M-052-alum adjuvant promotes protective efficacy in non-human primates
Source: Sci Immunol. 2021 Jul 15;6(61):eabh3634. doi: 10.1126/sciimmunol.abh3634 (PMC9119307; doi:10.1126/sciimmunol.abh3634)
Supplement: Supplementary file 2 — Materials and Methods Figs. S1 to S18 [file sciimmunol.abh3634_sm.pdf]

Science Immunology
AAAS

Supplementary Materials for

A yeast-expressed RBD-based SARS-CoV-2 vaccine formulated with 3M-052-alum adjuvant promotes protective efficacy in non-human primates

Maria Pino *et al.*

Corresponding author: Sudhir Pai Kasturi, skastur@emory.edu; Maria Elena Bottazzi, bottazzi@bcm.edu; Mirko Paiardini, mirko.paiardini@emory.edu

Sci. Immunol. **6**, eabh3634 (2021)
DOI: 10.1126/sciimmunol.abh3634

The PDF file includes:

Materials and Methods
Figs. S1 to S18

Other Supplementary Material for this manuscript includes the following:

Tables S1 to S4

Materials and Methods:

Cloning, expression and characterization of SARS-CoV-2 RBD in the yeast *Pichia pastoris*

The DNA encoding RBD219-WT (residues 331–549 of the SARS-CoV-2 spike protein, GenBank: QHD43416.1) was codon-optimized based on yeast codon usage and synthesized by GenScript (Piscataway, NJ, USA), followed by subcloning into the *Pichia* secretory expression vector pPICZαA (Invitrogen) using EcoRI and XbaI restriction sites. The recombinant plasmid DNA was then transformed into *Pichia pastoris* X-33 by electroporation. The expression of the recombinant RBD was confirmed by induction with 0.5% methanol at 30 °C for 72 hours. The purified protein (Lot: S2RBD300520JXL-1) was stored in 20 mM Tris, 150 mM NaCl, pH 7.5 until further use. SARS-CoV-2 RBD was treated with PNGase-F (NEB, P0704S) to remove the N-glycans following the vendor's manual. 6μg of SARS-CoV-2 RBD before and after PNGase-F treatment were loaded on a 4-20% Tris-glycine gel under reduced conditions to evaluate the size. The purity of the SARS-CoV-2 RBD was further evaluated using SE-HPLC. Briefly, 50μg of RBD were injected into a Yarra SEC-3000 column (300mm X 7.8mm; catalog # 00H-4513-K0), and the protein was eluted in 20 mM Tris, 150 mM NaCl, pH 7.5 at a flow rate of 0.6 ml/min.

Analyses of anti-RBD IgG binding Ab responses

RBD was coated on Nunc MaxiSorp plates at a concentration of 2μg/mL in 100μL of phosphate buffered saline (PBS) at 4°C overnight. Plates were blocked for two hours at R.T using 200μL of 4% (w/v) non-fat dry milk (NFDM) (BioRaD). Serum stored at -80°C was thawed at 4° C overnight and diluted in 1% (w/v) NFDM in PBS/0.05%Tween (PBS-T) [ELISA Buffer] starting at 1:400 and serially diluted 1:4 (8x). 100μL of each dilution was added in duplicates and incubated for 2 hours at R.T. Plates were washed 6x with 200μL PBS-T. 100μL of anti-monkey

IgG conjugated with horse radish peroxidase (HRP), (clone-SB108A, Southern Biotechnology Associates, AL) diluted at 1:5000 in ELISA buffer was added and plates were incubated for 1 hour at R.T. Plates were washed again 6x with PBS-T. Tetramethyl benzidine (TMB) substrate was added for 10 minutes at R.T for developing ELISA plates and the reaction was blocked using 2N sulfuric acid. Absorbance was measured at 450nm using a SpectraMax plate reader (Molecular Devices, CA). A cutoff of optical density at 0.1 (also > mean +10S.D of neg wells) was used for computing end point titers using an asymetric 5PL curve fit function in GraphPad Prism version 8.0. A titer of 400, the starting dilution was assigned for baseline samples when no background was detected and any background higher than 400 at baseline if detected, was subtracted for all post-vaccination time points titer calculation.

Live SARS-CoV-2 neutralizing or focus reduction neutralization titer (FRNT) assay:
Serum was serially diluted (three-fold) in serum-free Dulbecco's modified Eagle's medium (DMEM) in duplicate wells and incubated with 100–200 FFU infectious clone derived SARS-CoV-2-mNG (*64*) virus at 37 °C for 1 h. The antibody-virus mixture was added to VeroE6 cell (C1008, ATCC, #CRL-1586) monolayers seeded in 96-well blackout plates and incubated at 37 °C for 1 h. Post-incubation, the inoculum was removed and replaced with pre-warmed complete DMEM containing 0.85% methylcellulose. Plates were incubated at 37 °C for 24 h. After 24 h, methylcellulose overlay was removed, cells were washed twice with PBS and fixed with 2% paraformaldehyde in PBS for 30 min at room temperature. Following fixation, plates were washed twice with PBS and foci were visualized on a fluorescence ELISPOT reader (CTL ImmunoSpot S6 Universal Analyzer) and enumerated using Viridot (*66*). The neutralization titers were calculated as follows: 1 - (ratio of the mean number of foci in the presence of sera and foci at the highest dilution of respective sera sample). Each specimen was tested in two independent assays performed at different times. The FRNT-mNG$_{50}$ titers were

interpolated using a 4-parameter nonlinear regression in GraphPad Prism 8.4.3. Samples with an FRNT-mNG_{50} value that was below the limit of detection were plotted at 10. For these samples, this value was used in fold reduction calculations.

Spike protein-expressing cell antibody binding assay (SECABA)

We used two plasmids expressing the D614 and G614 sequences to generate the 293-F transfected cells. Cells non-transfected with any plasmid (mock-transfected) were used as a negative control condition. After resuspension, washing and counting, $1x10^5$ S-protein transfected target cells were dispensed into 96-well V-bottom plates and incubated with 8 serial dilutions of serum from vaccinated RMs starting at 1:50 dilution. After 30 min incubation at 37°C, cells were washed twice with 250 µL/well of PBS, stained with vital dye (Live/Dead Fixable Aqua Dead Cell Stain, Invitrogen) to exclude nonviable cells from subsequent analysis, washed with Wash Buffer (1%FBS-PBS; WB), permeabilized with CytoFix/CytoPerm (BD Biosciences), and stained with 1.25 µg/mL anti-Rhesus IgG (H+L) -APC (Cat no. 6200-31; Southern Biotech) and 5 µg/mL anti-flag – FITC (clone M2; Sigma Aldrich) in the dark for 20 min at RT. After three washes with Perm Wash (BD Biosciences), the cells were resuspended in 125µL PBS-1% paraformaldehyde. Samples were acquired within 24 h using a BD Fortessa cytometer and a High Throughput Sampler (HTS, BD Biosciences). The cytometers are rigorously maintained under quality control procedures regularly performed as described by Perfetto et al(70). The signal for each fluorophore was detected using: 1) 488 nm/50 mW laser and 530/30 filter for FITC; 2) 405 nm/50 mW laser and 525/50 filter for Live/Dead Aqua; 3) 640 nm/40 mW laser and 670/30 filter for the anti-Rhesus IgG (H+L)-APC. The appropriate compensation beads were used to compensate for the spill over signal for the three fluorophores. Data analysis was performed using FlowJo 10 software (BD Biosciences). A minimum of 50,000 total events were acquired for each analysis. Gates were set to include singlet, live and flag+ events. Anti-Rhesus IgG was gated on baseline samples for each dilution of plasma and applied to

vaccine/challenge samples to calculate %IgG+ cells. For the samples collected from the animals utilized as control, baseline samples were not available; therefore, the position of the gates was determined using the conditions of the plasma binding to the mock-transfected cells. MFI from wells that included the secondary antibody alone (no plasma) were subtracted from samples to calculate the MFI specifically due to mAb/plasma binding.

Antibody-dependent NK cell degranulation assay.

293T target cells were used 2-days post transfection with a plasmid expressing SARS-CoV-2 S protein G614 variant. Serum samples were tested at the 1:100, 1:500, and 1:1,000 dilutions. Samples from a SARS-Cov-2 infected (PC020v1) and a non-infected (SORF Neg) human subject were tested at the same dilutions as positive and negative controls, respectively. NK cells were purified from peripheral blood of a healthy human volunteer by negative selection (Miltenyi Biotech), and were incubated with target cells at a 1:1 ratio in the presence of diluted serum, Brefeldin A (GolgiPlug, 1µl/ml, BD Biosciences), Monensin (GolgiStop, 4µl/6mL, BD Biosciences), and CD107a-FITC (BD Biosciences, clone H4A3) in 96-well flat bottom plates for 6 hours at 37ºC in a humidified 5% CO2 incubator. NK cells were then recovered and stained for viability prior to staining with CD56-PECy7 (BD Biosciences, clone NCAM16.2), CD16-PacBlue (BD Biosciences, clone 3G8), and CD69-BV785 (BioLegend, Clone FN50). Flow cytometry data analysis was performed using FlowJo software (v9.9.6). Data is reported as the % of CD107a+ live NK cells (singlets, lymphocytes, aqua blue-, CD56+ and/or CD16+, CD107a+). All final data were reported as specific activity for each dilution, determined by subtraction of non-specific activity observed in assays performed with mock-infected cells and in absence of Abs. The results were analyzed as area under the curve (AUC) calculated using non-linear trapezoidal rule.

ELISpot assays to quantify ASCs in blood, LN and BM:

Briefly, 96 well multi-screen HTS filter plates (Millipore; MSHAN4B50) were coated overnight at 4°C with 10μg/mL of anti-monkey IgG, IgA, or IgM (H&L) goat antibody (Rockland) or with 4μg/mL of recombinant RBD protein for enumeration of total or antigen-specific antibody secreting cells (ASCs) respectively. Wells were washed 4 times with PBS 0.05% Tween 20 (PBS-T) and 4 times with PBS, and blocked with complete RPMI medium with L-glutamine (supplemented with 10% FBS, 10mM HEPES, 100mM sodium pyruvate, non-essential amino acids, 2-mercaptoethanol and Penicillin/Streptomycin with fungicide) for 2 hours in a 5% CO_2 incubator at 37°C. Whole PBMC, lymph node or bone marrow preparations were diluted in complete RPMI medium, plated in serial 3-fold dilutions starting with 5 million cells/ml and incubated overnight in a 5% CO_2 incubator at 37°C. Wells were washed 4 times with PBS and 4 times with PBS-T, followed by incubation with either anti-monkey IgG, or IgA, -biotin conjugated antibodies (Rockland), diluted 1:1000 in PBS-0.05% Tween 20 1% FBS solution (PBS-T-F), for 2 hours at room temperature. Wells were again washed 4 times with PBS-T before adding Avidin D-HRP (Vector labs) diluted 1:1000 in PBS-T-F. After a 2-3 hour incubation at room temperature, wells were washed 4 times with PBS-T and 4 times with PBS. Spots were developed with filtered 3-amino 9-ethylcarbazole (AEC) substrate (0.3mg/mL AEC diluted in 0.1M of sodium acetate buffer (pH 5.0), containing a 1:1000 dilution of 3% hydrogen peroxide). To stop the reaction, wells were washed with water. Spots were documented and counted using the Immunospot CTL counter and Image Acquisition 4.5 software (Cellular Technology). Once counted, the number of spots specific for each immunoglobulin isotype was reported as the number of either total or antigen-specific ASCs per million PBMCs.

T cell stimulation and intracellular cytokine staining assays:

E-fluor 780 Viability dye (E-bioscience/Thermofisher) was used to stain for dead cells as described before. The following antibodies known to cross-react with RM cells were used in staining cells as described before(*23*). BV421,anti-human CD4 (Clone; OKT4) and PerCP Cy5.5, anti-human CD8a (Clone; RPA-T8) antibodies from Biolegend were used in surface staining. BV655 anti-human CD3 (Clone; SP34-2, BD Biosciences), Alexa-488 anti human IL-2 (Clone; MQ1-17H12) and Alexa-647 anti human IFN-γ(Clone; 4S.B3) from Biolegend, PE E-fluor610 anti human IL-17A(Clone; eBio64DEC17) and PE-Cy7 anti human TNF (Clone; Mab11) from Thermofisher and PE anti human IL-4 (Clone; 7A3-3) was procured from Miltenyi Biotec. Stained cells were acquired using a Fortessa Flow Cytometer (BD Biosciences, CA). Flow cytometry data was analyzed using Flow Jo (TreeStar, Or).

PBMC staining by flow cytometry post challenge:

The following antibodies were used in cell surface staining. Antibodies from Biolegend; Alexa-488 anti human CD40 (Clone; 5C3), Alexa-488, mouse IgG1 k isotype control, PerCP anti human HLA-DR (Clone; L243), PE-Dazzle 594 anti human CD1c (Clone; L161), BV605 anti human CD14 (Clone; M5E2), BV655 anti human CD86 (Clone; IT2.2), BV650, mouse IgG2b k isotype control, A700 anti human CD16 (Clone;3G8). Antibodies from BD Biosciences included; PE anti human CD169, PE-Cy7 anti human CD123 (Clone; 7G3), BV421 anti human Clec9A (Clone; 3A4), BV711 anti human CD80 (Clone;307.4), BV785 anti human CD11c (Clone; S-HCL-3), BUV395 anti human CD3 (Clone; SP-34-2), BUV496 anti human CD8a (Clone; RPA-T8) and BUV737 anti human CD20 (Clone; 2H7). Cells were stained for surface markers, fixed and acquired as detailed with the ICS assay.

MSD cytokine assay.

Briefly, 25μL microliters of plasma from each samples were combined with the biotinylated antibody plus the assigned linker and the SULFO-TAG™ conjugated detection antibody; in parallel a multi-analyte calibrator standard was prepared by doing 4-fold serial dilutions. Read buffer was added to both samples and calibrators and loaded in a 10-spot U-PLEX plate, which was read by the MESO QuickPlex SQ 120. The plasma cytokines values (pg/mL) were extrapolated from the standard curve of each specific analyte. Cytokine clustering was performed using independent methods: gap statistic method to identify and characterize optimal number of k-means clusters, and hierarchical clustering ward clustering; Euclidean distance.

Viral stock

Vero E6 cell line (African Green Monkey Kidney cell line; CRL-1586, ATCC) was cultured and maintained in MEM (Sigma) supplemented with 10% heat inactivated fetal bovine serum (FBS) (Gibco) and 1 mM L-glutamine (Gibco), 50U/ml penicillin and 50μg/ml streptomycin (Gibco). The cells were kept at 37°C in the presence 5% CO_2. At the time of virus inoculation and propagation, the concentration of FBS was reduced to 2%. SARS-CoV-2 (NR-52281: BEI Resources, Manassas, VA; USA-WA/2020, Lot no. 70033175) was passaged on Vero E6 cells at a MOI of 0.01 to produce the infectious viral stock. SARS-CoV-2 has been propagated and titrated by $TCID_{50}$ method followed by storage of aliquots at -80°C until further use in the experiments. Back titration of viral stocks via plaque assay was used to determine the infectious dose delivered to the RMs. The virus stock was also directly sequenced via metagenomic methods prior to inoculation to confirm the presence of the furin cleavage motif, which has been shown to be lost upon sequential passage of SARS-CoV-2 in culture(*73*). Our stock contained fewer than 6% of viral genomes with a mutation that could potentially abrogate furin-mediated cleavage of S.

Quantifying viral load RNA:

Quantitative PCR (qPCR) was performed on total viral RNA using the N2 primer and probe set designed by the CDC for their diagnostic algorithm: CoV2-N2-F: 5'-TTACAAACATTGGCCGCAAA-3', CoV2-N2-R: 5'-GCGCGACATTCCGAAGAA-3', and CoV2-N2-Pr: 5'-FAM-ACAATTTGCCCCCAGCGCTTCAG-BHQ-3'. The primer and probe sequences for the sub-genomic mRNA or sgRNA transcript of the E gene are SGMRNA-E-F: 5'-CGATCTCTTGTAGATCTGTTCTC-3', SGMRNA-E-R: 5'-ATATTGCAGCAGTACGCACACA-3', and SGMRNA-E-Pr: 5'-FAM-ACACTAGCCATCCTTACTGCGCTTCG-3'. qPCR reactions were performed in duplicate with the Thermo-Fisher 1-Step Fast virus mastermix using the manufacturer's cycling conditions, 200nM of each primer, and 125nM of the probe. The limit of detection in this assay was 90 copies per mL of VTM/BAL. To verify sample quality the CDC RNase P p30 subunit qPCR was modified to account for rhesus macaque specific polymorphisms. The primer and probe sequences are RM-RPP30-F 5'-AGACTTGGACGTGCGAGCG-3', RM-RPP30-R 5'- GAGCCGCTGTCTCCACAAGT-3', and RPP30-Pr 5'-FAM-TTCTGACCTGAAGGCTCTGCGCG-BHQ1-3'. A single well from each extraction was run as above to verify RNA integrity and sample quality via detectable and consistent cycle threshold values (Ct between 25-32).

Histopathology:

For each animal, all the lung lobes were used for analysis and affected microscopic fields were scored semi-quantitatively as Grade 0 (None); Grade 1 (Mild); Grade 2 (Moderate) and Grade 3 (Severe). Scoring was performed based on these criteria: number of lung lobes affected, type 2 pneumocyte hyperplasia, alveolar septal thickening, fibrosis, perivascular cuffing, peribronchiolar hyperplasia, and inflammatory infiltrates. A total pathological score was calculated by the accumulative scores from each criterion of all lobes affected, and an average pathological score was calculated by combining scores from each criterion taking into account

the number of lobes affected. Digital images of H&E stained slides were captured at 20x and 10x magnification with an Olympus BX43 microscope equipped with a digital camera (DP27, Olympus) using Cellsens® Standard 2.3 digital imaging software (Olympus).

Statistics:

The repeated measures linear mixed model included three predictors (study group, time on study (categorical) and the statistical interaction between study group and time on study). A compound-symmetric variance-covariance form in repeated measurements was used for total viral RNA (and sqRNA) and robust estimates of the standard errors of parameters were used to perform statistical tests and construct 95% confidence intervals. A P-value ≤0.05 was considered statistically significant for the main effects (study group and time on study) and for the study group by time on study interaction effect from the repeated-measures analysis. The statistical test for interaction between time on study and study group was the overall hypothesis test to determine whether total viral RNA (and sgRNA) in the three study groups changed in significantly different ways during follow-up (i.e., different temporal patterns by study group). If the statistical interaction was not significant, then the main effect test for study group was used as the primary hypothesis test to compare the 3 study groups. If a significant interaction was detected, then t-tests were used to compare the differences between the model-based study group means at each time point and to compare differences over time within each study group. Specific statistical tests will be done within the framework of the mixed linear model. All statistical tests were 2-sided and unadjusted for multiple comparisons. After analysis, total viral RNA and sgRNA were back transformed to the original scale and reported as the geometric mean. The same analytic plan was used for the antibody titer outcomes. All repeated-measures analyses were implemented via the SAS MIXED Procedure (version 9.4; SAS Institute, Cary, NC), providing separate estimates of the mean for each outcome by time on study and treatment group.

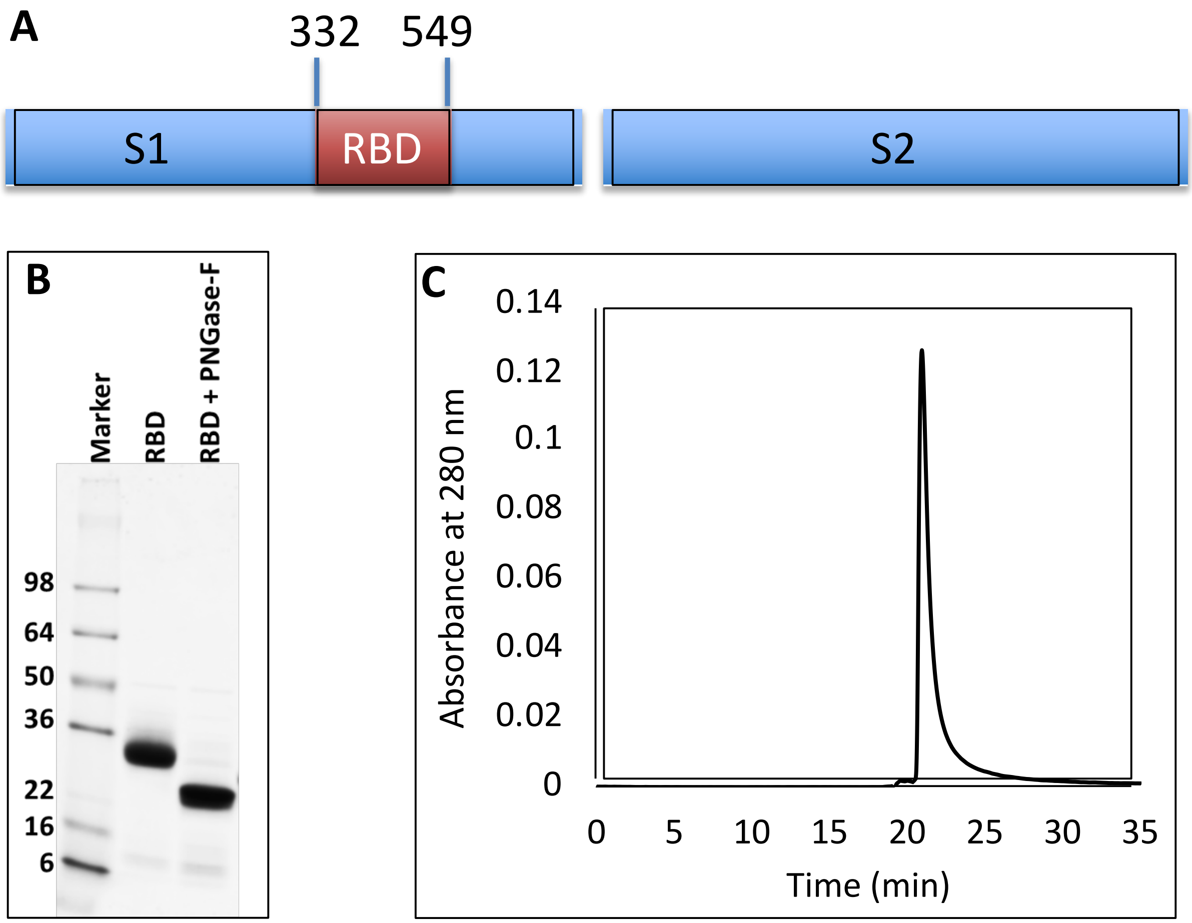

Fig. S1. SDS-PAGE and size exclusion HPLC of yeast expressed SARS-CoV-2 RBD. (A) Diagram of SARS-CoV-2 Spike protein and RBD region. (B) 6μg of SARS-CoV-2 RBD before and after PNGase-F treatment were loaded on a 4-20% Tris-glycine gel in a reduced condition. The size reduced after PNGase-F treatment suggested that RBD was N-glycosylated. (C) The purity of SARS-CoV-2 RBD was evaluated using SE-HPLC. Briefly, 50μg of RBD was injected into a Yarra SEC-3000 column (300mm X 7.8mm; catalog # 00H-4513-K0), and was eluted in 20mM Tris, 150mM NaCl, pH 7.5 at the flow rate of 0.6 ml/min. The % purity was estimated as ~99%.

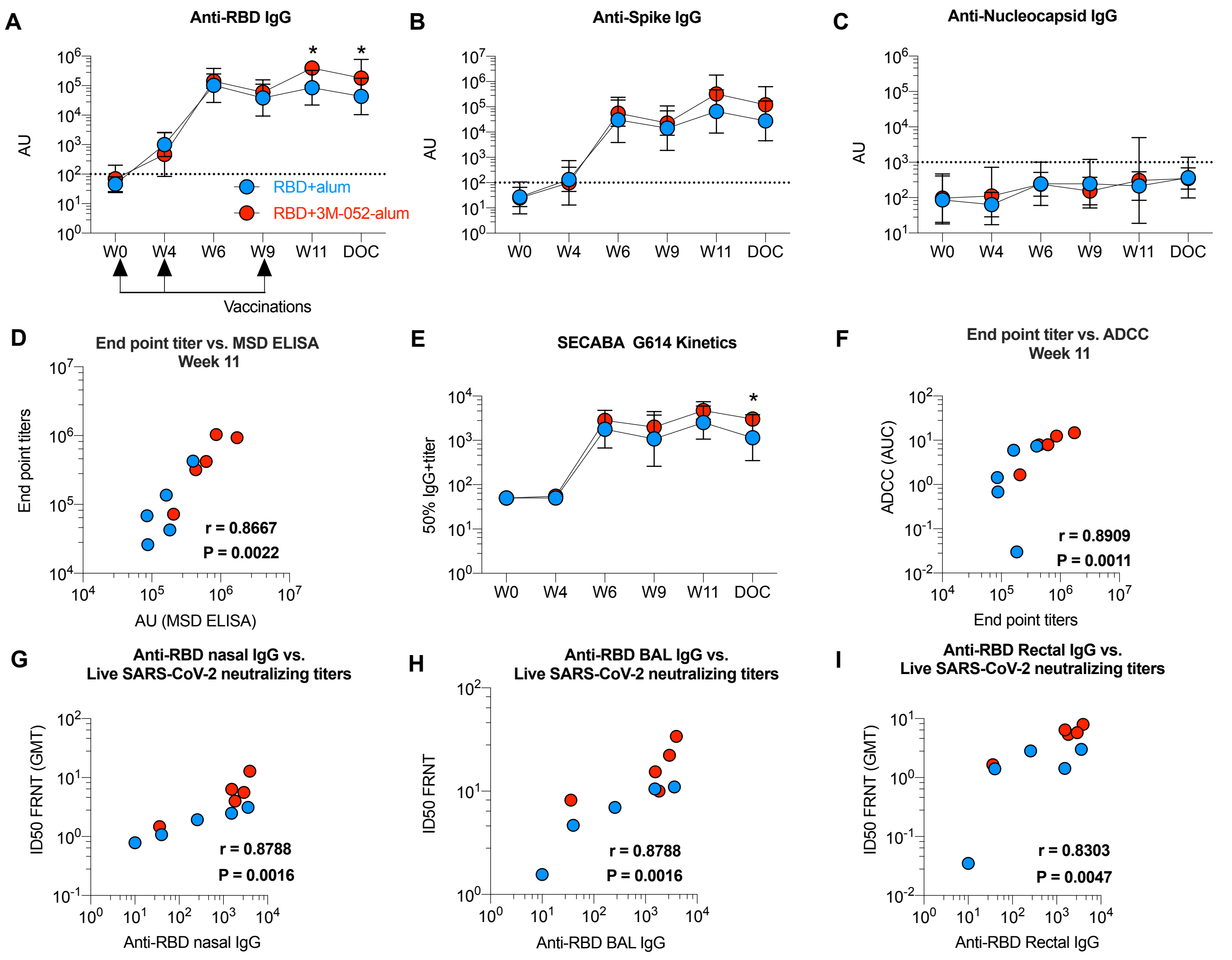

Fig. S2. Multiplexed- analyses of Ab responses in serum, SECABA activity and correlation of serum and mucosal Ab responses. A 4-plex kit from Meso Scale Discovery was used to simultaneously measure binding Ab responses to RBD, whole S and nucleocapsid SARS-CoV-2 proteins. A), B), and C) Line graphs indicate the magnitude of anti-RBD, anti-whole S, and anti-nucleocapsid binding Ab responses respectively. Geometric mean titers (GMT) with 95% CI are shown (n = 5 per group). D) Correlation of the multiplexed ELISA-based binding Ab response with end point titers at week 11 (peak) is shown. E) Line graphs indicate SECABA activity against the G614 strain. Geometric mean titers (GMT) with 95% CI are shown (n=5 per group). F) Correlation of NK cell degranulation activity with binding Ab response with end point titers at week 11 (peak) is shown. G) Correlation of Live SARS-CoV-2 neutralizing activity with binding Ab response in nasal swabs at week 11 (peak) is shown. H) Correlation of

Live SARS-CoV-2 neutralizing activity with binding Ab response in BAL at week 11 (peak) is shown. I) Correlation of Live SARS-CoV-2 neutralizing activity with binding Ab response in rectal swabs at week 11 (peak) is shown. Spearman's correlation analysis was used to compare end point titers and MSD anti-RBD binding responses. Spearman's r and p values are indicated on the graphs. Repeated measures analyses were used to test for significant statistical differences in line graphs A,B,C and E as detailed in methods. * in panel A = 0.02 at W11 and 0.04 on the DOC. * in panel E is P = 0.02.

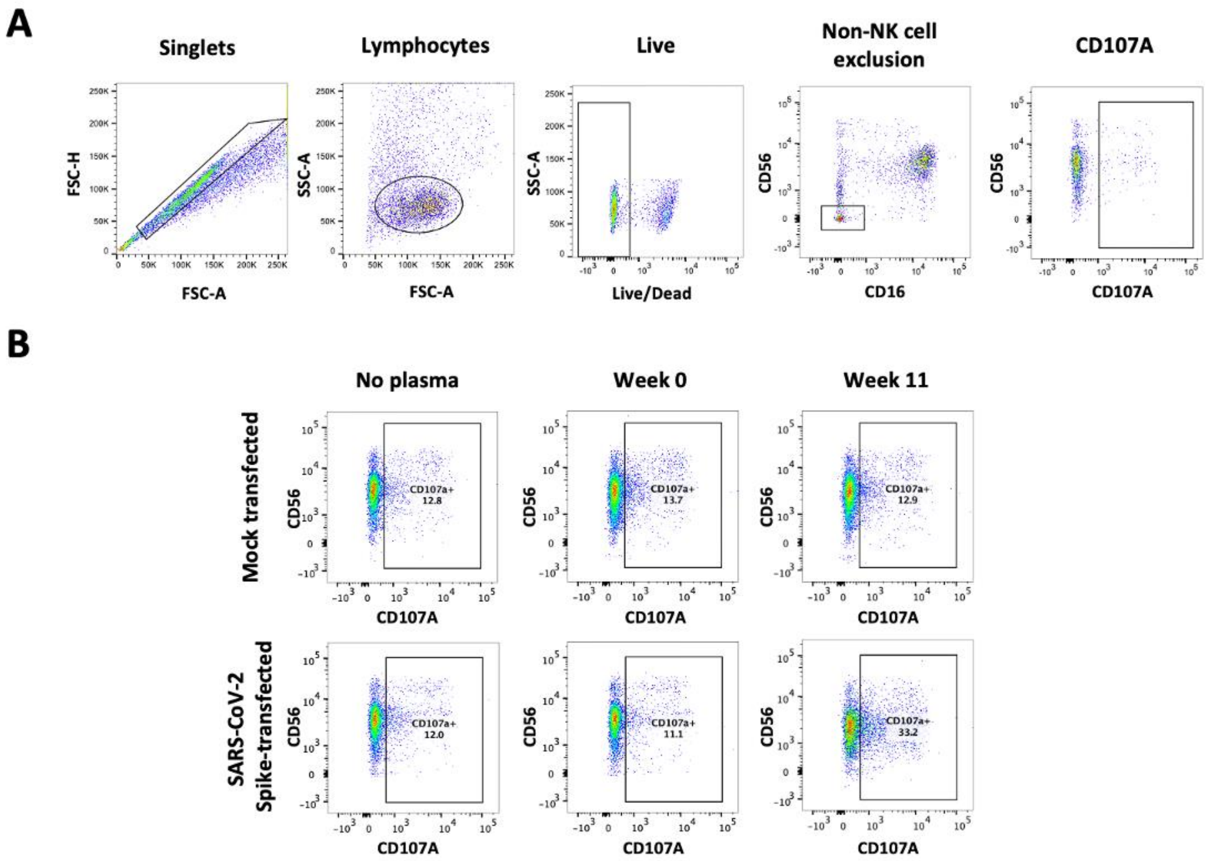

Fig. S3. Gating strategy and representative plots for NK cell degranulation assay.

(A) To analyze cells acquired by flow cytometry, singlets, lymphocytes, live cells and live cells were selected. Non-NK cells were then excluded and of NK cells, the percentage of CD107A+ cells was determined. (B) Representative plots of CD107A+ NK cells in the presence of mock transfected and SARS-CoV-2 Spike transfected cells and either no, baseline (week 0) or peak immunogenicity (week 11) plasma.

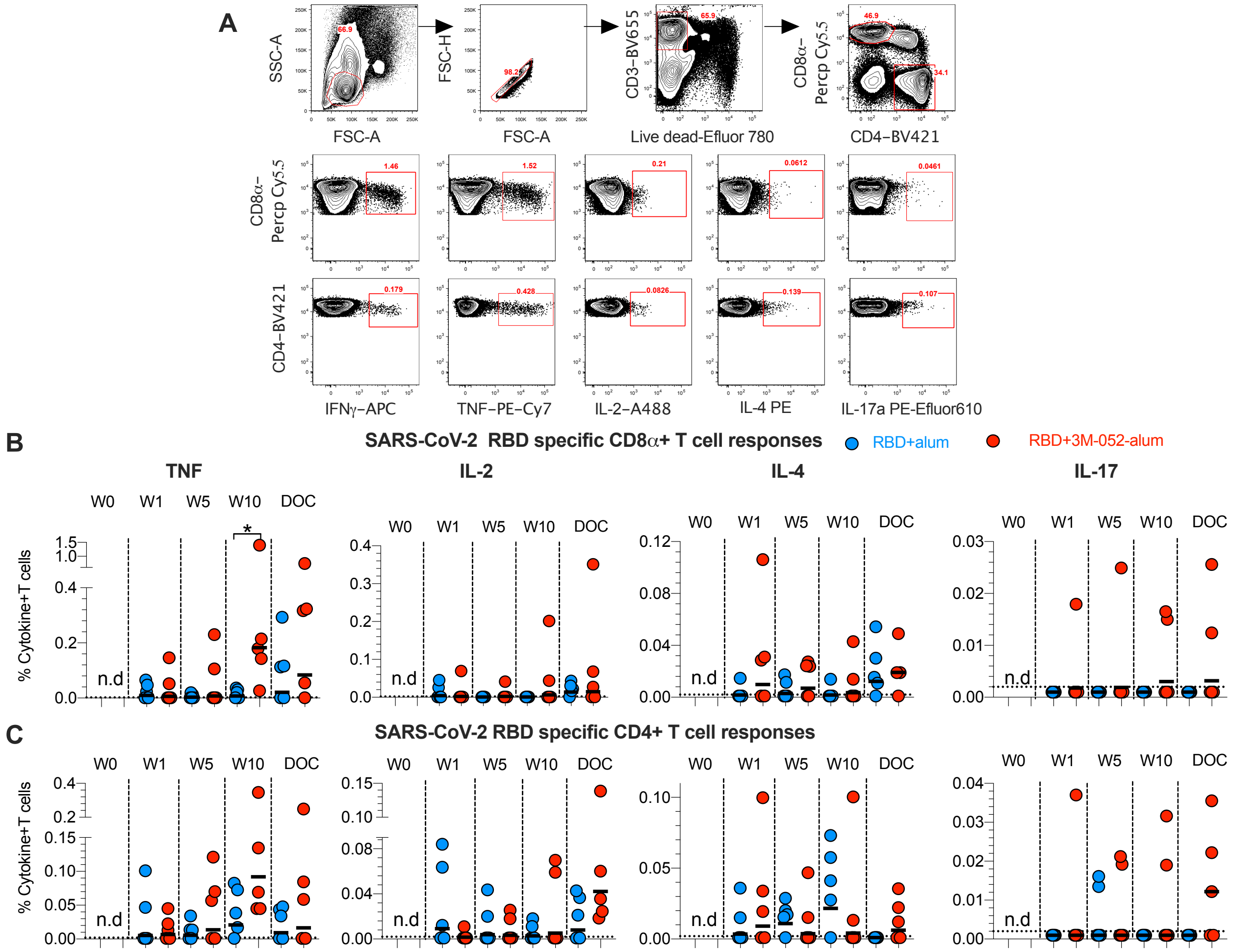

Fig. S4. Flow cytometry gating strategy in identifying CD8+ and CD4+ T cells and cytokine production upon ex-vivo RBD peptide pool stimulation. A) Flow cytometry gating strategy in identifying intracellular cytokine positive CD8+ and CD4+ T cells is shown. Scatter plots summarize cytokine positive B) CD8+ T cells and C) CD4+ T cells post ex-vivo RBD peptide pool stimulation at various time points post vaccination. The horizontal bar represents the Geometric mean. A two tailed Mann-Whitney test was used to compare the significance of the difference between groups 2 and 3. * represents a P value =0.0317.

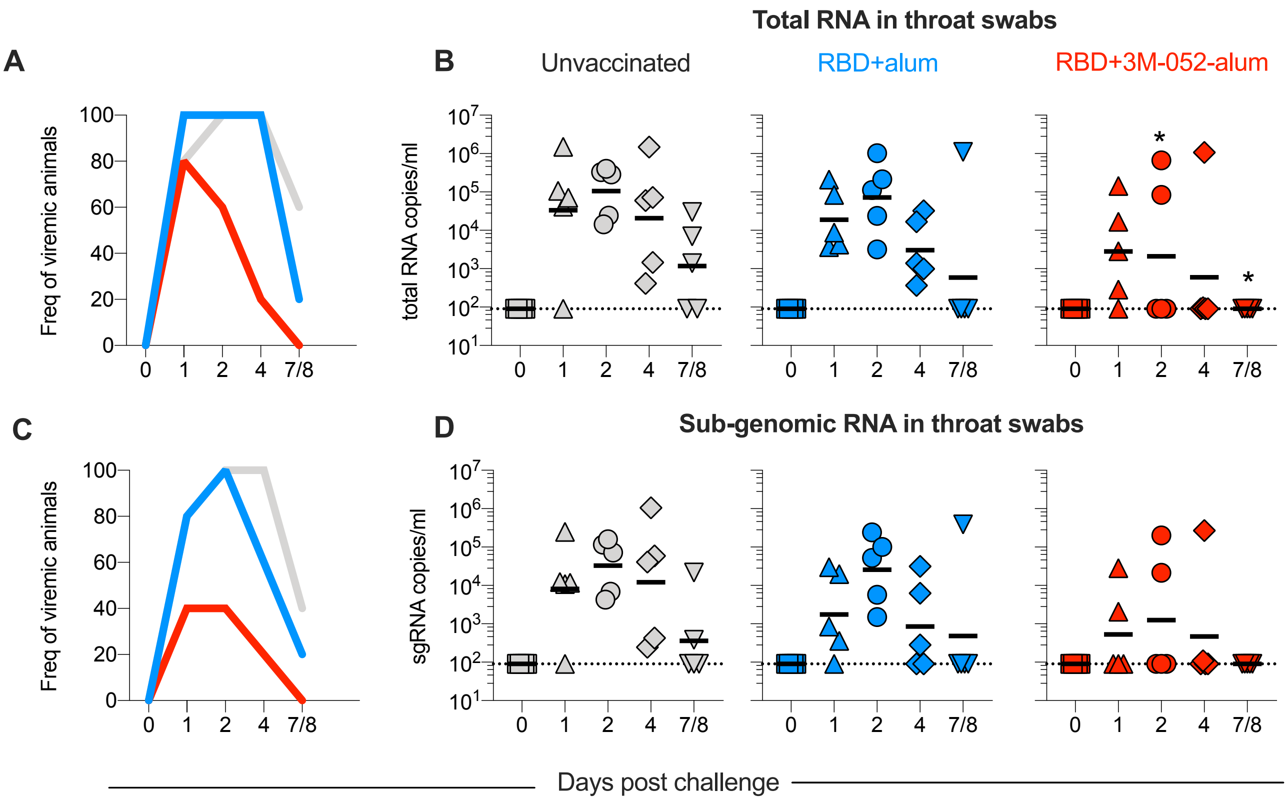

Fig. S5. Vaccination with RBD+3M-052-alum reduces total and sgRNA in throat swabs of RMs post respiratory challenge. A) Line graph indicates frequencies of animals testing positive for total RNA in throat swabs post challenge. B) Scatter plots indicate total SARS-CoV-2 RNA levels measured in throat swabs post challenge in all treatment groups. C) Line graph indicates frequencies of animals testing positive for sub-genomic (sgRNA) in throat swabs post challenge. D) Scatter plots indicate sgRNA levels measured in throat swabs post challenge. Horizontal lines in graphs B and D represent Geometric means. Repeated measures analyses were performed on data with viral loads to compare differences over time between the study groups as detailed in methods. * in panel B = 0.043 at day 2 and 0.018 at day 7/8.

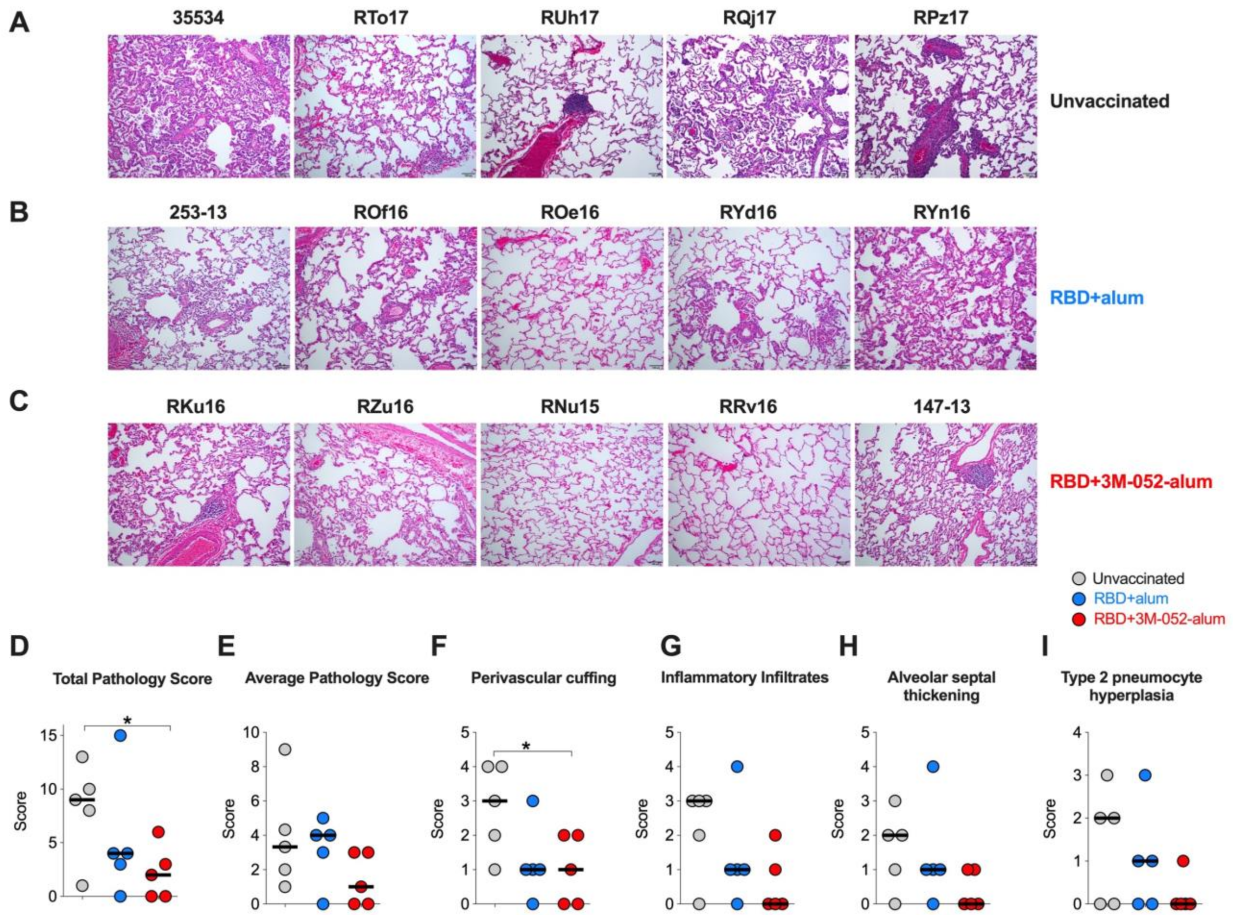

Fig. S6. Photomicrographs documenting lung pathology in RMs upon respiratory challenge with SARS-CoV-2. Photomicrographs and graphs in the figure summarize severity of lung inflammation scored by two independent pathologists in a blinded manner and pathology scores are represented. Scale bar =100µm. A) Photomicrographs show hematoxylin and eosin stained sections of lung lobes at (10x) magnifications from A) Unvaccinated, B) RBD+alum vaccinated, and C) RBD+3M-052-alum vaccinated animals. Scale bars = 100µM. D,E) Graphs summarize total and average pathology scores. F, G, H, and I) summarize perivascular cuffing, inflammatory cell infiltrates, alveolar septal thickening and type 2 pneumocyte hyperplasia. One-tailed Mann-Whitney test was specifically used to assess impact of vaccination on viral load reduction and the significance of difference between groups 2, 3 with unvaccinated animals in group 1. * represents a P value = 0.0278 in panel D and P = 0.0397 in panel F.

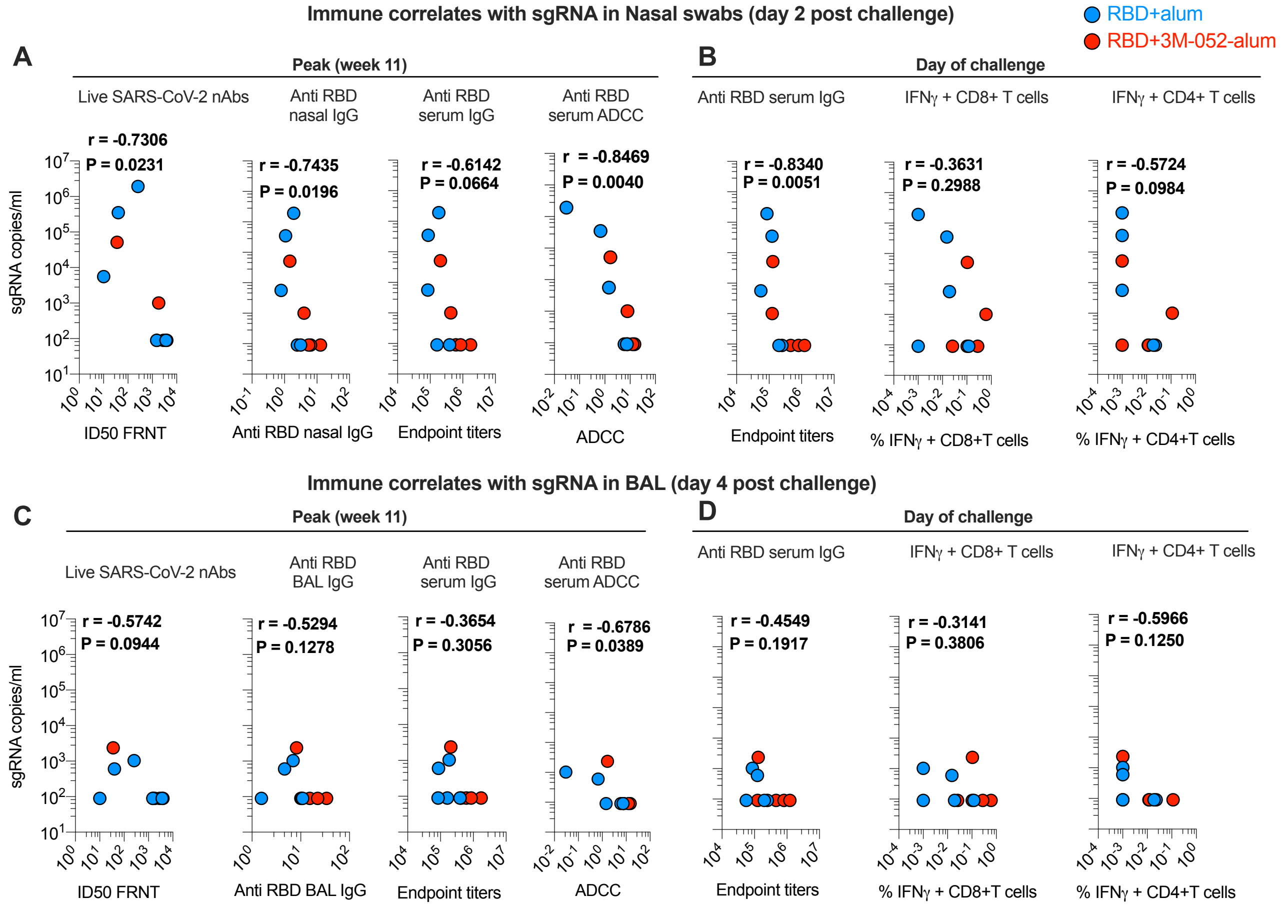

Fig. S7. Anti-RBD Ab responses correlate with viral sgRNA in URT. Correlations of anti-RBD Ab and T cell responses with sub-genomic viral load in URT and LRT were evaluated at both peak and the day of challenge (DOC). Graphs in A) and B) highlight correlations between Ab and T cell responses induced by vaccines in the study with sub-genomic SARS-CoV-2 viral RNA in nasal swabs. C) and D) highlight correlations between Ab and T cell responses induced by vaccination with sub-genomic SARS-CoV-2 viral RNA in BAL. Spearman's correlation was used to identify significance with P and r-values of the test are shown.

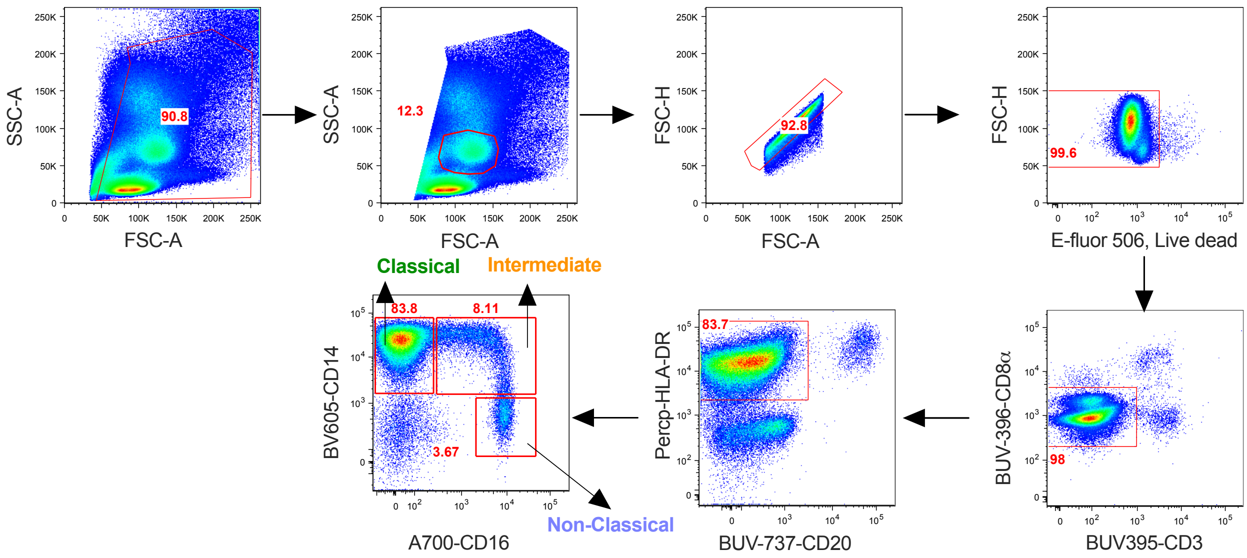

Fig. S8. Flow cytometry gating scheme to identify blood monocytes in RMs.

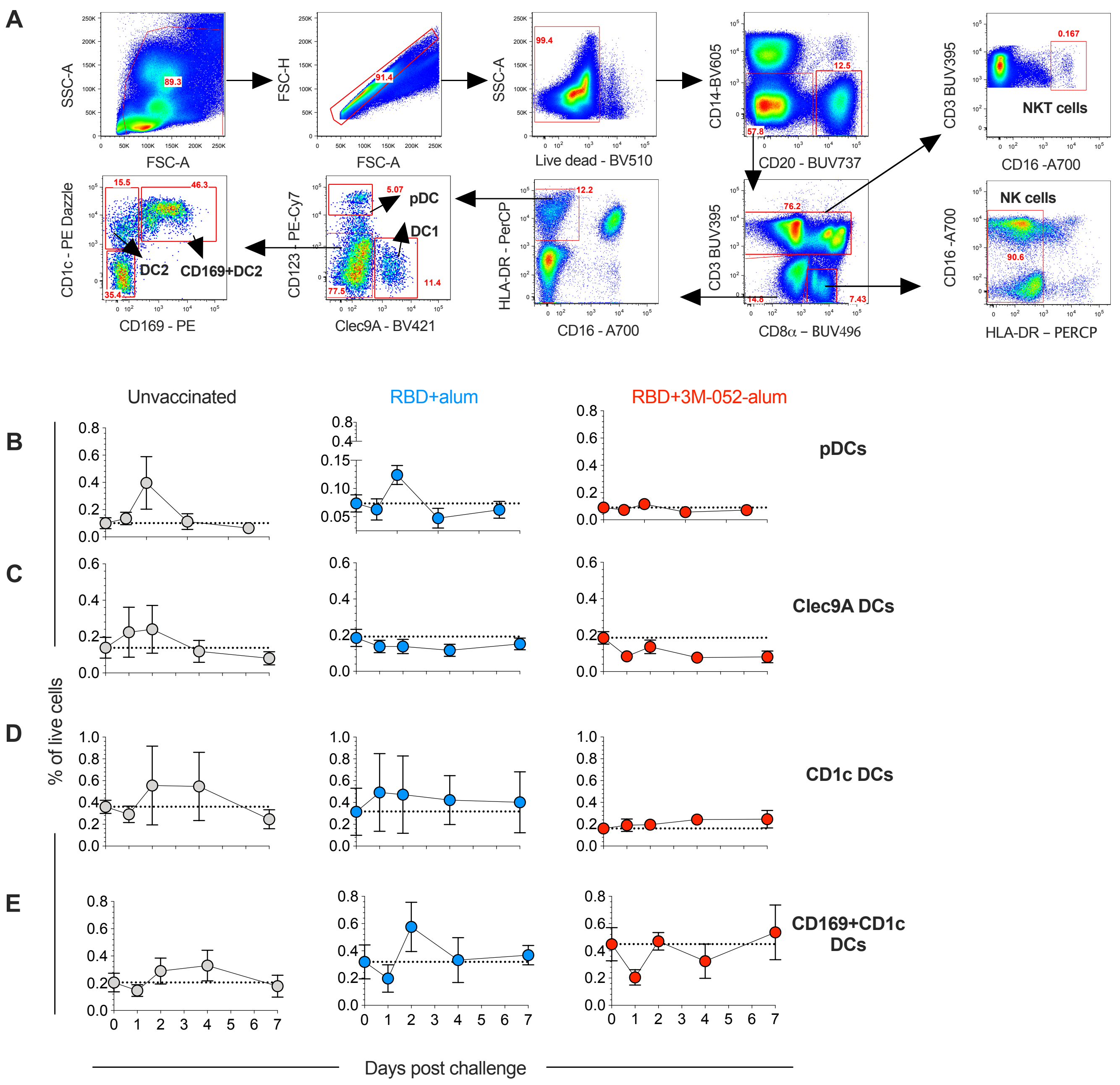

Fig. S9. Flow cytometry gating strategy in identifying DC and lymphocyte subsets in RM PBMCs and changes in DC subsets post SARS-CoV-2 challenge. A) Representative flow plots from one RM are shown to highlight the gating strategy used in identifying DC and lymphocyte cell subsets in RM PBMCs. Line graphs show changes in frequencies of B) pDCs, C) Clec9A+ DCs, D) CD1c+CD169-ve DCs, and E) CD1c+CD169+ DCs post SARS-CoV-2 challenge in all animals. Mean and standard error (SEM) is reported. Significance of change in

frequencies in comparison with baseline was tested using the Wilcoxon matched-pair signed-rank test.

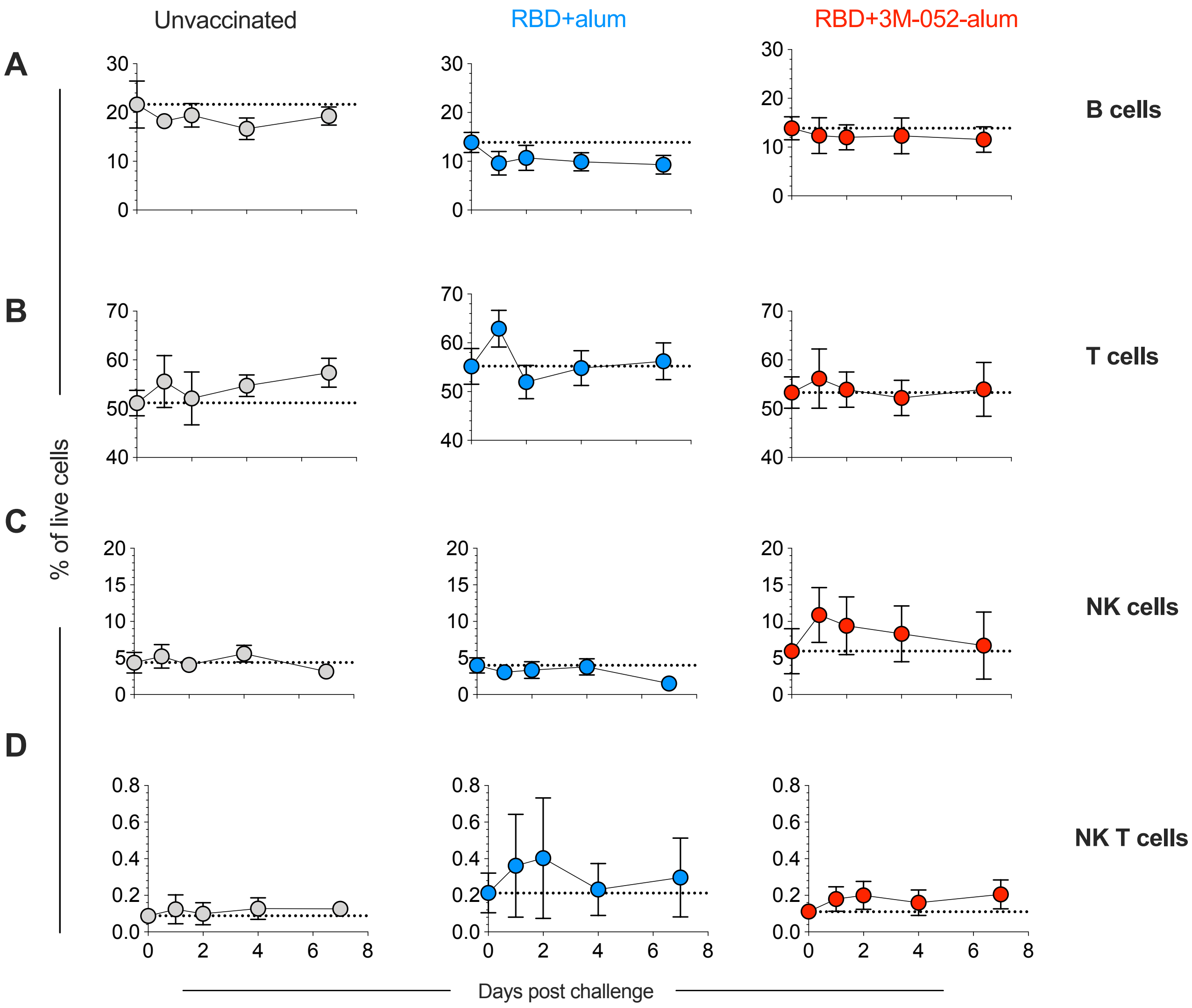

Fig. S10 Changes in frequencies of lymphocyte subsets in RM PBMCs post SARS-CoV-2 challenge. Line graphs show changes in frequencies of A) B cells, B) T cells, C) NK cells and D) NK T cells post SARS-CoV-2 challenge in all animals. Flow gating strategy to identify these cells is detailed in Fig. S7. Mean and standard error (SEM) is reported. Significance of change in frequencies in comparison with baseline was tested using the Wilcoxon matched-pair signed-rank test. A cut-off of $p<0.05$ was used for significance.

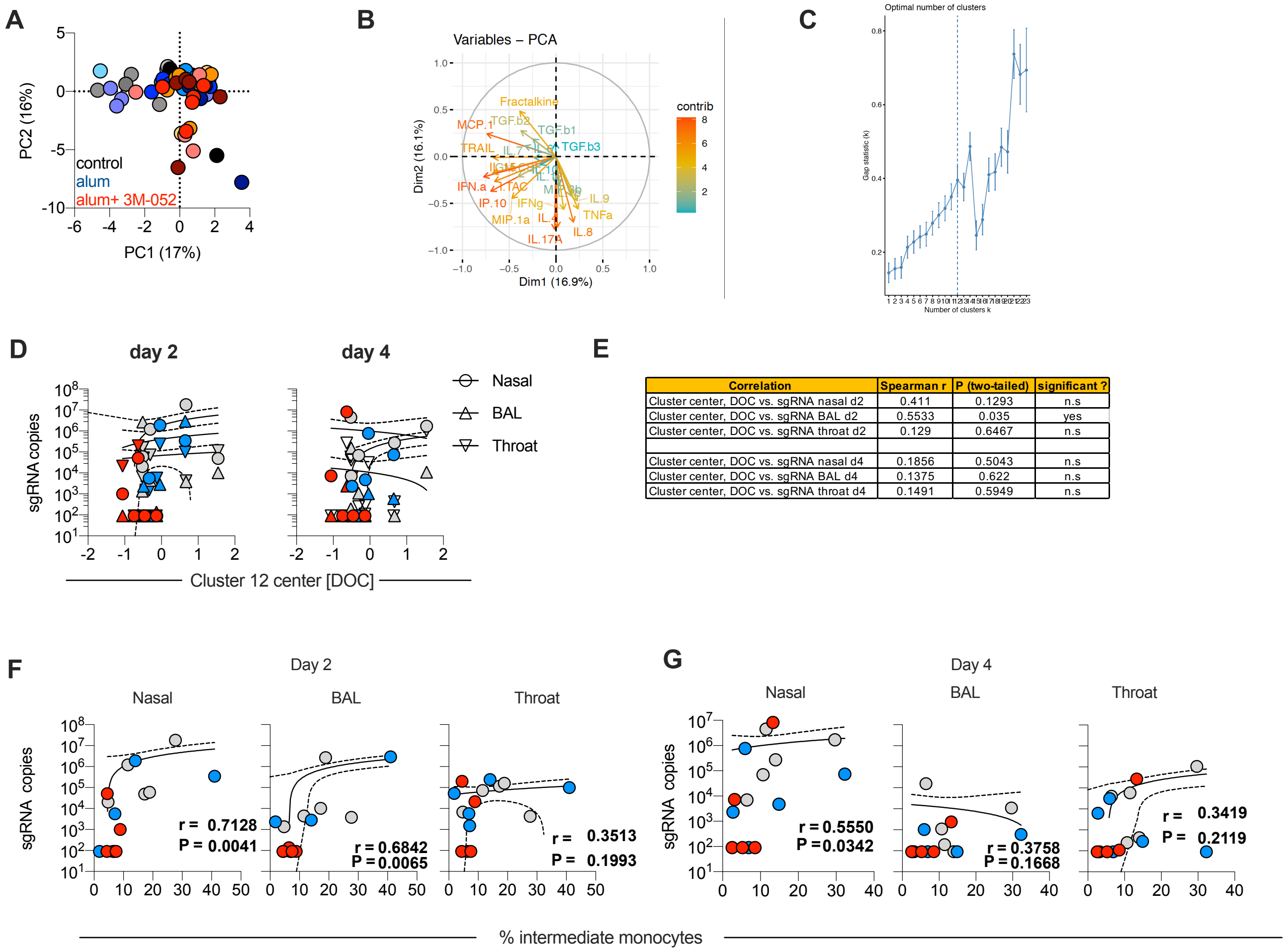

Correlation	Spearman r	P (two-tailed)	significant ?
Cluster center, DOC vs. sgRNA nasal d2	0.411	0.1293	n.s
Cluster center, DOC vs. sgRNA BAL d2	0.5533	0.035	yes
Cluster center, DOC vs. sgRNA throat d2	0.129	0.6467	n.s
Cluster center, DOC vs. sgRNA nasal d4	0.1856	0.5043	n.s
Cluster center, DOC vs. sgRNA BAL d4	0.1375	0.622	n.s
Cluster center, DOC vs. sgRNA throat d4	0.1491	0.5949	n.s

Fig. S11. Principal component analyses (PCA) of multiplexed soluble factors in plasma and correlation of Cluster 12 center and frequencies of blood IMs with sgRNA. A) Principal component analysis of plasma soluble factors post challenge. The x and y axes represent PC1 percentage and PC2 percentage, respectively. Each dot presents an independent subject. Ctr (control) is presented in shades of gray dots, alum presented in shades of blue and the alum+3M-052 is presented in shades of red dots – darker colors are the latest time points (from D0-7); B) Relative contribution of the evaluated variables in PC1. The x and y axes represent the percentage of Dim1 (dimension 1) and the percentage of Dim2 (dimension 2), respectively. Contribution of each variable from high to low is presented as gradient color from light red to cyan color. C) Determining the optimal number of clusters. The x and y axes represent the

number of clusters k and gap statistics (k), respectively. The cut off for the optimal number of clusters is indicated by a vertical blue dotted line**.** D) Graphs highlight correlation of the Cluster 12 center on the day of challenge (DOC) with sgRNA viral loads at days 2 and 4 (peak) in nasal and throat swabs as well as BAL. E) summarizes spearman's correlation values, F) Graphs highlight correlation of frequencies of intermediate monocytes with sgRNA at days 2 and 4 in nasal and throat swabs as well as BAL. Spearman's correlation was used to identify significance. P and r values of the Spearman's correlation test are indicated.

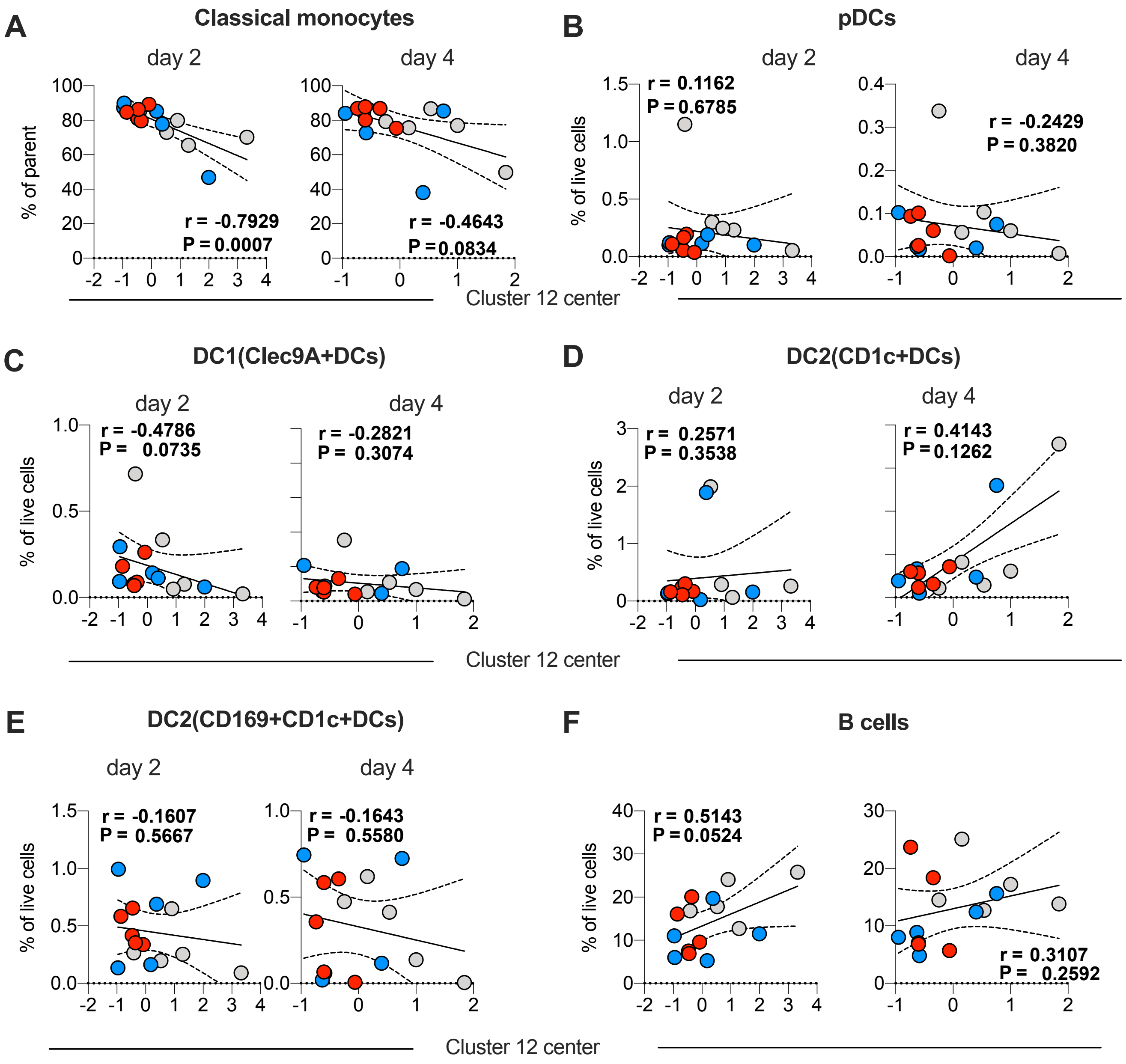

Fig. S12. Correlation of cluster 12 center with frequencies of DC subsets and B cells post challenge. Graphs highlight the correlation of the Cluster 12 center with the frequencies of DC subsets (A-E) and B cells (F) at days 2 and 4 in PBMCs. Spearman's correlation was used to identify significance. P and r values of the Spearman's correlation test are indicated.

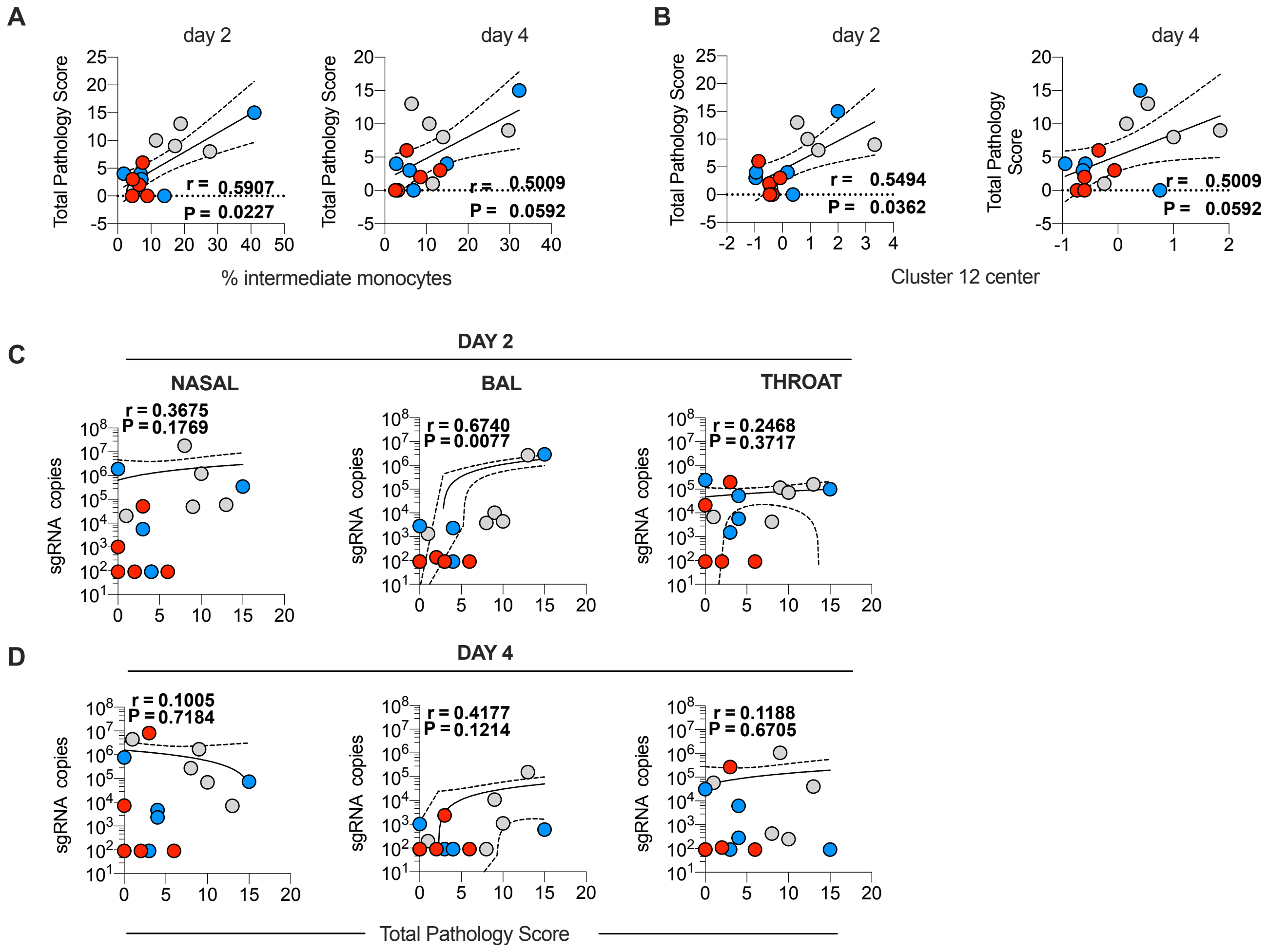

Fig. S13. Correlation of frequencies of intermediate monocytes, cluster 12 center and viral loads with lung pathology. Graphs show the correlation of % of intermediate monocytes among total and Cluster 12 center values at days 2 and 4 with lung pathology score at necropsy (A, B). Graphs in C) and D) show the correlation of sgRNA at day 2 and 4 in nasal, BAL and throat samples with lung pathology score at necropsy. Spearman's correlation was used to identify significance. P and r values of the Spearman's correlation test are indicated.

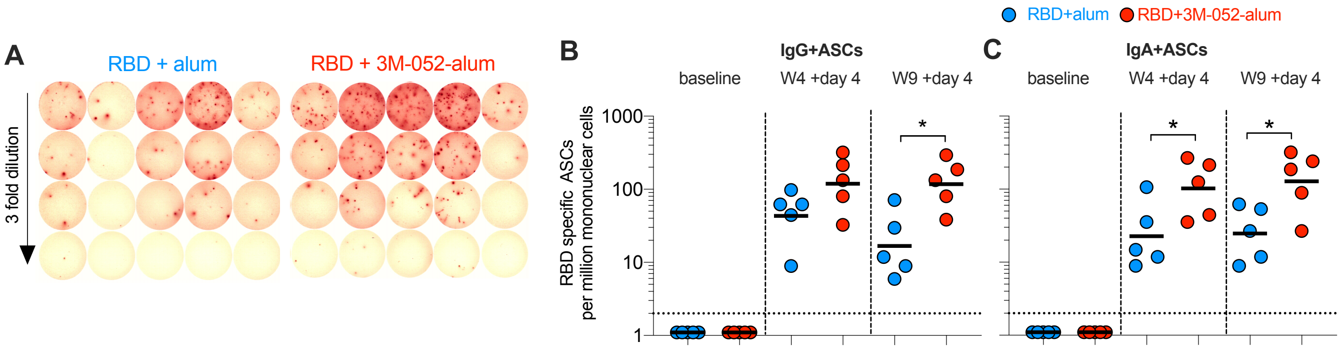

Fig. S14. Vaccination with RBD+3M-052-alum in comparison with alum induces higher RBD-specific blood ASCs. A) Scanned ELISPOT plate images are shown for IgG secreting RBD- specific plasmablasts for all animals at week 6+day 4, a peak time for induction of ASCs. B) Graph summarizes frequencies of RBD specific IgG+ ASCs in blood. A two tailed Mann-Whitney test was used to compare the significance of the difference between groups 2 and 3. * represents a P value = 0.0159 for panel B, 0.0397 for both time points in panel C.

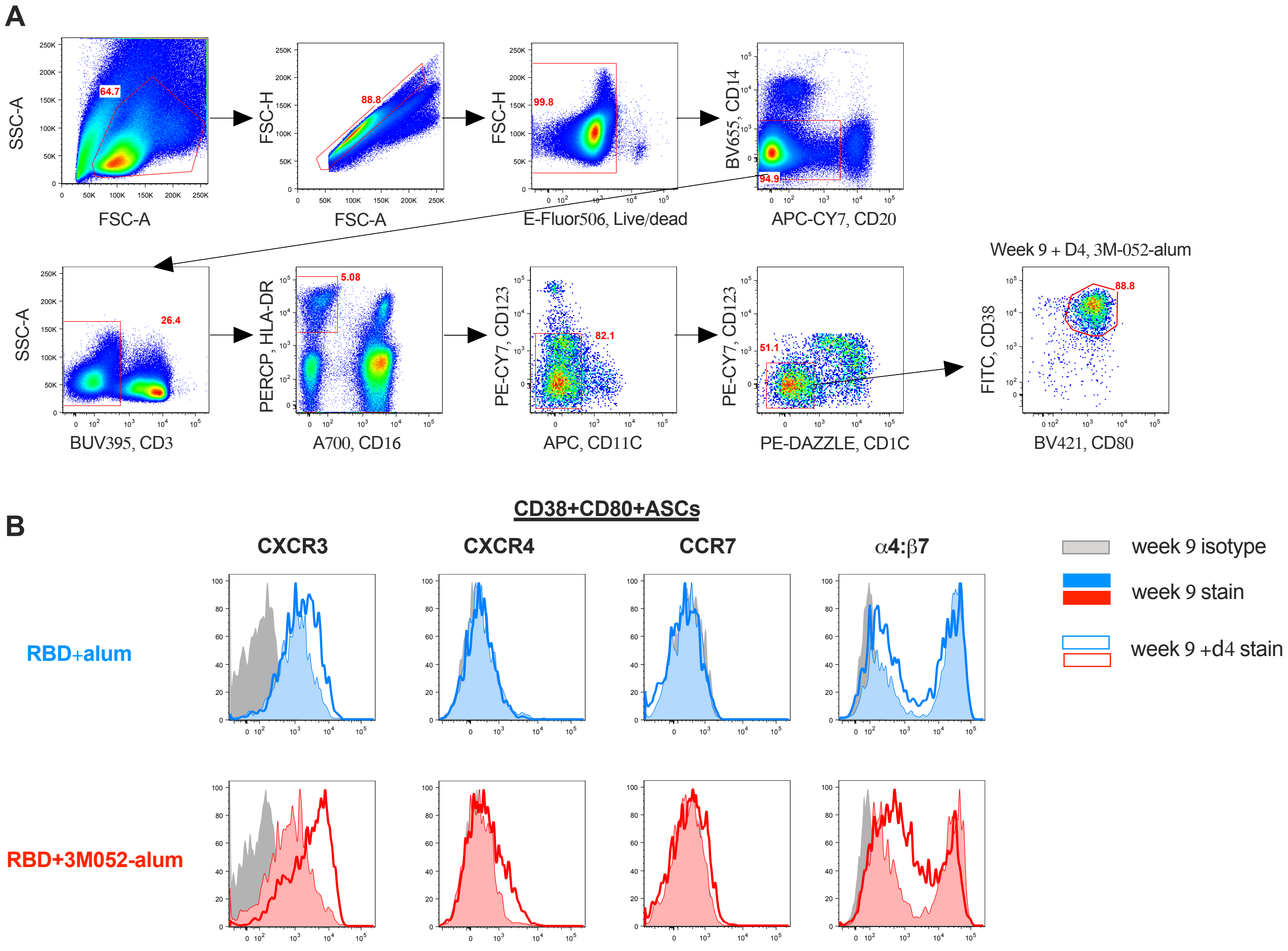

Fig. S15. Flow cytometry gating strategy used in identifying blood ASCs in RMs. A) Representative flow plots from one RM are shown to highlight the gating strategy used in identifying plasmablasts/blood ASCs in RM PBMCs. B) Representative flow plots from one RM each from the vaccinated groups highlight changes in expression of chemokine receptors CXCR3, CXCR4, CCR7 and alpha4:beta 7 post the third vaccination at week 9 in the study.

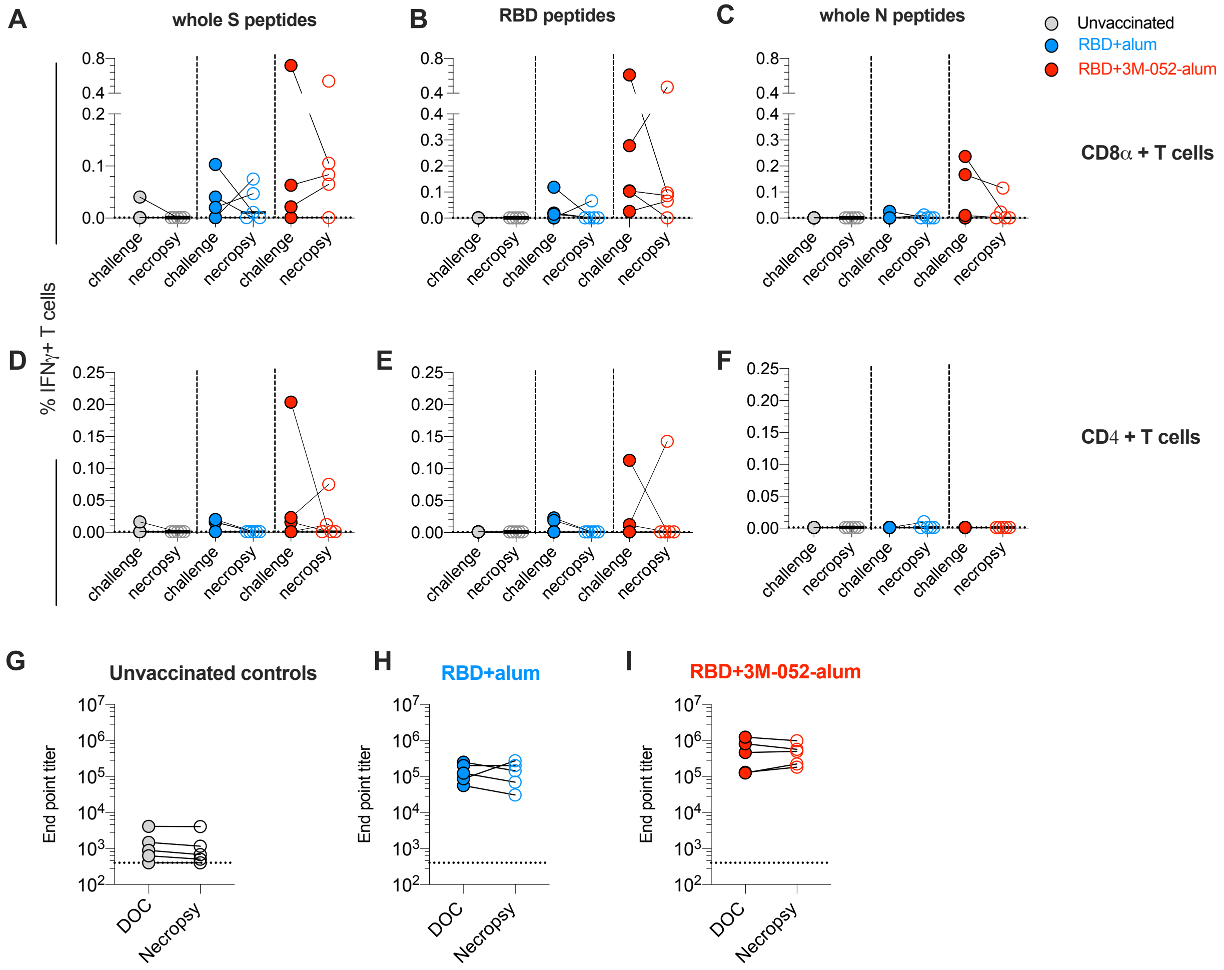

Fig. S16. Anamnestic T cell and Ab responses post challenge. Graphs represent anamnestic or recall T cell responses post SARS-CoV-2 challenge. Flow cytometry plots show IFN-γ secreting CD8+ T cell responses (A-C) and CD4+ T cell responses (D-F) to whole S (MP) and RBD peptide pool and N peptide pool stimulations ex-vivo. Binding ELISA titers were quantified on the day of challenge and at euthanasia in all unvaccinated and vaccinated animals (G-I).

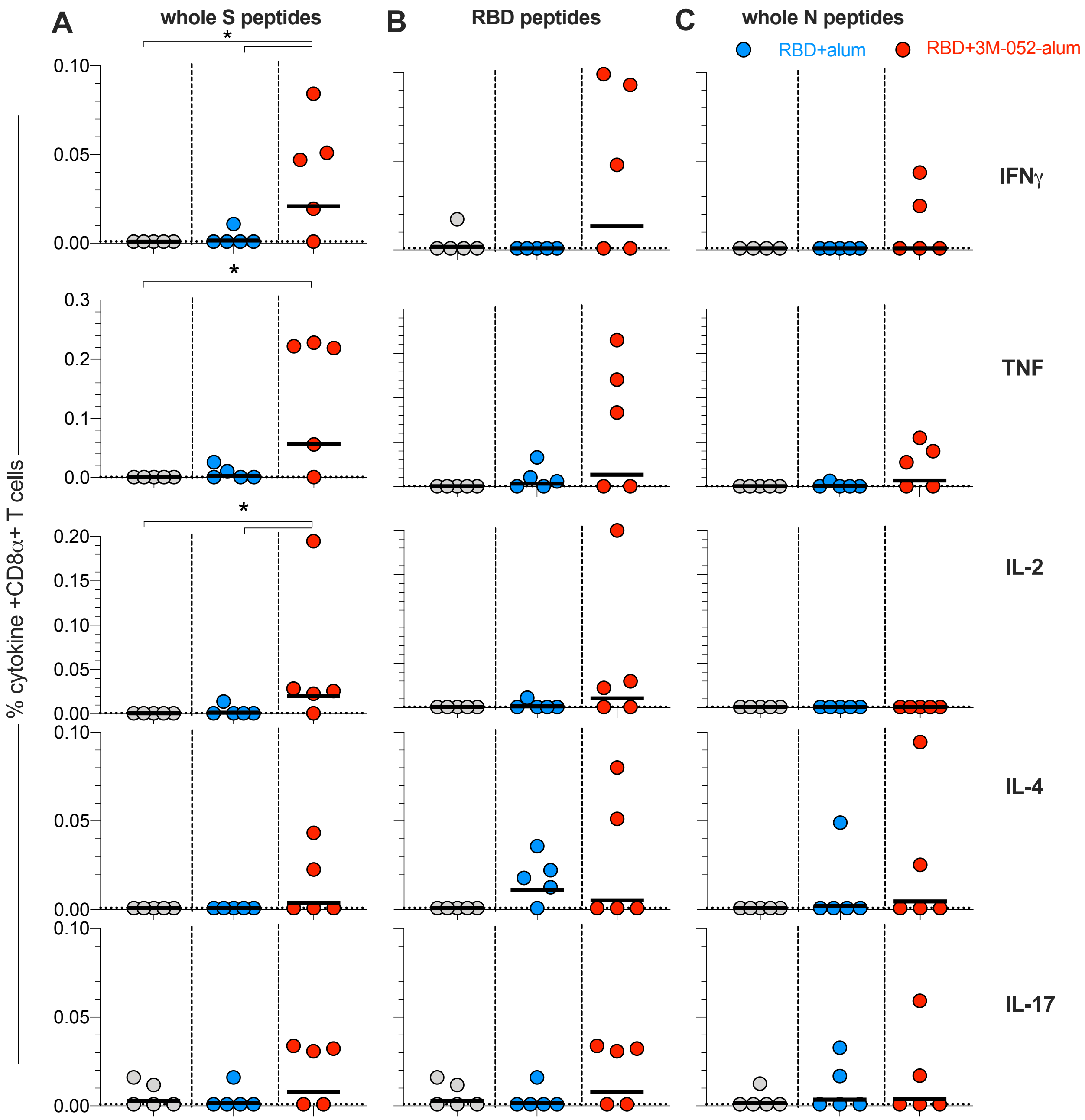

Fig. S17. Frequencies of SARS-CoV-2 specific CD8+T cells in hilar lymph nodes. CD8+T cell responses against RBD, whole S, and SARS-CoV-2 N proteins were assayed in lung draining hilar LNs at necropsy post challenge using an ex-vivo peptide pool stimulation assay. A two tailed Mann-Whitney test was used to compare the significance of the difference. * represents a P value = 0.0476.

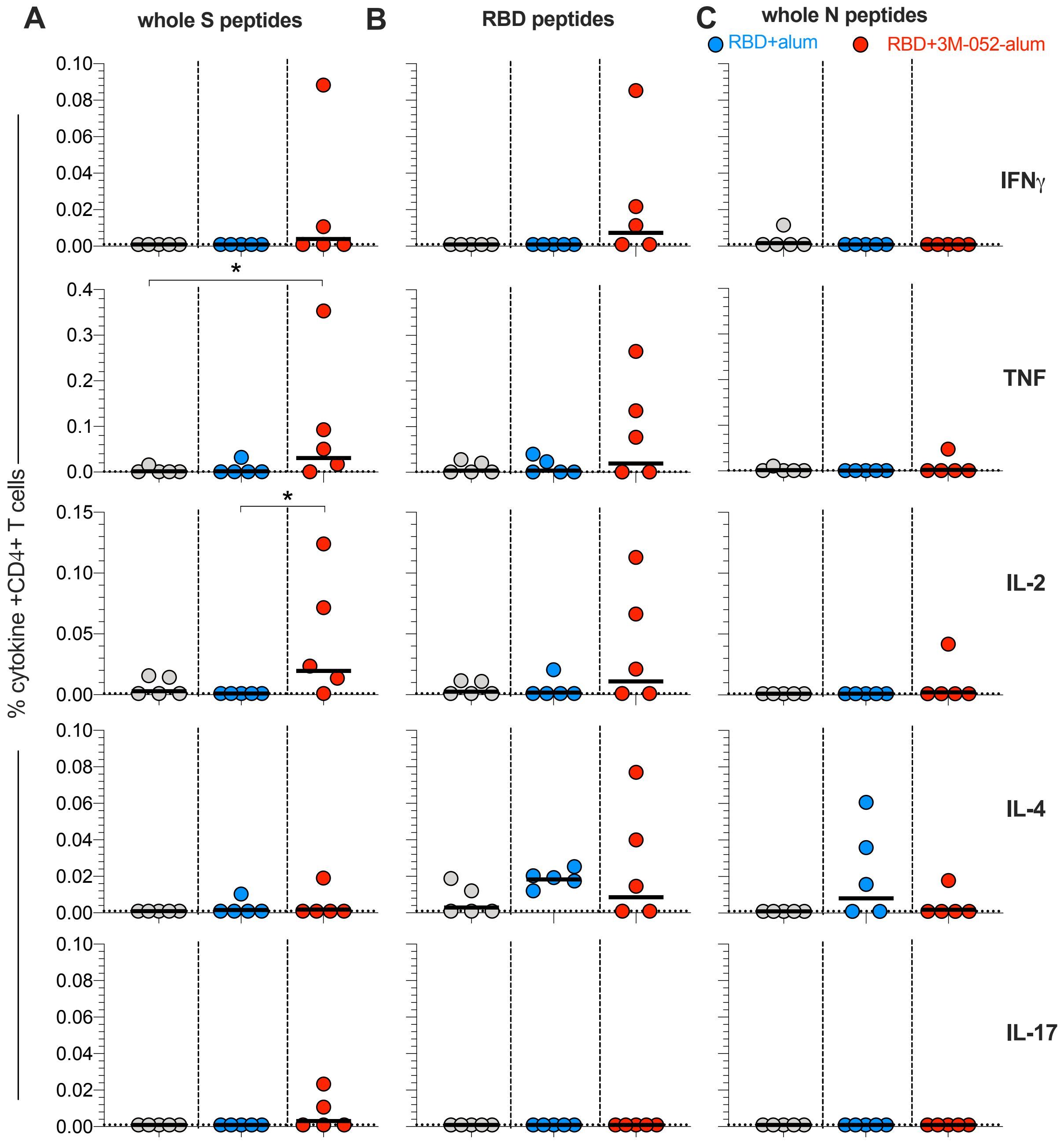

Fig. S18. Frequencies of SARS-CoV-2 specific CD4+T cells in hilar lymph nodes. CD4+T cell responses against RBD, whole S and SARS-CoV-2 N proteins were assayed in lung draining hilar LNs at necropsy post challenge using an ex-vivo peptide pool stimulation assay. A

two tailed Mann-Whitney test was used to compare the significance of the difference. * represents a P value = 0.0476.
