# Supplementary material for: A yeast-expressed RBD-based SARS-CoV-2 vaccine formulated with 3M-052-alum adjuvant promotes protective efficacy in non-human primates
Source: Sci Immunol. Author manuscript; Available in PMC 2022 May 19. (PMC9119307; doi:10.1126/sciimmunol.abh3634)
Supplement: Supplementary file 1 [file NIHMS1783029-supplement-Fig__S1.pdf]

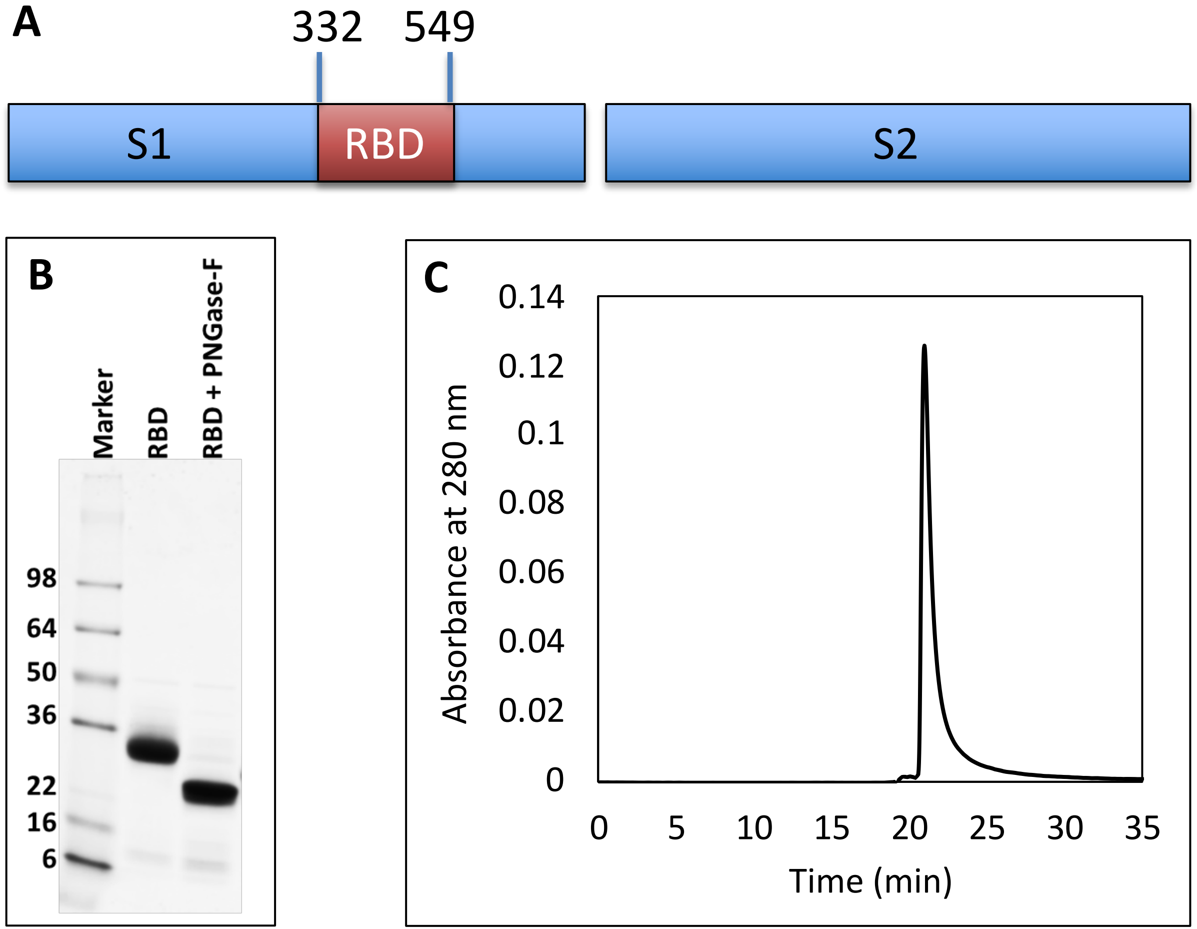
A
332
549
S1
RBD
S2
B
Marker
RBD
RBD + PNGase-F
98
64
50
36
22
16
6
C
Absorbance at 280 nm
0.14
0.12
0.1
0.08
0.06
0.04
0.02
0
0
5
10
15
20
25
30
35
Time (min)
