# Supplementary material for: A yeast-expressed RBD-based SARS-CoV-2 vaccine formulated with 3M-052-alum adjuvant promotes protective efficacy in non-human primates
Source: Sci Immunol. Author manuscript; Available in PMC 2022 May 19. (PMC9119307; doi:10.1126/sciimmunol.abh3634)
Supplement: Supplementary file 2 [file NIHMS1783029-supplement-Fig__S10.pdf]

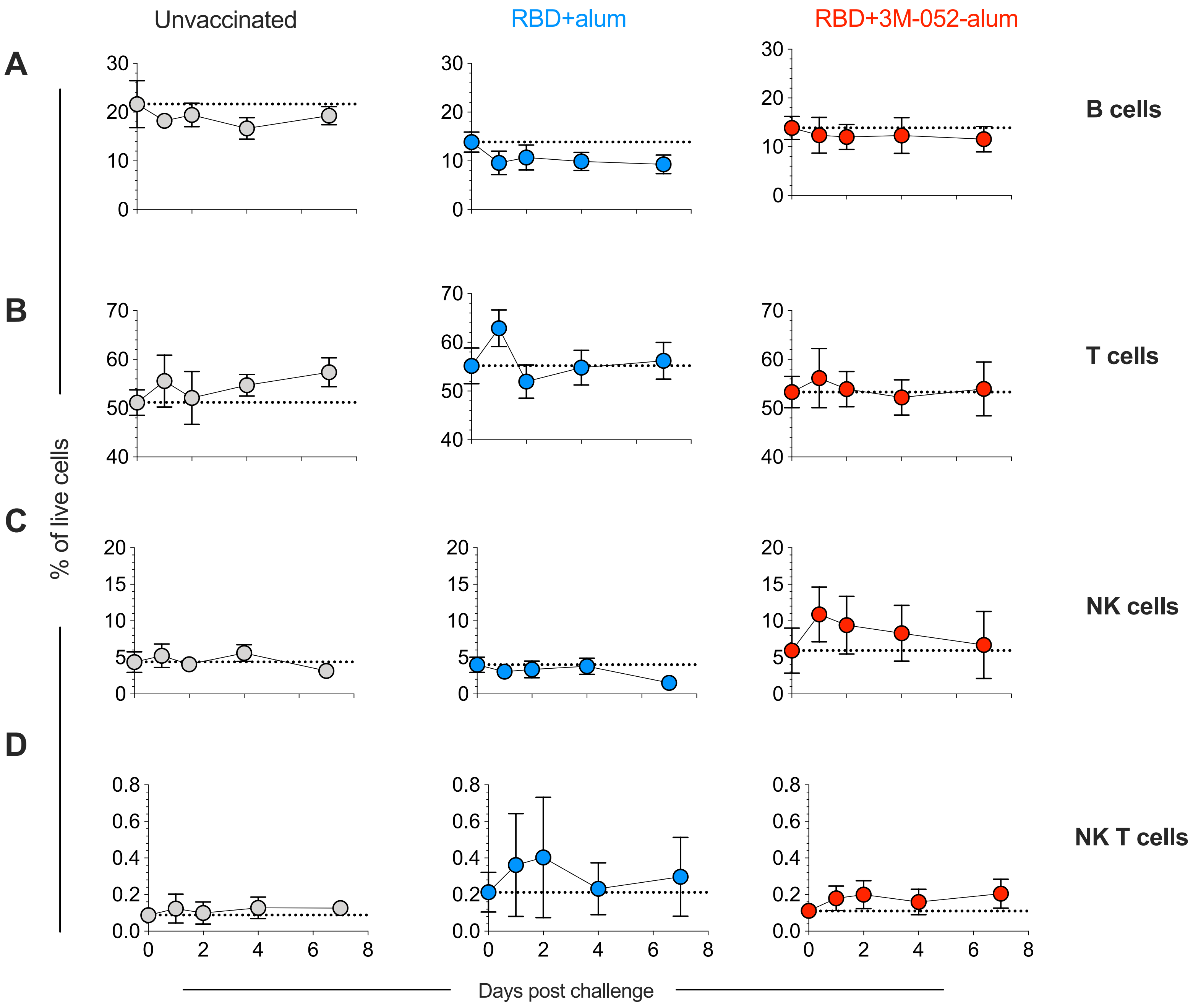

Unvaccinated
RBD+alum
RBD+3M-052-alum
A
B
C
D
B cells
T cells
NK cells
NK T cells
% of live cells
Days post challenge
