# Supplementary material for: A yeast-expressed RBD-based SARS-CoV-2 vaccine formulated with 3M-052-alum adjuvant promotes protective efficacy in non-human primates
Source: Sci Immunol. Author manuscript; Available in PMC 2022 May 19. (PMC9119307; doi:10.1126/sciimmunol.abh3634)

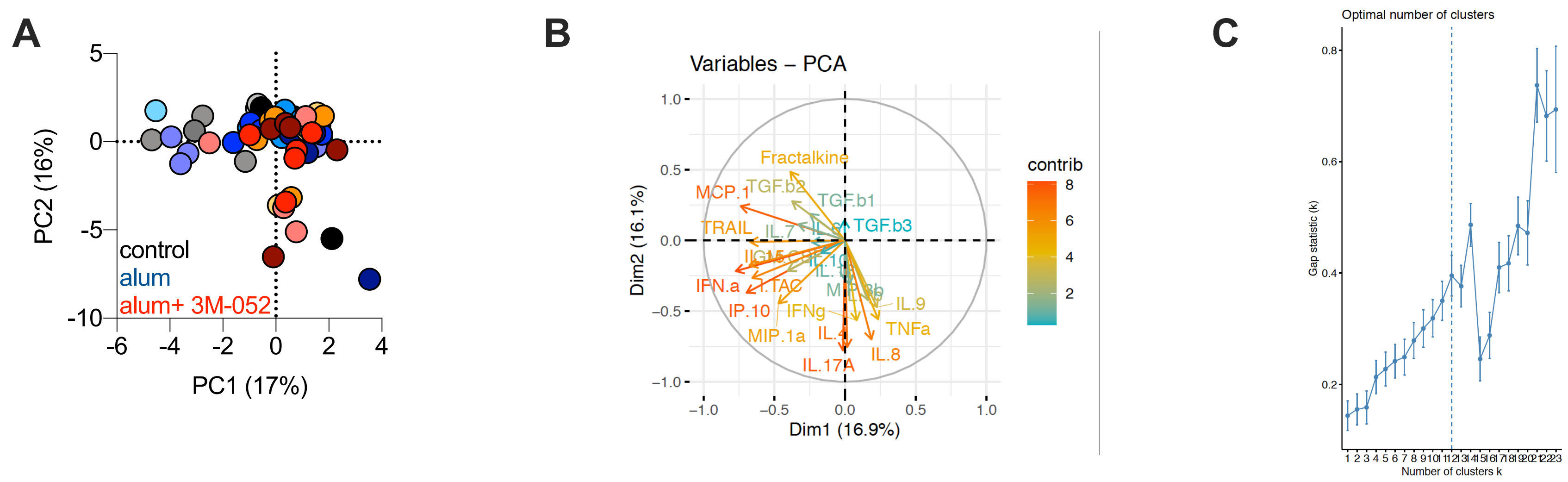

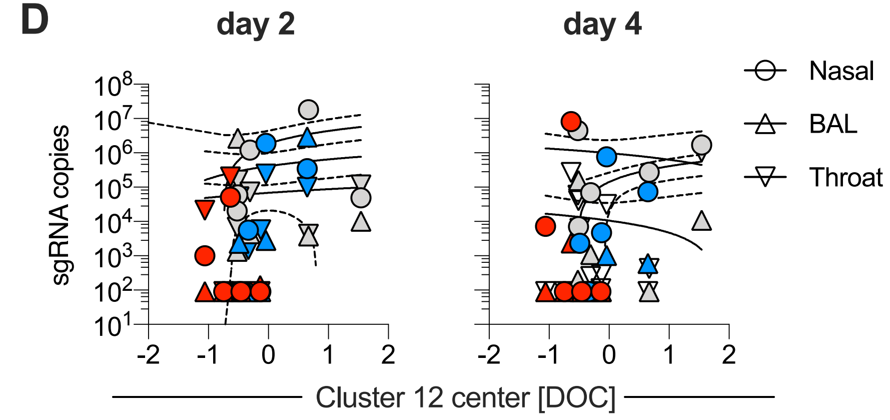

E

Correlation	Spearman r	P (two-tailed)	significant ?
Cluster center, DOC vs. sgRNA nasal d2	0.411	0.1293	n.s
Cluster center, DOC vs. sgRNA BAL d2	0.5533	0.035	yes
Cluster center, DOC vs. sgRNA throat d2	0.129	0.6467	n.s
Cluster center, DOC vs. sgRNA nasal d4	0.1856	0.5043	n.s
Cluster center, DOC vs. sgRNA BAL d4	0.1375	0.622	n.s
Cluster center, DOC vs. sgRNA throat d4	0.1491	0.5949	n.s

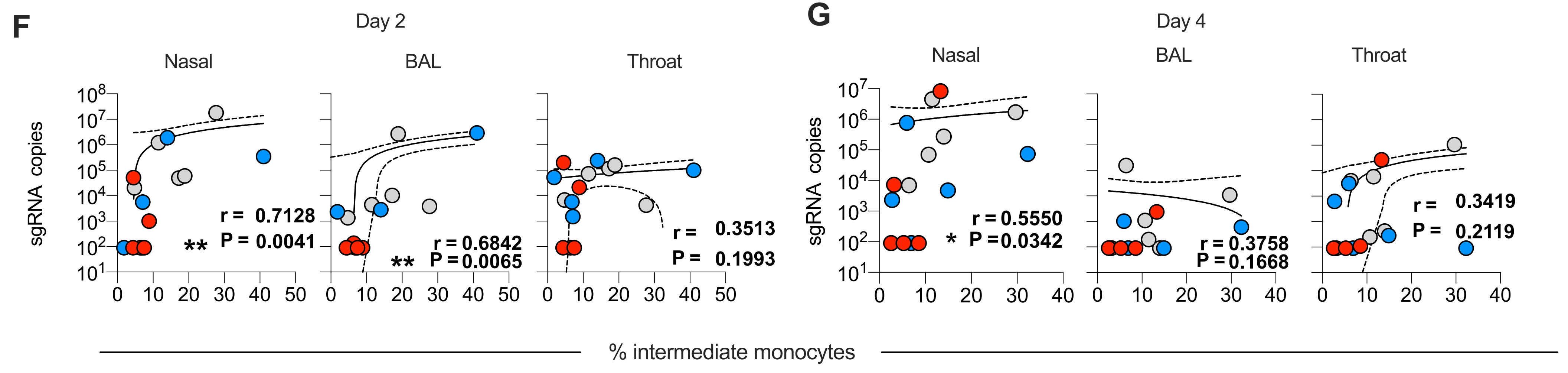

Supplement: Supplementary file 3 [file NIHMS1783029-supplement-Fig__S11.pdf]
