# Supplementary material for: A yeast-expressed RBD-based SARS-CoV-2 vaccine formulated with 3M-052-alum adjuvant promotes protective efficacy in non-human primates
Source: Sci Immunol. Author manuscript; Available in PMC 2022 May 19. (PMC9119307; doi:10.1126/sciimmunol.abh3634)
Supplement: Supplementary file 4 [file NIHMS1783029-supplement-Fig__S12.pdf]

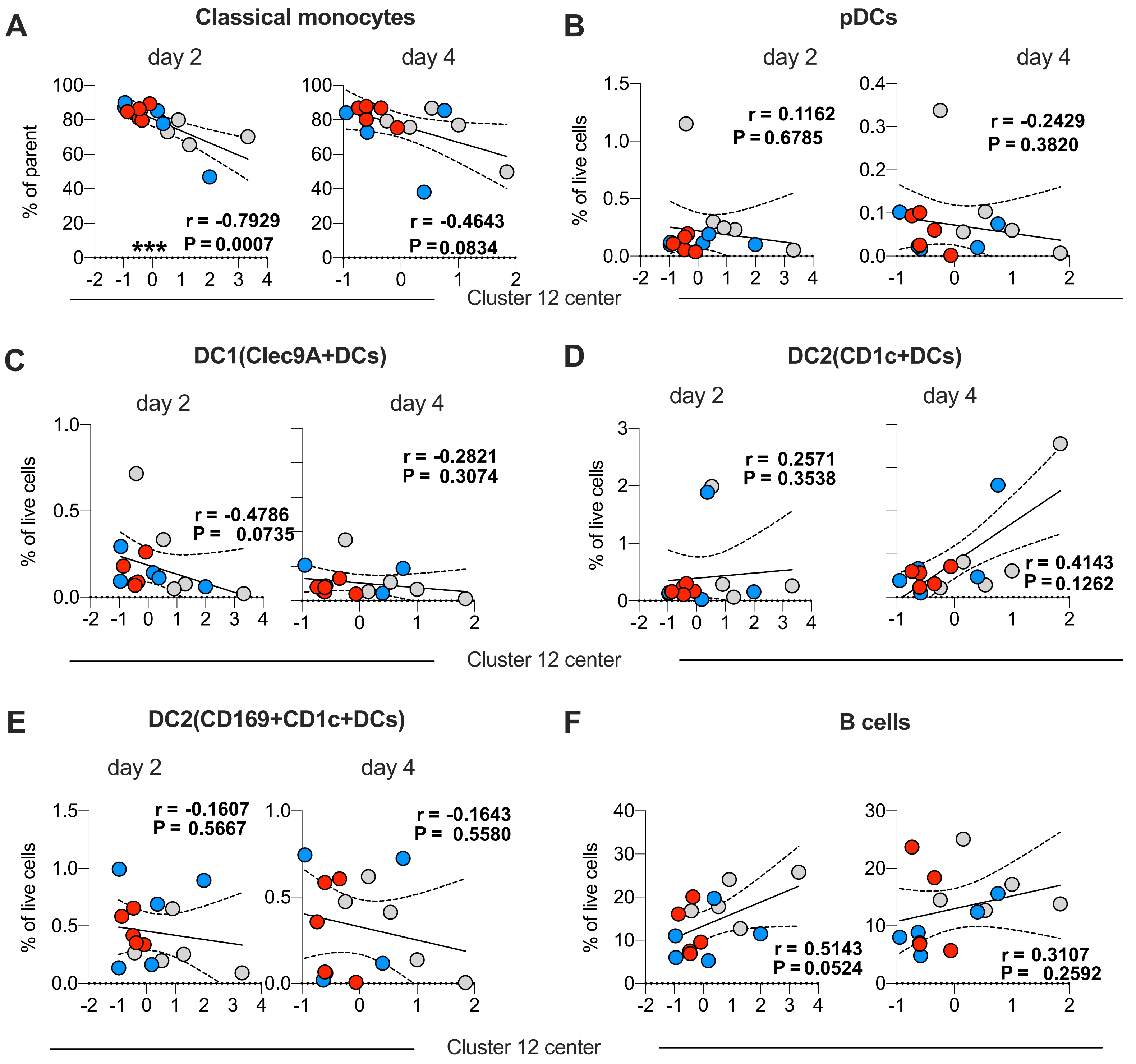
A
Classical monocytes
day 2
day 4
% of parent
r = -0.7929
P = 0.0007
***
r = -0.4643
P = 0.0834
B
pDCs
day 2
day 4
% of live cells
r = 0.1162
P = 0.6785
r = -0.2429
P = 0.3820
Cluster 12 center
C
DC1(Clec9A+DCs)
day 2
day 4
% of live cells
r = -0.4786
P = 0.0735
r = -0.2821
P = 0.3074
D
DC2(CD1c+DCs)
day 2
day 4
% of live cells
r = 0.2571
P = 0.3538
r = 0.4143
P = 0.1262
Cluster 12 center
E
DC2(CD169+CD1c+DCs)
day 2
day 4
% of live cells
r = -0.1607
P = 0.5667
r = -0.1643
P = 0.5580
F
B cells
% of live cells
r = 0.5143
P = 0.0524
r = 0.3107
P = 0.2592
Cluster 12 center
