# Supplementary material for: A yeast-expressed RBD-based SARS-CoV-2 vaccine formulated with 3M-052-alum adjuvant promotes protective efficacy in non-human primates
Source: Sci Immunol. Author manuscript; Available in PMC 2022 May 19. (PMC9119307; doi:10.1126/sciimmunol.abh3634)
Supplement: Supplementary file 5 [file NIHMS1783029-supplement-Fig__S13.pdf]

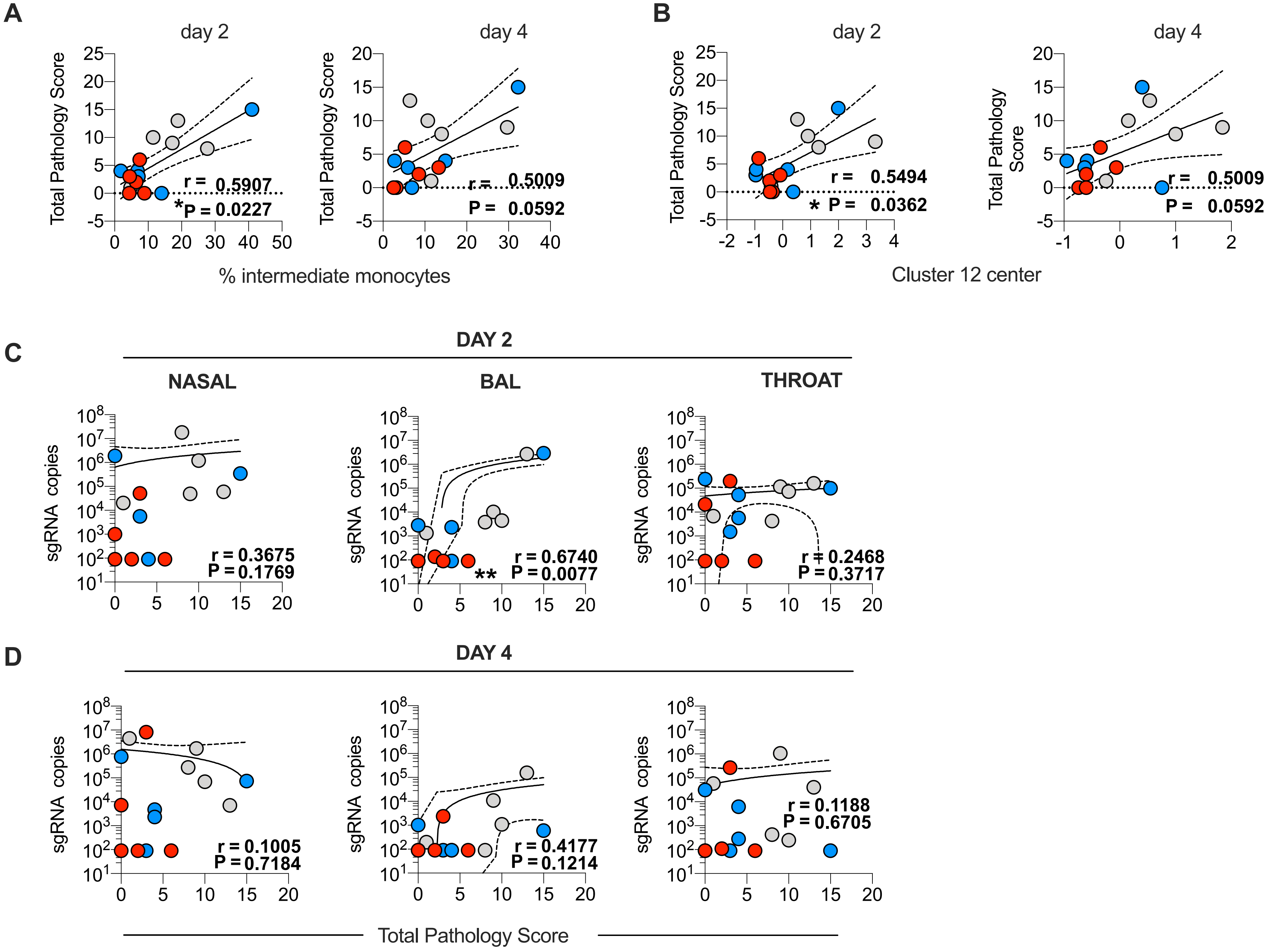
A
day 2
day 4
Total Pathology Score
r = 0.5907
*P = 0.0227
r = 0.5009
P = 0.0592
% intermediate monocytes
B
day 2
day 4
Total Pathology Score
r = 0.5494
* P = 0.0362
r = 0.5009
P = 0.0592
Cluster 12 center
C
DAY 2
NASAL
BAL
THROAT
sgRNA copies
r = 0.3675
P = 0.1769
r = 0.6740
** P = 0.0077
r = 0.2468
P = 0.3717
D
DAY 4
r = 0.1005
P = 0.7184
r = 0.4177
P = 0.1214
r = 0.1188
P = 0.6705
Total Pathology Score
