# Supplementary material for: A yeast-expressed RBD-based SARS-CoV-2 vaccine formulated with 3M-052-alum adjuvant promotes protective efficacy in non-human primates
Source: Sci Immunol. Author manuscript; Available in PMC 2022 May 19. (PMC9119307; doi:10.1126/sciimmunol.abh3634)
Supplement: Supplementary file 6 [file NIHMS1783029-supplement-Fig__S14.pdf]

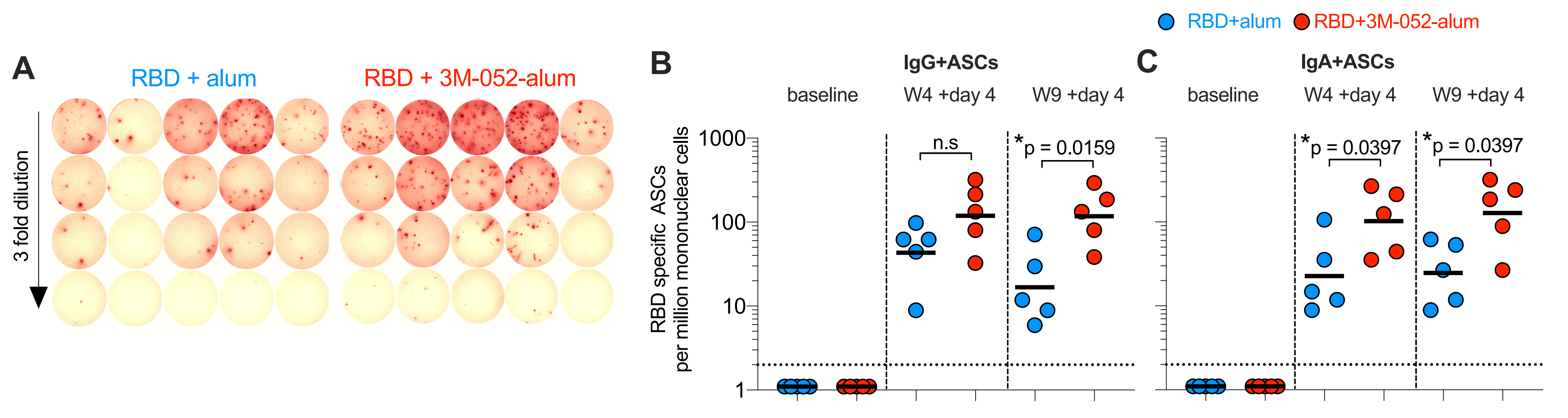
RBD+alum
RBD+3M-052-alum
A
RBD + alum
RBD + 3M-052-alum
3 fold dilution
B
IgG+ASCs
baseline
W4 +day 4
W9 +day 4
n.s
*p = 0.0159
RBD specific ASCs per million mononuclear cells
1000
100
10
1
C
IgA+ASCs
baseline
W4 +day 4
W9 +day 4
*p = 0.0397
*p = 0.0397
