# Supplementary material for: A yeast-expressed RBD-based SARS-CoV-2 vaccine formulated with 3M-052-alum adjuvant promotes protective efficacy in non-human primates
Source: Sci Immunol. Author manuscript; Available in PMC 2022 May 19. (PMC9119307; doi:10.1126/sciimmunol.abh3634)
Supplement: Supplementary file 7 [file NIHMS1783029-supplement-Fig__S15.pdf]

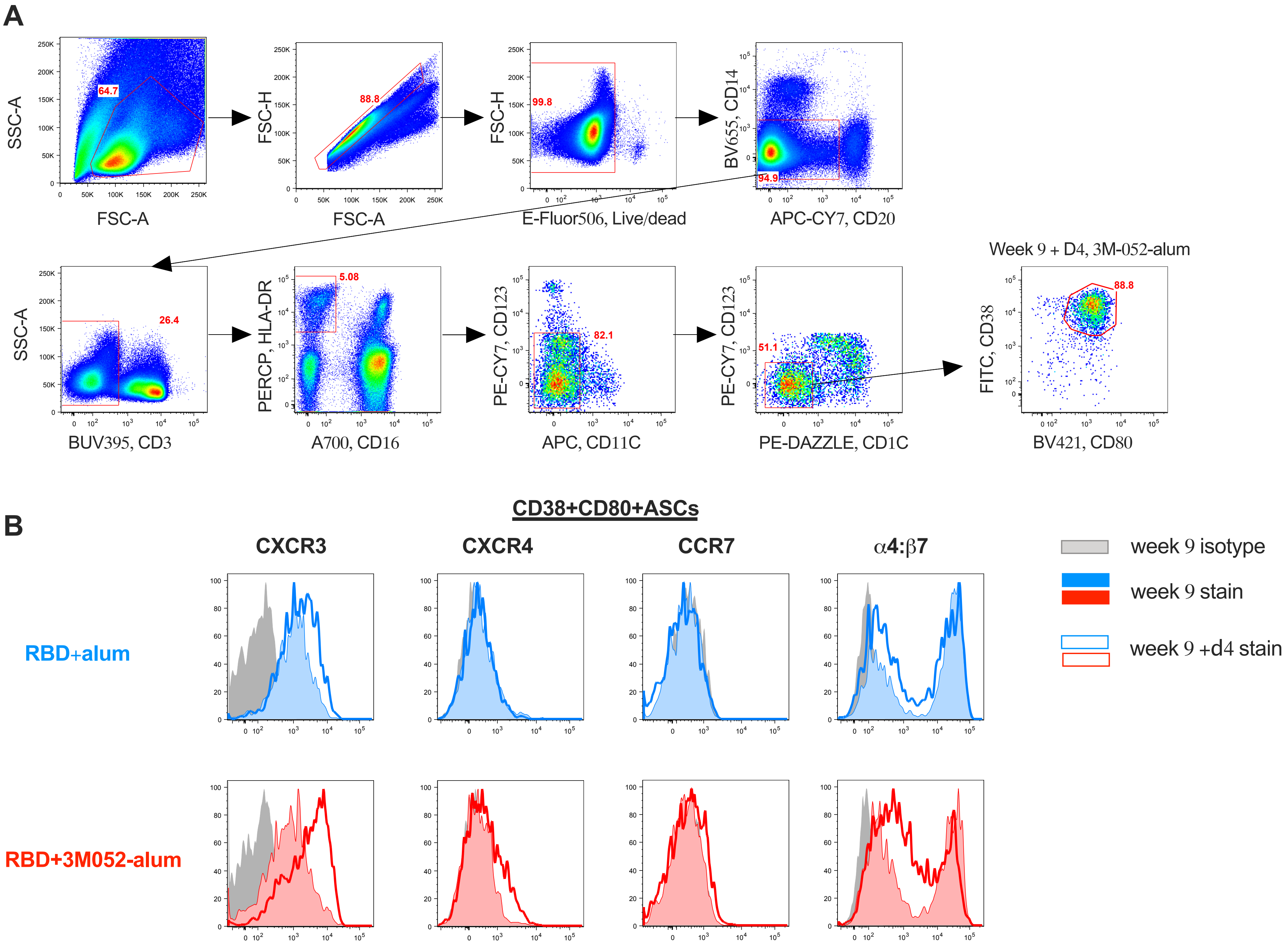
A
SSC-A
FSC-A
64.7
FSC-H
FSC-A
88.8
FSC-H
E-Fluor506, Live/dead
99.8
BV655, CD14
APC-CY7, CD20
94.9
SSC-A
BUV395, CD3
26.4
PERCP, HLA-DR
A700, CD16
5.08
PE-CY7, CD123
APC, CD11C
82.1
PE-CY7, CD123
PE-DAZZLE, CD1C
51.1
Week 9 + D4, 3M-052-alum
FITC, CD38
BV421, CD80
88.8
B
CD38+CD80+ASCs
CXCR3
CXCR4
CCR7
α4:β7
week 9 isotype
week 9 stain
week 9 +d4 stain
RBD+alum
RBD+3M052-alum
