# Supplementary material for: A yeast-expressed RBD-based SARS-CoV-2 vaccine formulated with 3M-052-alum adjuvant promotes protective efficacy in non-human primates
Source: Sci Immunol. Author manuscript; Available in PMC 2022 May 19. (PMC9119307; doi:10.1126/sciimmunol.abh3634)
Supplement: Supplementary file 8 [file NIHMS1783029-supplement-Fig__S16.pdf]

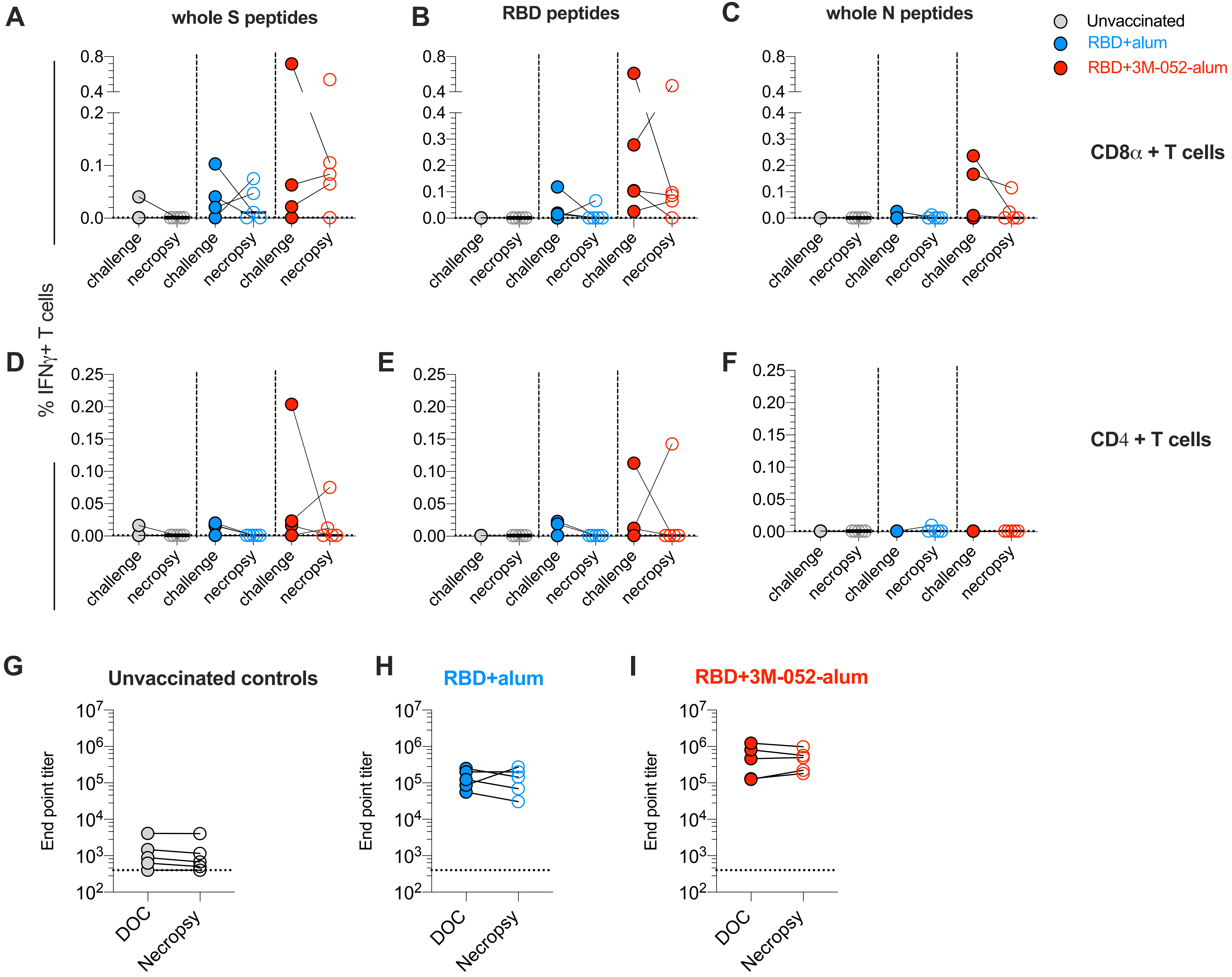
A
whole S peptides
B
RBD peptides
C
whole N peptides
Unvaccinated
RBD+alum
RBD+3M-052-alum
CD8α + T cells
% IFNγ+ T cells
challenge
necropsy
D
E
F
CD4 + T cells
G
Unvaccinated controls
H
RBD+alum
I
RBD+3M-052-alum
End point titer
DOC
Necropsy
