# Supplementary material for: A yeast-expressed RBD-based SARS-CoV-2 vaccine formulated with 3M-052-alum adjuvant promotes protective efficacy in non-human primates
Source: Sci Immunol. Author manuscript; Available in PMC 2022 May 19. (PMC9119307; doi:10.1126/sciimmunol.abh3634)
Supplement: Supplementary file 9 [file NIHMS1783029-supplement-Fig__S17.pdf]

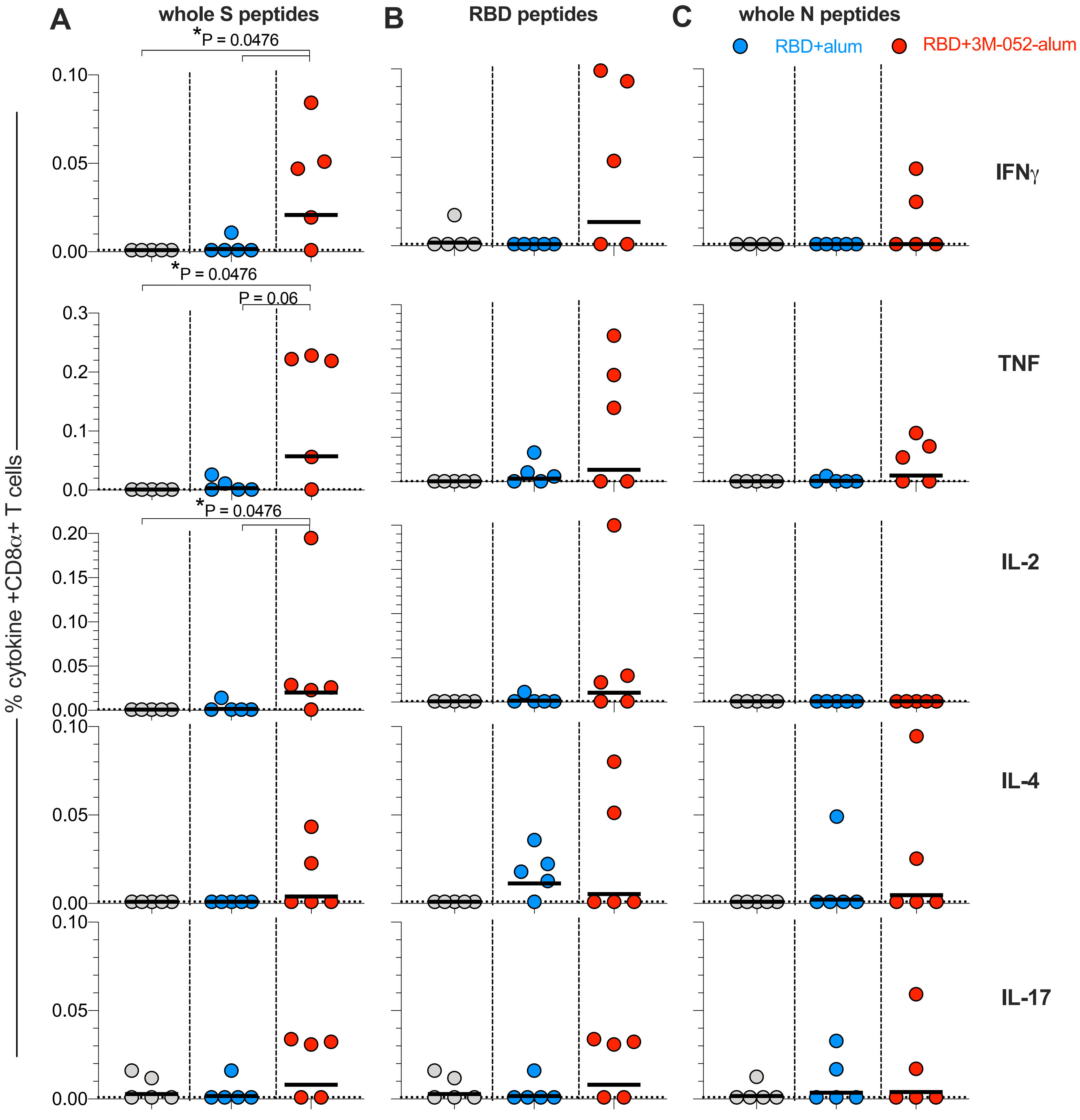
A
whole S peptides
B
RBD peptides
C
whole N peptides
RBD+alum
RBD+3M-052-alum
*P = 0.0476
*P = 0.0476
P = 0.06
*P = 0.0476
IFNγ
TNF
IL-2
IL-4
IL-17
% cytokine +CD8α+ T cells
