# Supplementary material for: A yeast-expressed RBD-based SARS-CoV-2 vaccine formulated with 3M-052-alum adjuvant promotes protective efficacy in non-human primates
Source: Sci Immunol. Author manuscript; Available in PMC 2022 May 19. (PMC9119307; doi:10.1126/sciimmunol.abh3634)
Supplement: Supplementary file 10 [file NIHMS1783029-supplement-Fig__S18.pdf]

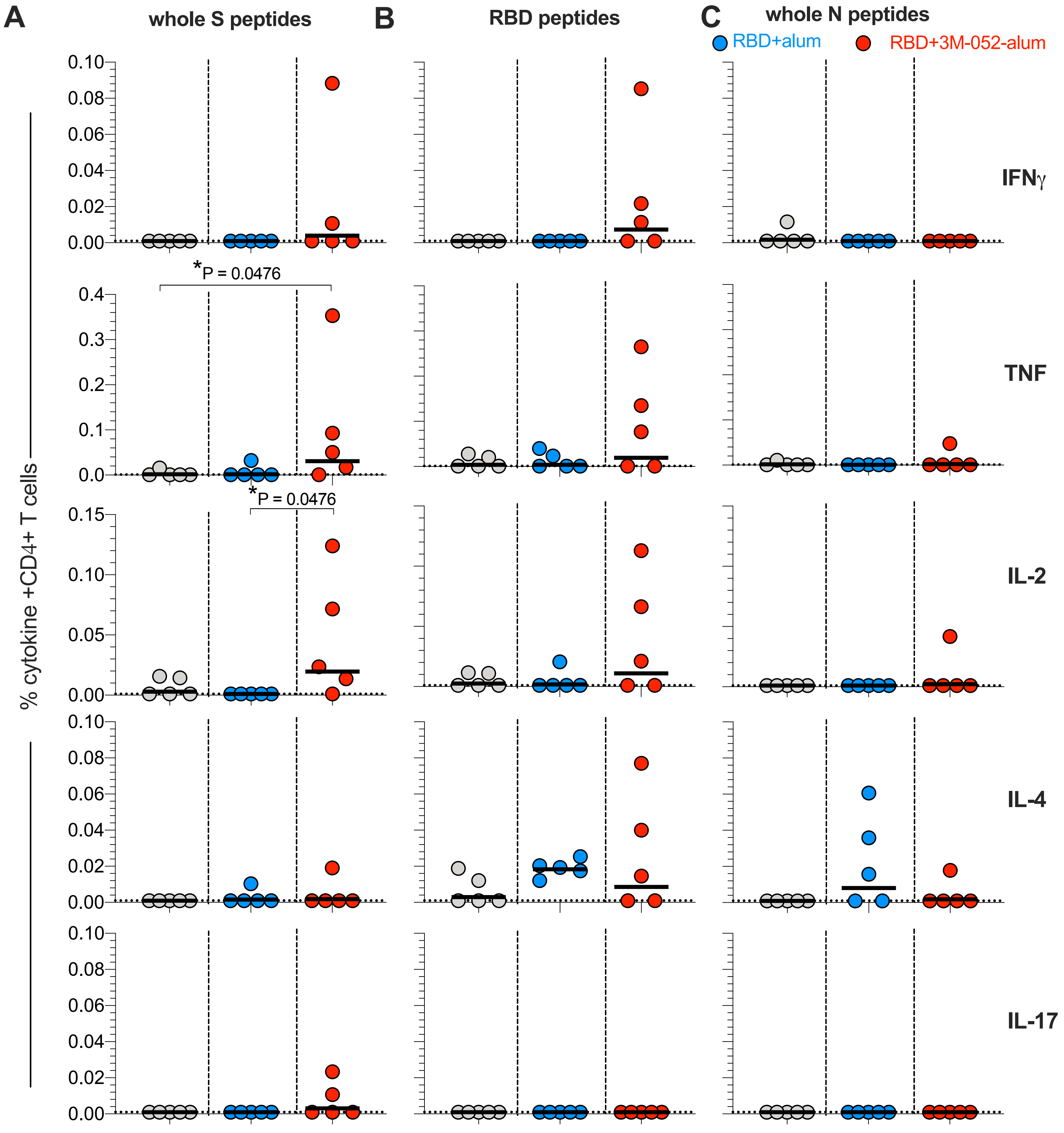

A
whole S peptides
B
RBD peptides
C
whole N peptides
RBD+alum
RBD+3M-052-alum
IFNγ
TNF
IL-2
IL-4
IL-17
% cytokine +CD4+ T cells
*P = 0.0476
*P = 0.0476
0.10
0.08
0.06
0.04
0.02
0.00
0.4
0.3
0.2
0.1
0.0
0.15
0.10
0.05
0.00
