# Supplementary material for: A yeast-expressed RBD-based SARS-CoV-2 vaccine formulated with 3M-052-alum adjuvant promotes protective efficacy in non-human primates
Source: Sci Immunol. Author manuscript; Available in PMC 2022 May 19. (PMC9119307; doi:10.1126/sciimmunol.abh3634)
Supplement: Supplementary file 11 [file NIHMS1783029-supplement-Fig__S2.pdf]

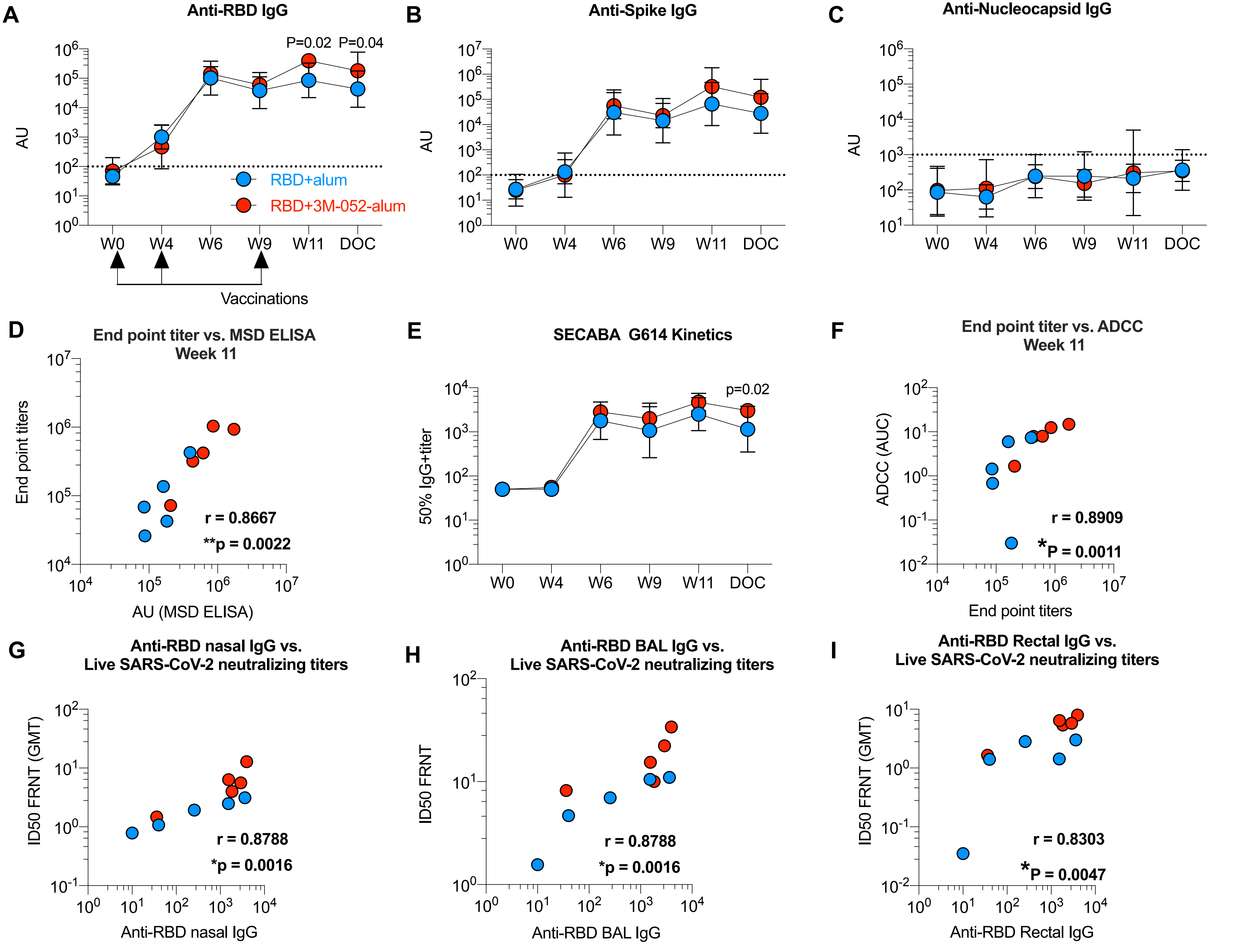

A
Anti-RBD IgG
P=0.02
P=0.04
AU
RBD+alum
RBD+3M-052-alum
W0
W4
W6
W9
W11
DOC
Vaccinations
B
Anti-Spike IgG
C
Anti-Nucleocapsid IgG
D
End point titer vs. MSD ELISA
Week 11
End point titers
AU (MSD ELISA)
r = 0.8667
**p = 0.0022
E
SECABA G614 Kinetics
p=0.02
50% IgG+titer
F
End point titer vs. ADCC
Week 11
ADCC (AUC)
End point titers
r = 0.8909
*P = 0.0011
G
Anti-RBD nasal IgG vs.
Live SARS-CoV-2 neutralizing titers
ID50 FRNT (GMT)
Anti-RBD nasal IgG
r = 0.8788
*p = 0.0016
H
Anti-RBD BAL IgG vs.
Live SARS-CoV-2 neutralizing titers
ID50 FRNT
Anti-RBD BAL IgG
r = 0.8788
*p = 0.0016
I
Anti-RBD Rectal IgG vs.
Live SARS-CoV-2 neutralizing titers
ID50 FRNT (GMT)
Anti-RBD Rectal IgG
r = 0.8303
*P = 0.0047
