# Supplementary material for: A yeast-expressed RBD-based SARS-CoV-2 vaccine formulated with 3M-052-alum adjuvant promotes protective efficacy in non-human primates
Source: Sci Immunol. Author manuscript; Available in PMC 2022 May 19. (PMC9119307; doi:10.1126/sciimmunol.abh3634)
Supplement: Supplementary file 12 [file NIHMS1783029-supplement-Fig__S3.pdf]

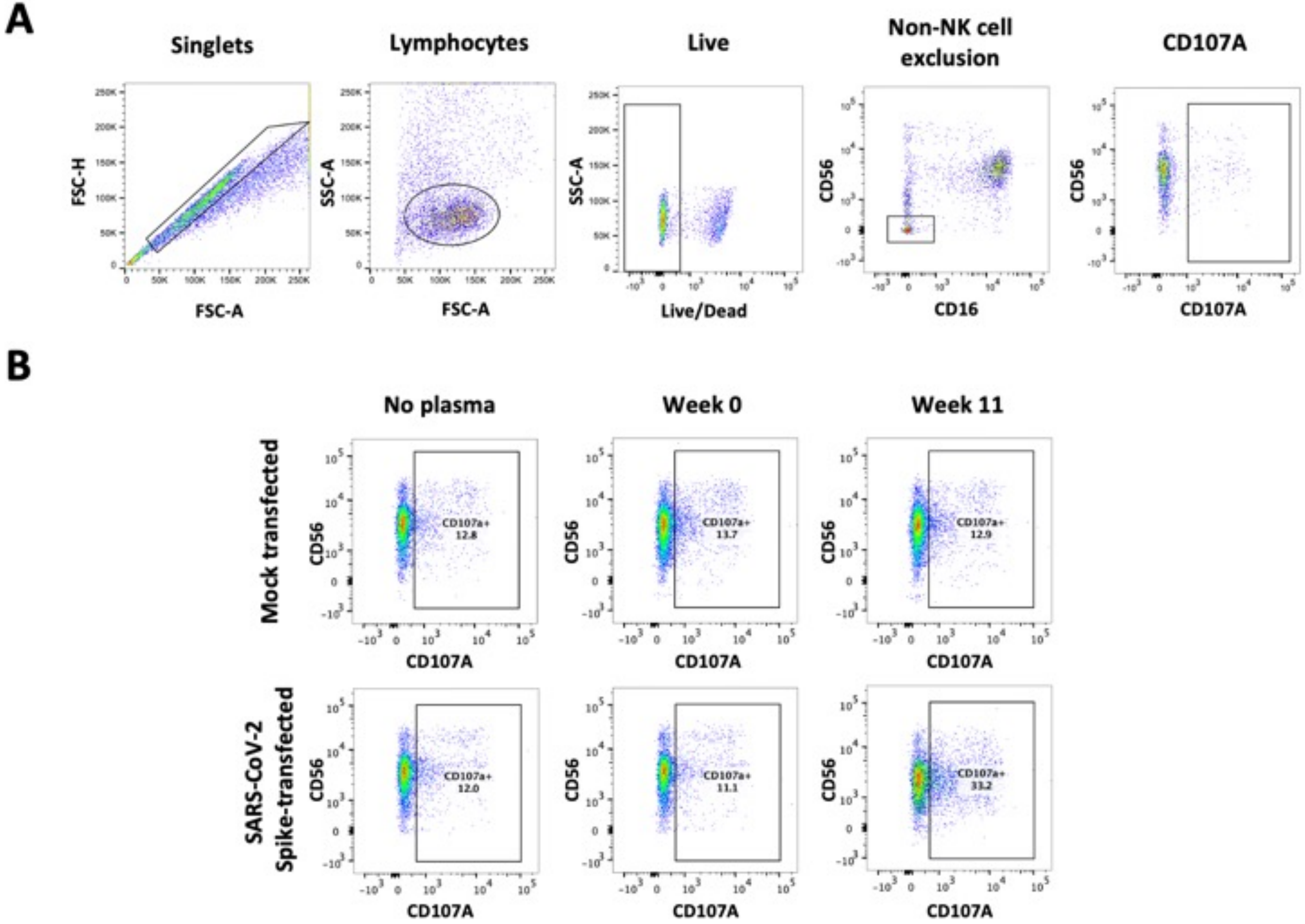

A
Singlets
Lymphocytes
Live
Non-NK cell exclusion
CD107A
FSC-H
FSC-A
SSC-A
FSC-A
SSC-A
Live/Dead
CD56
CD16
CD56
CD107A
B
No plasma
Week 0
Week 11
Mock transfected
SARS-CoV-2 Spike-transfected
CD107a+ 12.8
CD107a+ 13.7
CD107a+ 12.9
CD107a+ 12.0
CD107a+ 11.1
CD107a+ 33.2
CD56
CD107A
