# Supplementary material for: A yeast-expressed RBD-based SARS-CoV-2 vaccine formulated with 3M-052-alum adjuvant promotes protective efficacy in non-human primates
Source: Sci Immunol. Author manuscript; Available in PMC 2022 May 19. (PMC9119307; doi:10.1126/sciimmunol.abh3634)
Supplement: Supplementary file 13 [file NIHMS1783029-supplement-Fig__S4.pdf]

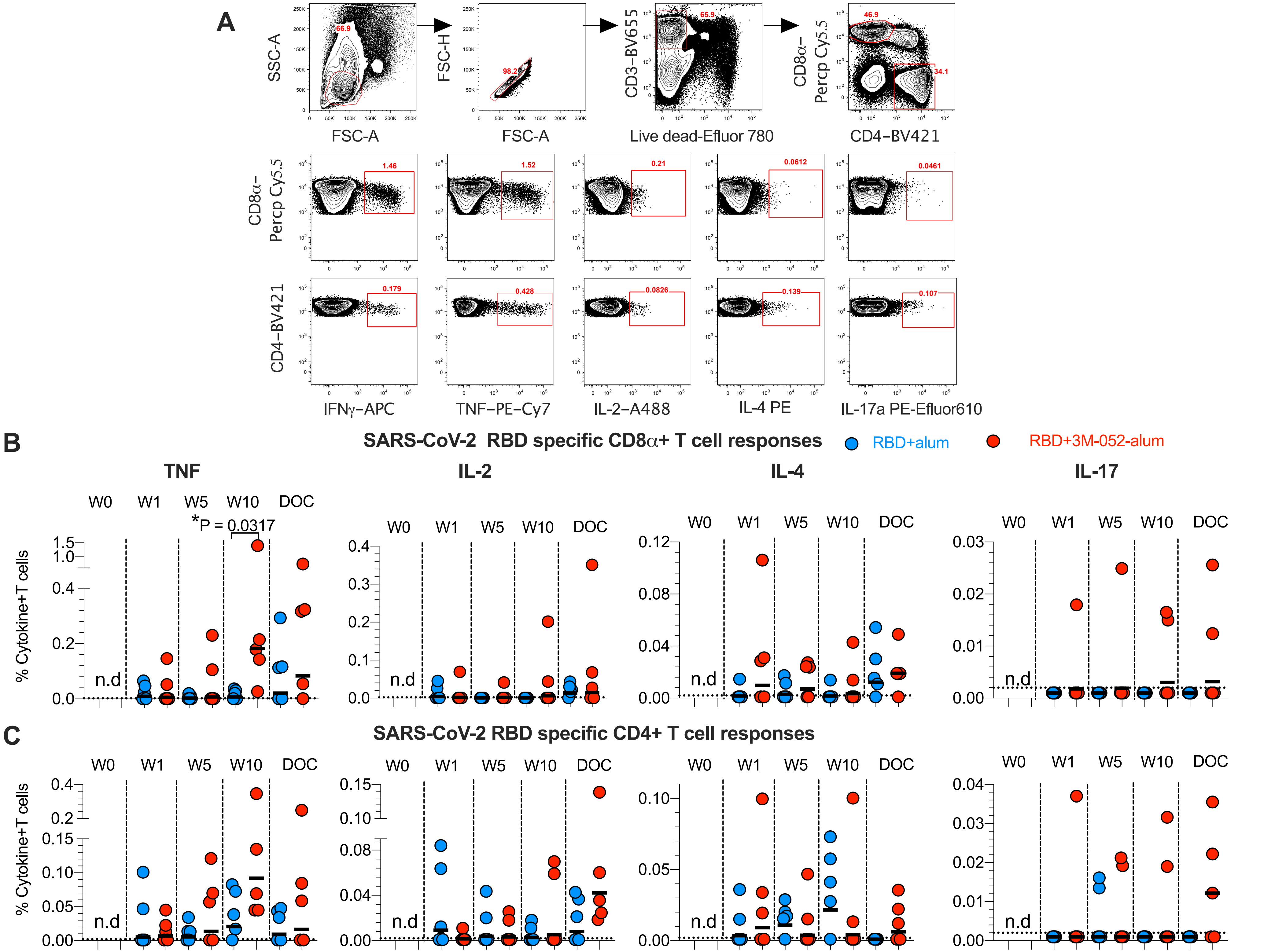

A
SSC-A
FSC-A
66.9
FSC-H
98.2
CD3-BV655
Live dead-Efluor 780
65.9
CD8α-Percp Cy5.5
CD4-BV421
46.9
34.1
1.46
1.52
0.21
0.0612
0.0461
0.179
0.428
0.0826
0.139
0.107
IFNγ-APC
TNF-PE-Cy7
IL-2-A488
IL-4 PE
IL-17a PE-Efluor610
B
SARS-CoV-2 RBD specific CD8α+ T cell responses
RBD+alum
RBD+3M-052-alum
TNF
IL-2
IL-4
IL-17
W0
W1
W5
W10
DOC
*P = 0.0317
% Cytokine+T cells
n.d
C
SARS-CoV-2 RBD specific CD4+ T cell responses
