# Supplementary material for: A yeast-expressed RBD-based SARS-CoV-2 vaccine formulated with 3M-052-alum adjuvant promotes protective efficacy in non-human primates
Source: Sci Immunol. Author manuscript; Available in PMC 2022 May 19. (PMC9119307; doi:10.1126/sciimmunol.abh3634)
Supplement: Supplementary file 14 [file NIHMS1783029-supplement-Fig__S5.pdf]

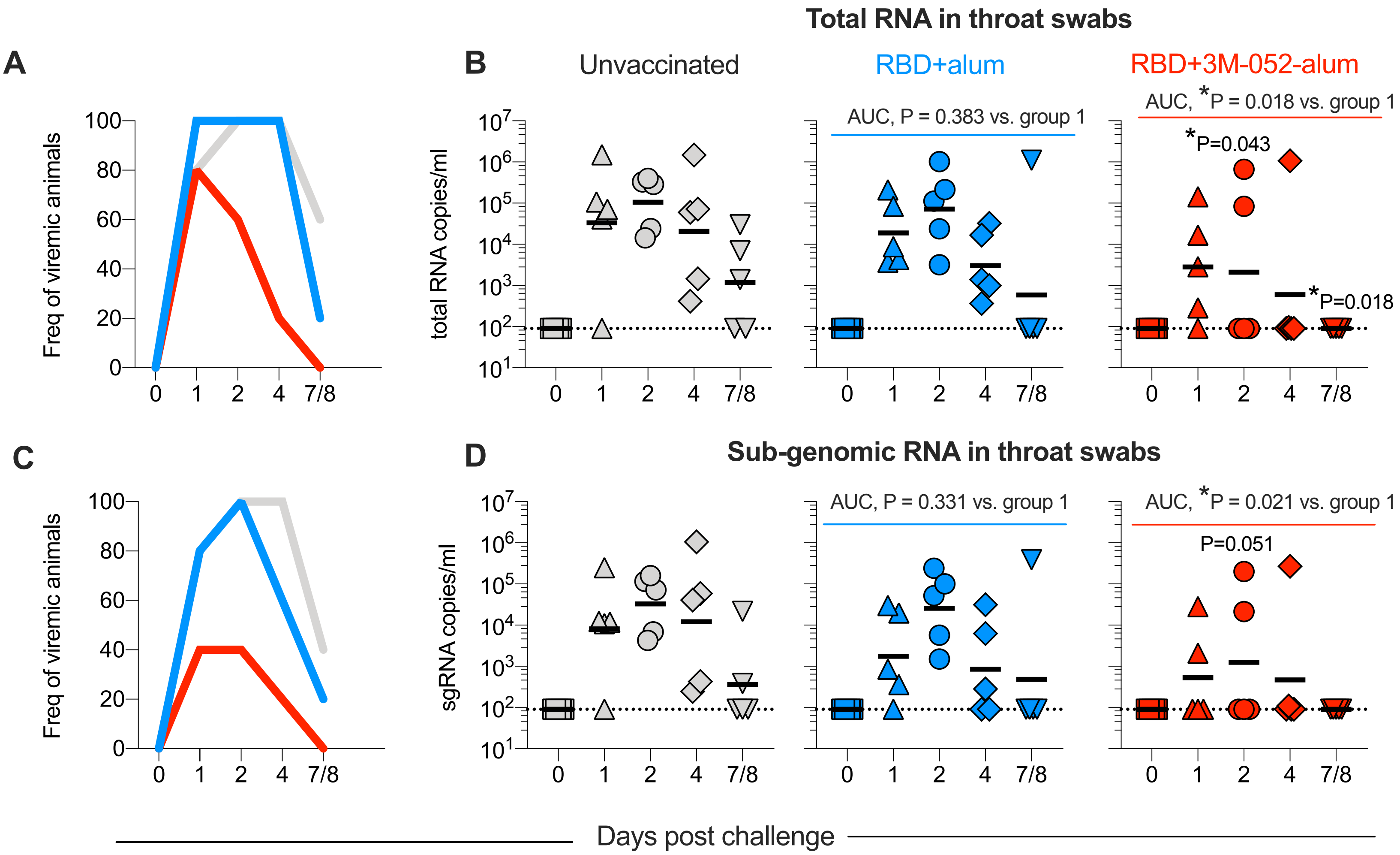

A
Freq of viremic animals
100
80
60
40
20
0
0
1
2
4
7/8
Total RNA in throat swabs
B
Unvaccinated
RBD+alum
RBD+3M-052-alum
total RNA copies/ml
10^7
10^6
10^5
10^4
10^3
10^2
10^1
AUC, P = 0.383 vs. group 1
AUC, *P = 0.018 vs. group 1
*P=0.043
*P=0.018
C
Sub-genomic RNA in throat swabs
D
sgRNA copies/ml
AUC, P = 0.331 vs. group 1
AUC, *P = 0.021 vs. group 1
P=0.051
Days post challenge
