# Supplementary material for: A yeast-expressed RBD-based SARS-CoV-2 vaccine formulated with 3M-052-alum adjuvant promotes protective efficacy in non-human primates
Source: Sci Immunol. Author manuscript; Available in PMC 2022 May 19. (PMC9119307; doi:10.1126/sciimmunol.abh3634)
Supplement: Supplementary file 15 [file NIHMS1783029-supplement-Fig__S7.pdf]

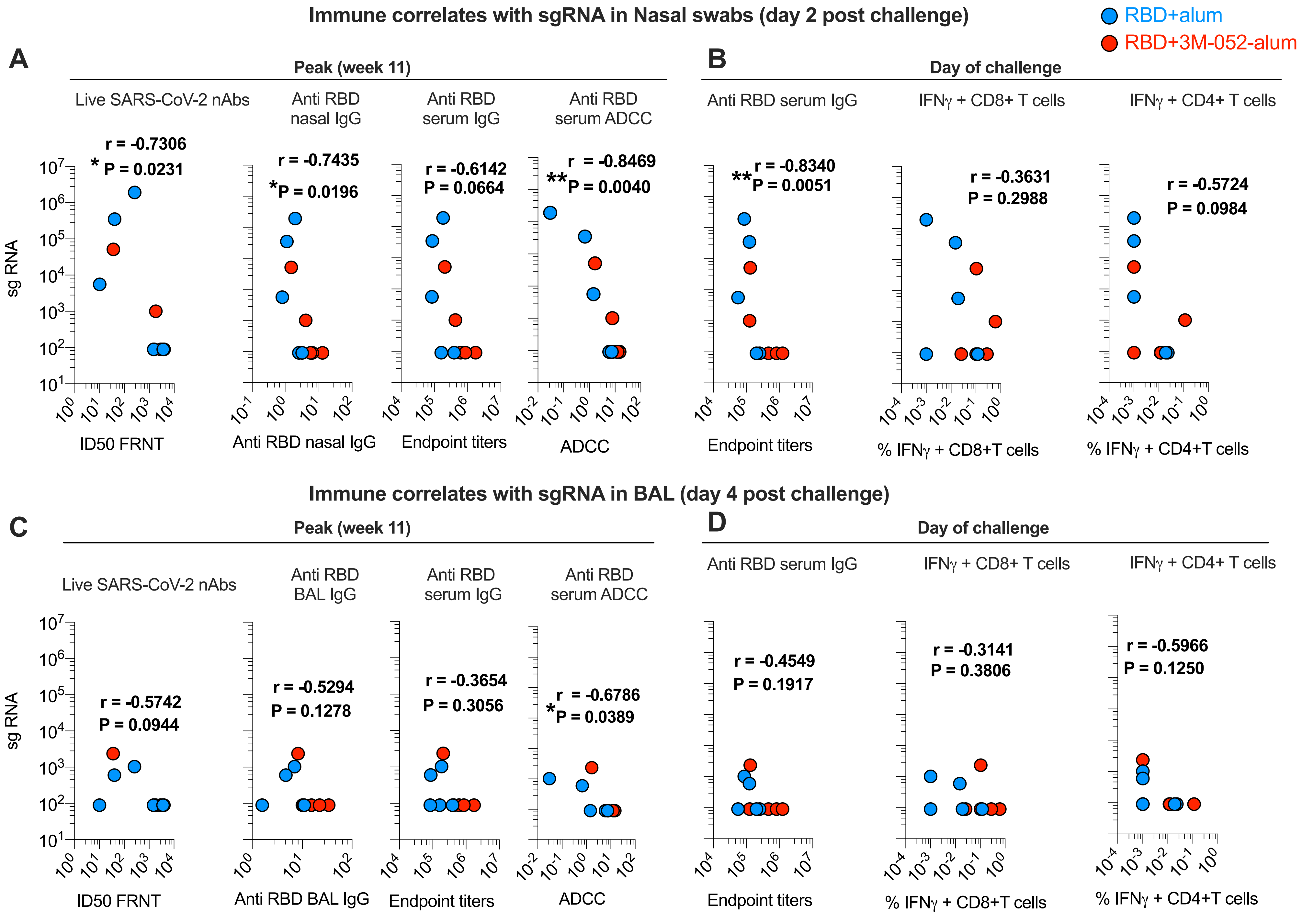

Immune correlates with sgRNA in Nasal swabs (day 2 post challenge)
RBD+alum
RBD+3M-052-alum
A
Peak (week 11)
Live SARS-CoV-2 nAbs
r = -0.7306
* P = 0.0231
Anti RBD nasal IgG
r = -0.7435
*P = 0.0196
Anti RBD serum IgG
r = -0.6142
P = 0.0664
Anti RBD serum ADCC
r = -0.8469
**P = 0.0040
sg RNA
ID50 FRNT
Anti RBD nasal IgG
Endpoint titers
ADCC
B
Day of challenge
Anti RBD serum IgG
**r = -0.8340
P = 0.0051
IFNγ + CD8+ T cells
r = -0.3631
P = 0.2988
IFNγ + CD4+ T cells
r = -0.5724
P = 0.0984
Endpoint titers
% IFNγ + CD8+T cells
% IFNγ + CD4+T cells
Immune correlates with sgRNA in BAL (day 4 post challenge)
C
Peak (week 11)
Live SARS-CoV-2 nAbs
r = -0.5742
P = 0.0944
Anti RBD BAL IgG
r = -0.5294
P = 0.1278
Anti RBD serum IgG
r = -0.3654
P = 0.3056
Anti RBD serum ADCC
r = -0.6786
*P = 0.0389
sg RNA
ID50 FRNT
Anti RBD BAL IgG
Endpoint titers
ADCC
D
Day of challenge
Anti RBD serum IgG
r = -0.4549
P = 0.1917
IFNγ + CD8+ T cells
r = -0.3141
P = 0.3806
IFNγ + CD4+ T cells
r = -0.5966
P = 0.1250
Endpoint titers
% IFNγ + CD8+T cells
% IFNγ + CD4+T cells
