# Supplementary material for: A yeast-expressed RBD-based SARS-CoV-2 vaccine formulated with 3M-052-alum adjuvant promotes protective efficacy in non-human primates
Source: Sci Immunol. Author manuscript; Available in PMC 2022 May 19. (PMC9119307; doi:10.1126/sciimmunol.abh3634)
Supplement: Supplementary file 16 [file NIHMS1783029-supplement-Fig__S8.pdf]

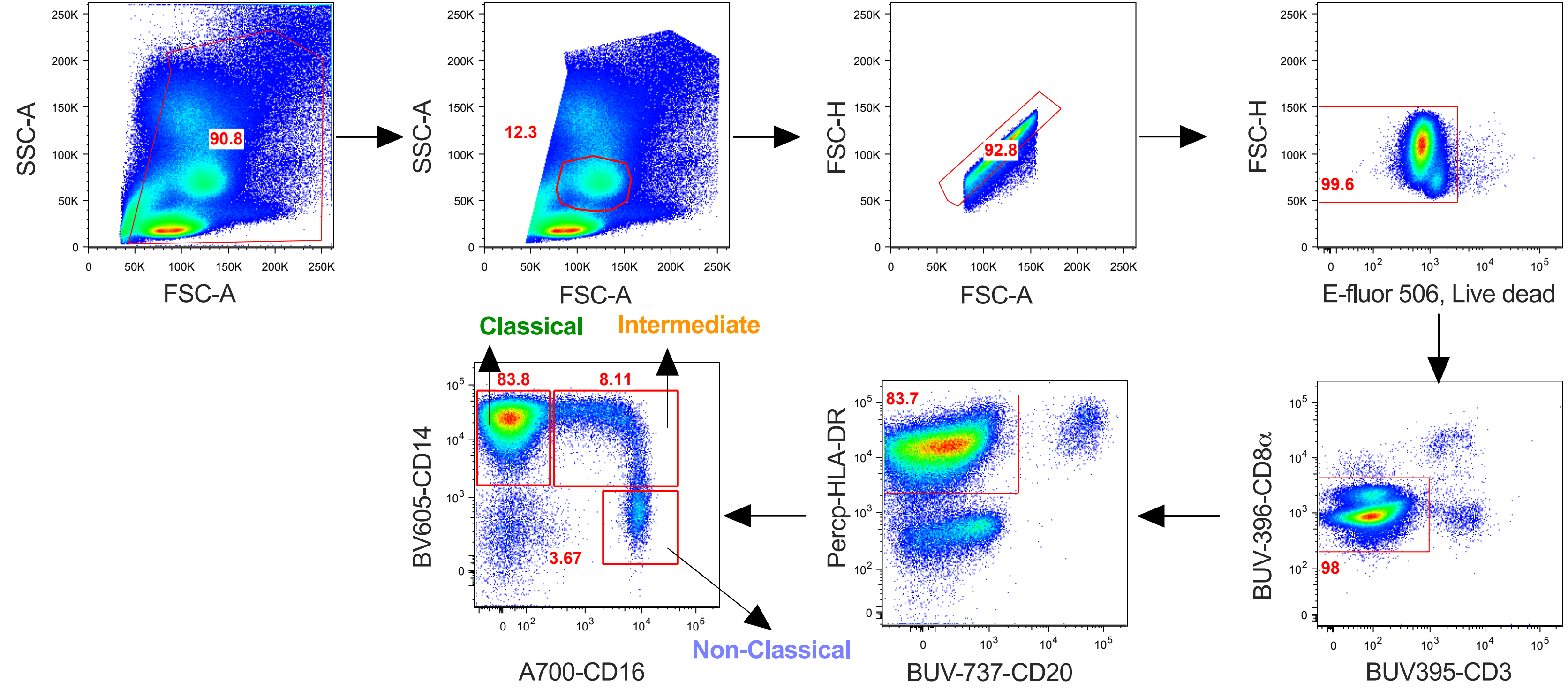

SSC-A
FSC-A
90.8
12.3
FSC-H
92.8
99.6
E-fluor 506, Live dead
BUV-396-CD8α
BUV395-CD3
98
Percp-HLA-DR
BUV-737-CD20
83.7
BV605-CD14
A700-CD16
83.8
8.11
3.67
Classical
Intermediate
Non-Classical
