# Supplementary material for: A yeast-expressed RBD-based SARS-CoV-2 vaccine formulated with 3M-052-alum adjuvant promotes protective efficacy in non-human primates
Source: Sci Immunol. Author manuscript; Available in PMC 2022 May 19. (PMC9119307; doi:10.1126/sciimmunol.abh3634)
Supplement: Supplementary file 17 [file NIHMS1783029-supplement-Fig__S9.pdf]

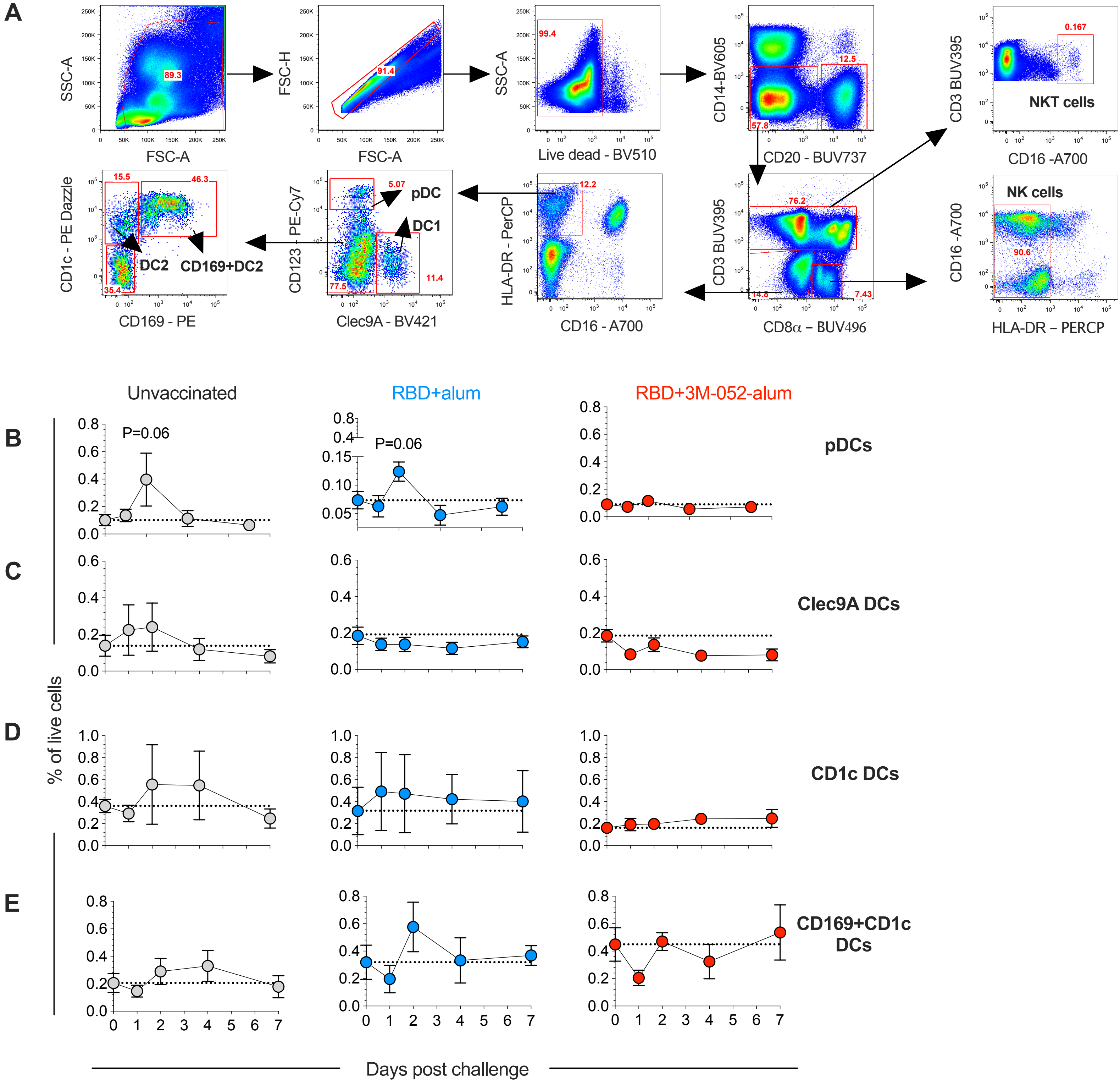

A
SSC-A
FSC-A
89.3
FSC-H
91.4
Live dead - BV510
99.4
CD14-BV605
CD20 - BUV737
57.8
12.5
CD3 BUV395
CD16 -A700
0.167
NKT cells
CD8α – BUV496
76.2
14.8
7.43
NK cells
HLA-DR – PERCP
90.6
HLA-DR - PerCP
CD16 - A700
12.2
CD123 - PE-Cy7
Clec9A - BV421
5.07
pDC
DC1
77.5
11.4
CD1c - PE Dazzle
CD169 - PE
15.5
46.3
35.4
DC2
CD169+DC2
Unvaccinated
RBD+alum
RBD+3M-052-alum
B
P=0.06
P=0.06
pDCs
C
Clec9A DCs
D
CD1c DCs
E
CD169+CD1c DCs
% of live cells
Days post challenge
